# Nut consumption and risk of cardiovascular disease, total cancer, all-cause and cause-specific mortality: a systematic review and dose-response meta-analysis of prospective studies

**DOI:** 10.1186/s12916-016-0730-3

**Published:** 2016-12-05

**Authors:** Dagfinn Aune, NaNa Keum, Edward Giovannucci, Lars T. Fadnes, Paolo Boffetta, Darren C. Greenwood, Serena Tonstad, Lars J. Vatten, Elio Riboli, Teresa Norat

**Affiliations:** 1 Department of Public Health and General Practice, Faculty of Medicine, Norwegian University of Science and Technology, Trondheim, Norway; 2 Department of Epidemiology and Biostatistics, School of Public Health, Imperial College London, London, UK; 3 Department of Nutrition, Harvard T.H. Chan School of Public Health, Boston, USA; 4 Department of Epidemiology, Harvard T.H. Chan School of Public Health, Boston, USA; 5 Channing Division of Network Medicine, Department of Medicine, Brigham and Women’s Hospital and Harvard Medical School, Boston, USA; 6 Centre for International Health, Department of Global Public Health and Primary Care & Department of Clinical Dentistry, University of Bergen, Bergen, Norway; 7 The Tisch Cancer Institute, Icahn School of Medicine at Mount Sinai, New York, USA; 8 Biostatistics Unit, Centre for Epidemiology and Biostatistics, University of Leeds, Leeds, UK; 9 Department of Preventive Cardiology, Oslo University Hospital Ullevål, Oslo, Norway

**Keywords:** Nuts, Peanuts, Cardiovascular disease, Cancer, All-cause mortality, Cause-specific mortality, Meta-analysis

## Abstract

**Background:**

Although nut consumption has been associated with a reduced risk of cardiovascular disease and all-cause mortality, data on less common causes of death has not been systematically assessed. Previous reviews missed several studies and additional studies have since been published. We therefore conducted a systematic review and meta-analysis of nut consumption and risk of cardiovascular disease, total cancer, and all-cause and cause-specific mortality.

**Methods:**

PubMed and Embase were searched for prospective studies of nut consumption and risk of cardiovascular disease, total cancer, and all-cause and cause-specific mortality in adult populations published up to July 19, 2016. Summary relative risks (RRs) and 95% confidence intervals (CIs) were calculated using random-effects models. The burden of mortality attributable to low nut consumption was calculated for selected regions.

**Results:**

Twenty studies (29 publications) were included in the meta-analysis. The summary RRs per 28 grams/day increase in nut intake was for coronary heart disease, 0.71 (95% CI: 0.63–0.80, I^2^ = 47%, *n* = 11), stroke, 0.93 (95% CI: 0.83–1.05, I^2^ = 14%, *n* = 11), cardiovascular disease, 0.79 (95% CI: 0.70–0.88, I^2^ = 60%, *n* = 12), total cancer, 0.85 (95% CI: 0.76–0.94, I^2^ = 42%, *n* = 8), all-cause mortality, 0.78 (95% CI: 0.72–0.84, I^2^ = 66%, *n* = 15), and for mortality from respiratory disease, 0.48 (95% CI: 0.26–0.89, I^2^ = 61%, *n* = 3), diabetes, 0.61 (95% CI: 0.43–0.88, I^2^ = 0%, *n* = 4), neurodegenerative disease, 0.65 (95% CI: 0.40–1.08, I^2^ = 5.9%, *n* = 3), infectious disease, 0.25 (95% CI: 0.07–0.85, I^2^ = 54%, *n* = 2), and kidney disease, 0.27 (95% CI: 0.04–1.91, I^2^ = 61%, *n* = 2). The results were similar for tree nuts and peanuts. If the associations are causal, an estimated 4.4 million premature deaths in the America, Europe, Southeast Asia, and Western Pacific would be attributable to a nut intake below 20 grams per day in 2013.

**Conclusions:**

Higher nut intake is associated with reduced risk of cardiovascular disease, total cancer and all-cause mortality, and mortality from respiratory disease, diabetes, and infections.

**Electronic supplementary material:**

The online version of this article (doi:10.1186/s12916-016-0730-3) contains supplementary material, which is available to authorized users.

## Background

Cardiovascular disease and cancer remain the two most common causes of death, accounting for 25.5 million deaths worldwide in 2013 [1]. Epidemiological and intervention studies have shown that a high intake of nuts is associated with a reduced risk of coronary heart disease and possibly other health outcomes such as diabetes, overweight and obesity, gallstones, and colorectal cancer [2–4]. Tree nuts, botanically defined as dry fruit containing one seed (rarely two) within the ovary wall that becomes hard at maturity, include walnuts, almonds, hazelnuts, cashews, pistachios, and pecans [5]. While Brazil nuts and peanuts are botanically classified as seeds and legumes, respectively, all of tree nuts, Brazil nuts, and peanuts are collectively referred to as nuts due to their similar nutritional properties and culinary use. Nuts are good sources of dietary fiber, magnesium, polyunsaturated fats, vitamin E, and antioxidants, all of which may reduce risk of cardiovascular disease by reducing insulin resistance [6], cholesterol concentrations [4], lipid peroxidation [7], and oxidative stress [8]. Nuts also contain other bioactive compounds, such as ellagic acid, anacardic acid, genistein, resveratrol, and inositol phosphates, which may reduce cancer risk by inducing cell cycle arrest, apoptosis, inhibiting cell proliferation, migration, invasion, and angiogenesis [9]. However, epidemiological data on nuts and cancer risk are less extensive than for cardiovascular disease.

There is a growing body of evidence suggesting a role of nut consumption in reducing risk of coronary heart disease [2, 10–17] and mortality [10, 12–16, 18–22]. However, whether a high intake of nuts is associated with risk of stroke [13–15, 23–27] or overall cancer risk [13–15, 21, 24, 28] is not clear, as most studies reported no significant association [13–15, 21, 23, 25–27] and only a few reported significant inverse associations [14, 16, 21]. Nevertheless, the possibility that a weak association may have been missed because of low statistical power cannot be excluded. Although a few previous reviews reported a reduced risk of coronary heart disease and mortality [29–31] with higher nut intake, associations with stroke have been unclear, with one meta-analysis finding no statistically significant association [32], but another meta-analysis reporting a significant inverse association [33]. However, in the latter, the Nurses’ Health Study and the Health Professionals Follow-up Study had been included twice, thus, questions remain with regards to whether there is an association between nut intake and stroke. In addition, several large cohort studies including 47,061 deaths and > 748,000 additional participants were either not included [20, 28] or have been published [14, 15, 22, 34–37] since these reviews, and more detailed and updated analyses have since been published from the Physicians’ Health Study [15] and the Netherlands Cohort Study [16]. Associations between nut consumption and less common causes of death have not been systematically assessed. Therefore, we conducted a systematic review and meta-analysis of prospective studies of nut consumption and the risk of coronary heart disease, stroke, cardiovascular disease, total cancer, and all-cause mortality as well as less common causes of death to provide a more up-to-date and comprehensive assessment of the available evidence. We aimed to clarify the strength and shape of the dose–response relationship between nut consumption and these outcomes, identify potential differences by type of nuts consumed (total nuts, tree nuts, peanuts), as well as potential sources of heterogeneity between studies by geographic location. To examine the health impact of low nut consumption we also estimated the number of deaths in North and South America, Europe, Southeast Asia, and the Western Pacific attributable to low nut consumption based on regional studies [14, 38–44] and data on mortality from the Global Burden of Disease Study [1].

## Methods

### Search strategy and inclusion criteria

**Fig. 1:**
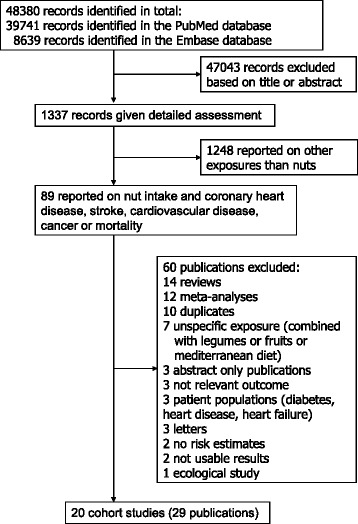
Flow-chart of study selection

The PubMed and EMBASE databases were searched from their inception (1966 and 1947, respectively) to July 19, 2016. The search terms used for the PubMed search are provided in Additional file 1: Table S1 and a similar search was conducted in EMBASE. Published prospective studies (cohort studies, case-cohort studies, nested case–control studies within cohort studies, and randomized trials) of nut intake (any type of edible nut consumption, including all dosages) among mainly adult populations and incidence or mortality from coronary heart disease, stroke, cardiovascular disease, total cancer, and all-cause (primary outcomes) and cause-specific mortality (secondary outcomes) from any cause of death investigated by at least two studies were included if they reported adjusted relative risk (RR) estimates and 95% confidence intervals (CIs). For the dose–response analyses, a quantitative measure of the intake for at least three categories of nut intake or a risk estimate on a continuous scale had to be available. Retrospective case–control studies were excluded because of the greater potential for recall and selection bias, while cross-sectional studies were excluded because of the difficulty of drawing conclusions with regard to the cause and effect. Reviews, meta-analyses, duplicate publications, studies with an unspecific exposure (e.g., nut intake was combined with fruits or legumes), studies on other outcomes, studies that did not report adjusted risk estimates, ecological studies, letters, and studies with unusable data, as well as abstracts, grey literature, and unpublished studies, were not included. When duplicate publications were published from the same studies we chose the publication with the largest number of cases or deaths for inclusion. We searched the references of the retrieved reports for any additional studies. The first author of one study [20] was contacted to obtain information with regard to the amount of nut intake for each category of intake and this information was provided. DA conducted the literature search and the screening of the studies and TN screened in duplicate the 89 potentially relevant studies identified from the initial screening (Fig. 1). Any discrepancies were resolved by discussion. Study quality was assessed by two authors (DA, DCG) using the Newcastle–Ottawa Scale, which awards a score of 0–9 based on the selection, comparability, and outcome assessment [45]. We considered studies with a score of 0–3, 4–6, and 7–9 to represent low, medium, and high quality studies, respectively. We followed the PRISMA criteria for reporting of meta-analyses of observational studies [46]. A list of the excluded studies is provided in Additional file 1: Table S2. Although there was no protocol for the current review, we followed standard methods and analytic approaches similar to our previous meta-analysis [47].

### Data extraction

The following data from the studies were extracted into tables: name of first author, publication year, country or region, the name of the study, follow-up period, sample size and number of cases or deaths, type of outcome, gender, age, type of nuts (total nuts, tree nuts, walnuts, peanuts, peanut butter), amount or frequency of intake, RRs and 95% CIs, and variables adjusted for in the analysis. DA conducted the data extraction which was checked for accuracy by NK.

### Statistical methods

Summary RRs and 95% CIs of cardiovascular disease, total cancer, and all-cause and cause-specific mortality for the highest versus the lowest level and per one serving per day increase in nut intake were calculated using the random effects model [48], which takes into account both within and between study variation (heterogeneity). The RR from each study was weighted by the method of DerSimonian and Laird [48] and the average of the natural logarithm of the RRs was estimated. When data were reported separately by sex or other subgroups we pooled the RRs using a fixed effects model before inclusion in the overall meta-analysis. A two-tailed *P* < 0.05 was considered statistically significant.

Linear dose–response analyses were conducted using the method by Greenland and Longnecker [49] and we computed study-specific slopes (linear trends) and 95% CIs from the natural logarithm of the RRs across categories of nut intake. A serving size of nuts was defined as one ounce or 28 grams [12, 13, 21], and serving sizes were converted accordingly unless authors specified another serving size. The mean or median level of nut intake was used if reported in the paper, and for studies that reported nut intake by ranges of intake we estimated the midpoint of the upper and lower bound. When extreme categories were open-ended or had extreme upper or lower values, we used the width of the adjacent interval to calculate an upper or lower cut-off value. Potential nonlinear dose–response relationships between nut intake and cardiovascular disease, cancer, and mortality were assessed using restricted cubic splines, with three knots at 10%, 50%, and 90% percentiles of the distribution, which were combined using multivariate meta-analysis [50, 51]. We tested for nonlinearity by using a likelihood ratio test to assess the difference between the nonlinear and linear models [52].

Heterogeneity between studies was assessed using Q and I^2^ statistics [53]. A *P* < 0.10 was considered to be statistically significant for the Q statistic. I^2^ is the proportion of total variation that is explained by between-study variation. Sources of heterogeneity were investigated in subgroup analyses stratified by sex, duration of follow-up, geographic location, number of cases/deaths, study quality score, and adjustment for confounding factors (age, education, family history of cardiovascular disease, body mass index (BMI), smoking, alcohol, physical activity, hypertension, hypercholesterolemia/serum cholesterol, coffee/caffeine, sugar-sweetened beverages, red and/or processed meat, fish, fruit and vegetables, whole grains, dairy products, and energy intake). Small study effects, such as publication bias, were assessed using Egger’s test [54] and by inspection of funnel plots. Stata version 12.0 software (StataCorp, Texas, US) was used for the analyses.

### Population-attributable risk

In a secondary analysis, we estimated the number of deaths from all causes and specific causes that could potentially be avoided, assuming a causal relationship between nut intake and mortality in Europe, North and South America, and Southeast Asia and Western Pacific, using data on nut intake from cohort studies and dietary surveys in these regions [14, 38–44], data on mortality from the Global Burden of Disease Study 2013 [1], and the summary RRs from the nonlinear dose–response meta-analysis of mortality from coronary heart disease, total cancer, all causes, respiratory disease, and diabetes. The formula by Miettinen [55] was used to calculate the population-attributable risk of mortality due to low nut consumption: PAR = p(rr–1)/(1 + p(rr–1)), where p is the prevalence of the exposure in the population, and rr is the relative risk. Because there was evidence of nonlinearity between nut consumption and mortality and most specific causes of death we used the relative risk estimates derived from the nonlinear analyses. We used 20 grams per day as the optimal intake, because there was little evidence of further reductions in risk above this level of intake in the current meta-analysis. The prevalence of nut intake was calculated in increments of 10 grams per day (0, > 0 to < 10, 10 to < 20, compared to ≥ 20 (reference)), and we used the relative risk at zero intake and at the midpoint of each category (0, 5, 15, compared to ≥ 20). Because all the epidemiological studies included in this meta-analysis have been conducted in mainly adult populations we excluded the number of deaths occurring before 15 years of age.

## Results

**Fig. 2:**
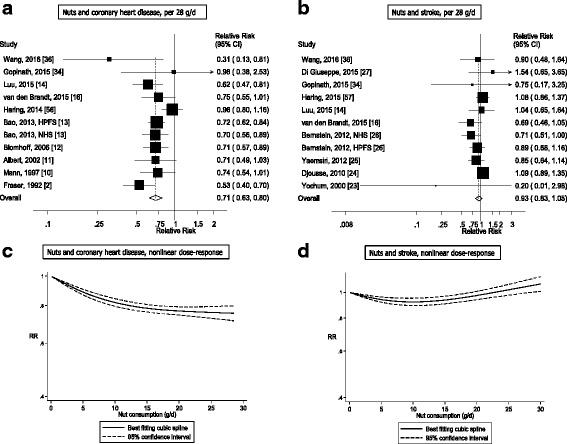
Nut consumption and coronary heart disease and stroke

**Fig. 3:**
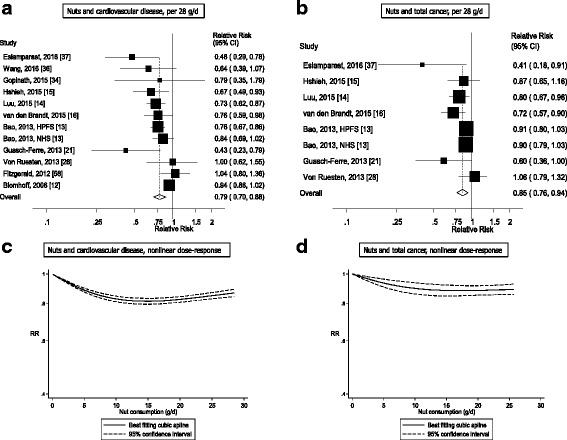
Nut consumption and cardiovascular disease and total cancer

**Fig. 4:**
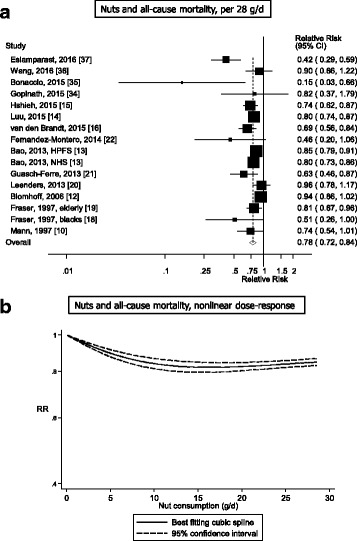
Nut consumption and all-cause mortality

**Fig. 5:**
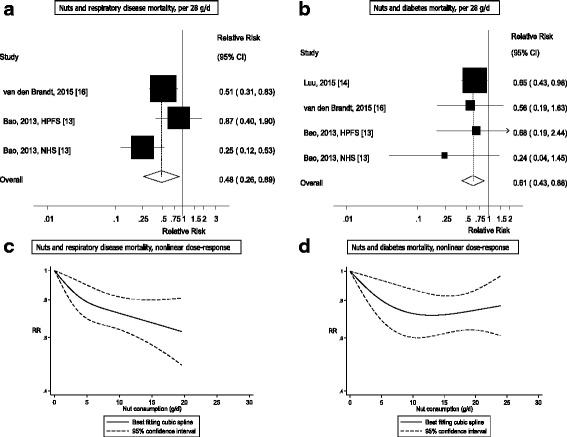
Nut consumption respiratory disease and diabetes mortality

**Fig. 6:**
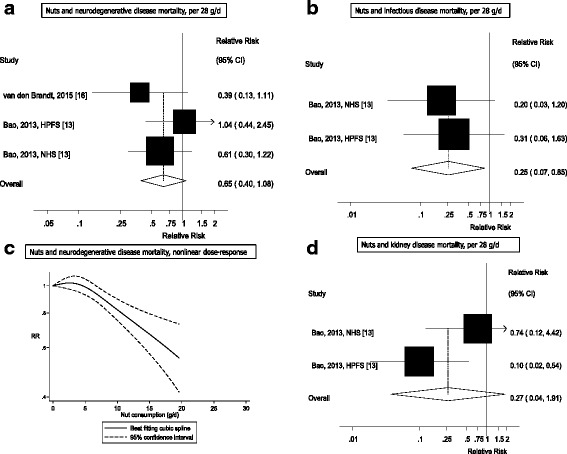
Nut consumption neurodegenerative disease, infectious disease, and kidney disease mortality

Out of a total of 48,380 records identified by the search, 1337 were given detailed assessment, and 89 of these reporting on nut intake were considered potentially eligible for inclusion. Twenty prospective cohort studies (29 publications) [2, 10–28, 34–37, 56–60] (some studies had more than one publication, but reported on different outcomes in each included publication) were included in the analysis of nut intake and coronary heart disease, stroke, cardiovascular disease, total cancer, all-cause mortality, and other causes of mortality (Additional file 1: Table S3–S13). For inflammatory disease mortality, there was only one study [60] so it was not possible to conduct a meta-analysis, but the results are reviewed in Additional file 1: Table S13. No additional studies were identified by scanning the reference lists of the included studies and previous reviews. The dose-response analyses of nut intake included 12,331 coronary heart disease cases, 9272 stroke cases, 18,655 cardiovascular disease cases, 18,490 cancer cases, and 85,870 deaths among up to 819,448 participants. Nine studies were from the US, six from Europe, four from Asia, and one from Australia. All studies were among mainly adult populations, although one study had an age range of 16–79 years [10]. Three studies were among men only, five among women only, and 12 in both sexes. A summary of the study characteristics of the included studies is provided in Additional file 1: Table S3–S13. Figure 1 shows a flow-chart of the study selection. Figures 2, 3, 4, 5 and 6 shows the results of the dose–response analyses and Additional file 1: Figure S1–S10 shows the results from the high versus low analyses. Results for specific tree nuts, peanuts, and peanut butter are provided in Table 1 and Additional file 1: Figure S11–S69.

**Table 1:** Nut consumption and coronary heart disease, stroke, cardiovascular disease, total cancer, mortality, and other causes of mortality

Outcome^a^	Exposure	Cases or deaths	Participants	High vs. low analysis				Dose–response analysis^b^			
				N	RR (95% CI)	I^2^ (%)	*P*_heterogeneity_	N	RR (95% CI)	I^2^ (%)	*P*_heterogeneity_
Coronary heart disease	Total nuts	12,331	315,397	11	**0.76 (0.69–0.84)**	47.5	0.04	11	**0.71 (0.63–0.80)**	47.4	0.04
	Tree nuts	6394	130,987	3	**0.79 (0.68–0.92)**	28.0	0.25	3	**0.73 (0.63–0.85)**	0	0.44
	Peanuts	7025	265,252	5	**0.76 (0.69–0.82)**	0	0.65	5	**0.69 (0.57–0.84)**	45.1	0.12
Stroke	Total nuts	9272	396,768	10	**0.89 (0.82–0.97)**	0	0.90	11	0.93 (0.83–1.05)	13.7	0.31
	Tree nuts	2130	130,987	3	0.93 (0.77–1.13)	0	0.44	3	0.89 (0.69–1.14)	0	0.58
	Peanuts	3315	265,252	5	0.83 (0.69–1.00)	45.9	0.12	5	**0.63 (0.41–0.95)**	77.6	0.001
Cardiovascular disease	Total nuts	18,655	376,228	11	**0.81 (0.74–0.89)**	52.3	0.02	12	**0.79 (0.70–0.88)**	59.6	0.004
	Tree nuts	9456	130,987	3	**0.81 (0.74–0.89)**	0	0.62	3	**0.75 (0.67–0.84)**	0	0.84
	Peanuts	12,043	265,252	5	**0.81 (0.75–0.87)**	15.0	0.32	5	**0.64 (0.50–0.81)**	77.0	0.001
Total cancer	Total nuts	17,603	254,240	8	**0.82 (0.74–0.89)**	27.5	0.21	8	**0.85 (0.76–0.94)**	41.8	0.10
	Tree nuts	14,210	130,987	3	**0.82 (0.76–0.90)**	0	0.98	3	**0.80 (0.72–0.89)**	0	0.99
	Peanuts	17,742	265,252	5	**0.93 (0.87–0.99)**	19	0.30	5	0.92 (0.82–1.03)	30.0	0.22
All-cause mortality	Total nuts	85,870	819,448	15	**0.81 (0.77–0.85)**	41.0	0.05	16	**0.78 (0.72–0.84)**	66.0	<,0.0001
	Tree nuts	42,508	202,751	4	**0.80 (0.74–0.86)**	58.0	0.07	4	**0.82 (0.75–0.90)**	70.0	0.02
	Peanuts	44,396	265,252	5	**0.85 (0.82–0.89)**	18.0	0.30	5	**0.77 (0.69–0.86)**	64.0	0.03
	Peanut butter	15,079	83,789	2	**0.89 (0.80–0.99)**	31.0	0.23	2	0.94 (0.86–1.02)	0	0.76
Respiratory disease mortality	Total nuts	2551	130,987	3	**0.76 (0.61–0.94)**	53.9	0.09	3	**0.48 (0.26–0.89)**	60.8	0.08
	Tree nuts	2551	130,987	3	0.89 (0.74–1.07)	0	0.78	3	0.79 (0.62–1.01)	0	0.43
	Peanuts	2551	130,987	3	**0.77 (0.63–0.93)**	39.2	0.19	3	**0.69 (0.53–0.91)**	49.8	0.14
Diabetes mortality	Total nuts	800	202,751	4	**0.68 (0.52–0.90)**	0	0.59	4	**0.61 (0.43–0.88)**	0	0.76
	Tree nuts	462	130,987	3	1.19 (0.74–1.89)	0	0.43	3	1.23 (0.68–2.25)	0	0.62
	Peanuts	901	265,252	5	0.84 (0.60–1.19)	42.6	0.14	5	0.73 (0.45–1.20)	15.4	0.32
Neurodegenerative disease mortality	Total nuts	2056	130,987	3	0.93 (0.72–1.21)	6	0.30	3	0.65 (0.40–1.08)	5.9	0.35
	Tree nuts	2056	130,987	3	0.94 (0.75–1.18)	13.8	0.31	3	0.81 (0.58–1.12)	25.6	0.26
	Peanuts	2056	130,987	3	0.94 (0.72–1.23)	45.9	0.16	3	0.92 (0.65–1.31)	48.6	0.14
Infectious disease mortality	Total nuts	397	118,962	2	0.79 (0.56–1.11)	0	0.49	2	**0.25 (0.07–0.85)**	0	0.74
	Tree nuts	397	118,962	2	0.73 (0.47–1.13)	0	0.79	2	0.64 (0.36–1.13)	0	0.40
	Peanuts	397	118,962	2	1.01 (0.83–1.23)	12.5	0.29	2	1.03 (0.81–1.31)	2.6	0.31
Kidney disease mortality	Total nuts	367	118,962	2	0.69 (0.38–1.25)	69.0	0.07	2	0.27 (0.04–1.91)	61.0	0.11
	Tree nuts	367	118,962	2	0.65 (0.40–1.03)	0	0.48	2	0.66 (0.36–1.22)	0	0.68
	Peanuts	367	118,962	2	**0.52 (0.27–0.97)**	52.1	0.15	2	**0.42 (0.24–0.73)**	0	0.35

^a^Associations which were statistically significant are shown with bold font

^b^The increment for the dose-response analysis is 28 g/d for total nuts and 10 g/d for tree nuts and peanuts

### Nuts and coronary heart disease

Twelve cohort studies (11 publications) [2, 10–14, 16, 34–36, 56] investigated the association between nut intake and coronary heart disease risk and the dose-response analysis included a total of 12,331 cases and 315,397 participants. One publication was only included in the subgroup analysis of coronary heart disease incidence [17], while another publication was only included in the subgroup analysis of coronary heart disease mortality [15]. The summary RR for high versus low intake was 0.76 (95% CI: 0.69–0.84, I^2^ = 42%, *P*_heterogeneity_ = 0.06) (Additional file 1: Figure S1). The summary RR for a one serving per day increment was 0.71 (95% CI: 0.63–0.80, I^2^ = 47%, *P*_heterogeneity_ = 0.04) (Fig. 2a, Table 1). There was no evidence of small study bias with Egger’s test, *P* = 0.28. There was evidence of a nonlinear association between nut intake and coronary heart disease, *P*_nonlinearity_ < 0.0001, with only slight further reductions in risk above 15–20 grams per day (Fig. 2b, Additional file 1: Table S14).

The summary RR for high versus low intake of tree nuts was 0.79 (95% CI: 0.68–0.92, I^2^ = 28%, *P*_heterogeneity_ = 0.25, *n* = 3) [13, 16] and peanuts was 0.76 (95% CI: 0.69–0.82, I^2^ = 0%, *P*_heterogeneity_ = 0.65, *n* = 5) [13, 14, 16] and the respective summary RRs per 10 grams/day increase in intake were 0.73 (95% CI: 0.63–0.85, I^2^ = 0%, *P*_heterogeneity_ = 0.44, *n* = 3) and 0.69 (95% CI: 0.57–0.84, I^2^ = 45%, *P*_heterogeneity_ = 0.12, *n* = 5) (Additional file 1: Figure S11–S16, Table 1).

### Nuts and stroke

Eleven cohort studies (10 publications) [14–16, 23, 25–27, 34–36, 57] were included in the analysis of nut intake and risk of stroke and the dose-response analysis included a total of 9272 cases and 396,768 participants. The summary RR for high versus low intake was 0.89 (95% CI: 0.82–0.97, I^2^ = 0%, *P*_heterogeneity_ = 0.90) (Additional file 1: Figure S2). The summary RR per one serving per day was 0.93 (95% CI: 0.83–1.05, I^2^ = 14%, *P*_heterogeneity_ = 0.31) (Fig. 2c, Table 1). There was no evidence of small study bias with Egger’s test (*P* = 0.30). There was evidence of a nonlinear association between nut intake and stroke risk, *P*_nonlinearity_ < 0.0001, with a slight J-shaped curve with reductions in risk observed up to approximately 10–15 grams per day, but a slight positive association at intakes of 30 grams per day, however, this was not observed when studies were stratified by whether the outcome was stroke incidence or stroke mortality (Fig. 2d, Additional file 1: Table S14).

The summary RR for high versus low intake of tree nuts was 0.93 (95% CI: 0.77–1.13, I^2^ = 0%, *P*_heterogeneity_ = 0.44, *n* = 3) [13, 16] and of peanuts was 0.83 (95% CI: 0.69–1.00, I^2^ = 46%, *P*_heterogeneity_ = 0.12, *n* = 5) [13, 14, 16] and the respective summary RRs per 10 grams/day increase in intake were 0.89 (95% CI: 0.69–1.14, I^2^ = 0%, *P*_heterogeneity_ = 0.58, *n* = 3) and 0.63 (95% CI: 0.41–0.95, I^2^ = 78%, *P*_heterogeneity_ = 0.001, *n* = 5) (Additional file 1: Figure S17–S22, Table 1).

### Nuts and cardiovascular disease

Twelve cohort studies (11 publications) [12–16, 21, 28, 34–37, 58] investigated nut intake and cardiovascular disease risk and the dose-response analysis included a total of 18,655 cases and 376,228 participants. One publication was included in the subgroup analysis of cardiovascular disease incidence only [59]. The summary RR for high versus low intake was 0.81 (95% CI: 0.74–0.89, I^2^ = 52%, *P*_heterogeneity_ = 0.02) (Additional file 1: Figure S3). The summary RR was 0.79 (95% CI: 0.70–0.88, I^2^ = 60%, *P*_heterogeneity_ = 0.004) per one serving per day (Fig. 3a). There was some suggestion of small study bias with Egger’s test (*P* = 0.07), but this was explained by one small study [21] and, when excluded, Egger’s test showed *P* = 0.16, and the summary estimate was not materially altered (summary RR = 0.80; 95% CI: 0.72–0.89, I^2^ = 56%, *P*_heterogeneity_ = 0.01). There was evidence of a nonlinear association between nut intake and cardiovascular disease risk (*P*_nonlinearity_ = 0.001), with a reduction in risk observed up to an intake of approximately 15 g/d, but no further reductions with higher intakes (Fig. 3b, Additional file 1: Table S14).

The summary RR for high versus low intake of tree nuts was 0.76 (95% CI: 0.69–0.84, I^2^ = 0%, *P*_heterogeneity_ = 0.92, *n* = 3) [13, 16] and peanuts was 0.76 (95% CI: 0.70–0.81, I^2^ = 0%, *P*_heterogeneity_ = 0.90, *n* = 5) [13, 14, 16] and the respective summary RRs per 10 grams/day increase in intake were 0.75 (95% CI: 0.67–0.84, I^2^ = 0%, *P*_heterogeneity_ = 0.84, *n* = 3) and 0.64 (95% CI: 0.50–0.81, I^2^ = 77%, *P*_heterogeneity_ = 0.001, *n* = 5) (Additional file 1: Figure S23–S28, Table 1).

### Nuts and total cancer

Nine cohort studies (8 publications) [13–16, 21, 28, 35, 37] were included in the analysis of nut intake and total cancer risk and the dose-response analysis included 18,490 cancer cases among 304,285 participants. The summary RR for the high versus low intake was 0.82 (95% CI: 0.74–0.89, I^2^ = 28%, *P*_heterogeneity_ = 0.21) (Additional file 1: Figure S4). The summary RR per one serving per day was 0.85 (95% CI: 0.76–0.94, I^2^ = 42%, *P*_heterogeneity_ = 0.10) (Fig. 3c, Table 1). Egger’s test for small study bias was not significant (*P* = 0.10). There was no evidence of a nonlinear association between nut intake and total cancer (*P*_nonlinearity_ = 0.11) (Fig. 3d, Additional file 1: Table S15).

The summary RR for high versus low intake of tree nuts was 0.82 (95% CI: 0.76–0.90, I^2^ = 0%, *P*_heterogeneity_ = 0.98, *n* = 3) [13, 16] and peanuts was 0.93 (95% CI: 0.87–0.99, I^2^ = 19%, *P*_heterogeneity_ = 0.30, *n* = 5) [13, 14, 16], and the respective summary RRs per 10 grams/day increase in intake were 0.80 (95% CI: 0.72–0.89, I^2^ = 0%, *P*_heterogeneity_ = 0.99, *n* = 3) and 0.92 (95% CI: 0.82–1.03, I^2^ = 30%, *P*_heterogeneity_ = 0.22, *n* = 5) (Additional file 1: Figure S29–S34, Table 1).

### Nuts and all-cause mortality

Fifteen cohort studies (15 publications, 16 risk estimates) [10, 12–16, 18–22, 34–37] were included in the analysis of nut intake and all-cause mortality and the dose-response analysis included 85,870 deaths and 819,448 participants. The summary RR for high versus low intake was 0.81 (95% CI: 0.77–0.85, I^2^ = 41%, *P*_heterogeneity_ = 0.05) (Additional file 1: Figure S5). The summary RR was 0.78 (95% CI: 0.72–0.84, I^2^ = 66%, *P*_heterogeneity_ < 0.0001) per one serving per day (Fig. 4a, Table 1). There was suggestion of small study bias with Egger’s test (*P* = 0.02) (Additional file 1: Figure S35); however, after excluding five studies with less than 500 deaths [10, 18, 21, 22, 35], Egger’s test was no longer significant (*P* = 0.25), and there was no asymmetry in the funnel plot, while association remained similar (summary RR = 0.80; 95% CI: 0.74–0.87). There was evidence of a nonlinear association between nut consumption and all-cause mortality (*P*_nonlinearity_ < 0.0001), with a steeper reduction in risk at lower intakes, and no further reduction in risk above 15–20 grams per day (Fig. 4b, Additional file 1: Table S15).

The summary RR for high versus low intake of tree nuts was 0.80 (95% CI: 0.74–0.86, I^2^ = 58%, *P*_heterogeneity_ = 0.07, *n* = 4) [13, 14, 16], that of peanuts was 0.85 (95% CI: 0.81–0.89, I^2^ = 34%, *P*_heterogeneity_ = 0.19, *n* = 5) [13, 14, 16], and that of peanut butter was 0.89 (95% CI: 0.80–0.99, I^2^ = 31%, *P*_heterogeneity_ = 0.23, *n* = 2) [14, 16], and the respective summary RRs per 10 grams/day increase in intake were 0.82 (95% CI: 0.75–0.90, I^2^ = 70%, *P*_heterogeneity_ = 0.02, *n* = 3), 0.77 (95% CI: 0.69–0.86, I^2^ = 64%, *P*_heterogeneity_ = 0.03, *n* = 5), and 0.94 (95% CI: 0.86–1.02, I^2^ = 0%, *P*_heterogeneity_ = 0.76, *n* = 2), respectively (Additional file 1: Figure S36–S43, Table 1).

### Nuts and other causes of mortality

The summary RR for high versus low intake was 0.76 (95% CI: 0.61–0.94, I^2^ = 54%, *P*_heterogeneity_ = 0.11, *n* = 3 studies, 2551 deaths, 122,164 participants) for respiratory disease mortality (Additional file 1: Figure S6), 0.68 (95% CI: 0.52–0.90, I^2^ = 0%, *P*_heterogeneity_ = 0.59, *n* = 4, 800 deaths, 193,928 participants) for diabetes mortality (Additional file 1: Figure S7), 0.93 (95% CI: 0.72–1.21, I^2^ = 53%, *P*_heterogeneity_ = 0.12, *n* = 3, 2056 deaths, 122,164 participants) for neurodegenerative disease mortality (Additional file 1: Figure S8), 0.79 (95% CI: 0.56–1.11, I^2^ = 0%, *P*_heterogeneity_ = 0.49, *n* = 2397 deaths, 118,962 participants) for infectious disease mortality (Additional file 1: Figure S9), and 0.69 (95% CI: 0.38–1.25, I^2^ = 69%, *P*_heterogeneity_ = 0.07, *n* = 2367 deaths, 118,962 participants) for kidney disease mortality (Additional file 1: Figure S10). The respective summary RRs per one serving per day increase in nut intake was 0.48 (95% CI: 0.26–0.89, I^2^ = 61%, *P*_heterogeneity_ = 0.08, *n* = 3), 0.61 (95% CI: 0.43–0.88, I^2^ = 0%, *P*_heterogeneity_ = 0.76, *n* = 4), 0.65 (95% CI: 0.40–1.08, I^2^ = 5.9%, *P*_heterogeneity_ = 0.35, *n* = 3), 0.25 (95% CI: 0.07–0.85, I^2^ = 0%, *P*_heterogeneity_ = 0.74, *n* = 2), and 0.27 (95% CI: 0.04–1.91, I^2^ = 61%, *P*_heterogeneity_ = 0.11, *n* = 2) (Figs. 5 and 6, Additional file 1: Table S15). Results for subtypes of nuts in relation to other causes of mortality were in general similar to the overall results (Table 1, Additional file 1: Figure S44–S69). Only one study reported on nut intake and inflammatory disease mortality and reported a reduced risk with higher intake of nuts; however, it was not possible to conduct a meta-analysis for this outcome (Additional file 1: Table S13).

### Subgroup and meta-regression analyses, study quality, and sensitivity analyses

In subgroup and meta-regression analyses there was little evidence of heterogeneity between subgroups when analyses were stratified by study characteristics including duration of follow-up, sex, geographic location, number of cases or deaths, study quality scores, and adjustment for potential confounding factors (Additional file 1: Table S16 and S17). There was indication of a stronger association between nut intake and cardiovascular disease among men than among women (*P*_heterogeneity_ = 0.02) (Additional file 1: Table S16); however, there was no evidence of a difference of the other associations by sex. There was also heterogeneity in the analysis of nuts and cardiovascular disease when stratified by adjustment for smoking (*P*_heterogeneity_ = 0.02), hypertension (*P*_heterogeneity_ = 0.007), and whole grains (*P*_heterogeneity_ = 0.01), with stronger associations among studies with adjustment for smoking and hypertension than among studies without such adjustment, and a weaker association among studies with adjustment for whole grains than without such adjustment. For all-cause mortality there was evidence of a weaker association among studies with a longer duration of follow-up compared to studies with a shorter duration of follow-up (*P*_heterogeneity_ = 0.03).

Mean (median) study quality scores were 7.4 (8.0) for coronary heart disease, 7.7 (8.0) for stroke, 7.6 (8.0) for cardiovascular disease, 8.0 (8.0) for total cancer, and 7.3 (7.5) for all-cause mortality (Additional file 1: Table S18–S22). With regard to the study quality score some parts of the score more often gave zero points than others including representativeness of the cohort, exposure ascertainment (lack of interview or lack of validated FFQ), demonstration that the outcome was not present at the beginning of the study (no exclusion of prevalent cases), and adequacy of follow-up (loss to follow-up of more than 10% or not stated), while the parts of the score that covered the selection of the non-exposed cohort, adjustment for confounding factors, assessment of the outcome, and having a long enough follow-up for cases to accrue, appeared to be met across studies.

In sensitivity analyses excluding one study at a time from the analysis the results were robust to the influence of individual studies in the analysis of coronary heart disease, stroke, cardiovascular disease, total cancer, and mortality (Additional file 1: Figure S70–S74).

### Population-attributable risk

Under the assumption that the associations observed between nut consumption and mortality are causal, we estimated that a total of 4.4 million deaths may be attributable to a nut intake of less than 20 grams per day in 2013 in the regions covered (Additional file 1: Table S23). Of specific causes of death we estimated 1.19 million deaths due to coronary heart disease, 469,000 deaths due to cancer, 1.07 million deaths due to respiratory disease, and 139,000 deaths due to diabetes may be attributable to a nut intake below 20 grams per day in 2013 in the same regions (Additional file 1: Table S23).

## Discussion

In this meta-analysis there was a 24%, 11%, 19%, 18%, and 19% reduction in the relative risk of coronary heart disease, stroke, cardiovascular disease, total cancer, and all-cause mortality with a higher nut intake, respectively. In the dose–response analysis there was a 29%, 7%, 21%, 15%, and 22% reduction in the relative risk for a one serving per day increase in nut intake (one serving = 28 grams), respectively, although the association for stroke was not statistically significant in the linear dose–response analysis. There was evidence of a nonlinear association between nut intake and coronary heart disease, stroke, cardiovascular disease, total cancer, and all-cause mortality, with most of the reduction in risk observed up to an intake of approximately 15–20 grams per day or 5–6 servings per week for most of the outcomes. In addition, there was a 52%, 39%, and 75% reduction in the relative risk of respiratory disease, diabetes, and infectious disease mortality, respectively, for a one serving per day increase in intake, and non-significant inverse associations were also observed for neurodegenerative disease mortality and kidney disease mortality, although the number of studies was low. The intake of both peanuts and tree nuts was associated with a reduced risk of coronary heart disease, cardiovascular disease, and mortality; however, only the intake of peanuts was associated with reduced risk of stroke, while the intake of tree nuts was associated with reduced cancer risk. Inverse associations were observed in European and American studies as well as in Asian studies of peanuts. Intake of peanut butter was inversely associated with mortality in the high versus low analysis, but not in the dose–response analysis. Although it is possible that the added sugar or salt in peanut butter could counteract any beneficial effects of plain peanuts, the limited number of studies makes the interpretation of those results difficult.

Under the assumption that the observed associations are causal we estimated that approximately 4.4 million premature deaths in the regions covered, including North and South America, Europe, Southeast Asia, and Western Pacific, may be attributable to a nut intake below 20 grams per day. For specific causes of death, we estimated that 1.19 million deaths due to coronary heart disease, 469,000 due to cancer, 1.07 million due to respiratory disease, and 138,000 due to diabetes may be caused by a nut intake below 20 grams per day. However, the appropriateness of these estimates is dependent on the validity of several assumptions, including that of (1) a causal relationship between nut consumption and these outcomes, (2) lack of confounding and measurement error in explaining the results, and (3) the generalizability of the results to the regions covered.

Our meta-analysis provides the most up-to-date summary estimates of the association between nut consumption and cardiovascular disease, cancer, and all-cause and cause-specific mortality and is consistent with previous reviews and meta-analyses that have been published on the topic [29, 31, 61]. Nevertheless, the current meta-analysis contains 1.5–3 times the number of studies compared to the previously published meta-analyses and approximately twice the number of all-cause deaths. For example, in the dose–response analysis of nut intake and all-cause mortality we included 15 studies (85,870 deaths), compared to 5 studies (48,818 deaths) [29], 7 studies (44,636 deaths) [31], and 10 studies (49,232 deaths) in previous meta-analyses [61]. In the dose–response analysis of coronary heart disease, stroke, cardiovascular disease, and total cancer, we included 11, 11, 12, and 8 studies, respectively, compared to 6 and 4 studies for coronary heart disease and stroke in one meta-analysis [30], 7 and 4 studies on coronary heart disease and stroke in another meta-analysis [62], 5 and 3 studies for cardiovascular and cancer mortality in a third meta-analysis [31], and 4 studies on cancer mortality in a fourth meta-analysis [16]. In addition, we found inverse associations between nut consumption and mortality from respiratory disease, diabetes, and infections, although the number of studies in these analyses was low and further studies are needed to confirm these findings.

Some potential limitations of our meta-analysis should be mentioned. There was high heterogeneity in the analysis of cardiovascular disease and all-cause mortality. Some heterogeneity is expected because of differences in the age groups included, geographic location, detail of the dietary assessment, factors adjusted for in the analyses, types of nuts consumed, as well as the distribution of specific causes of death and cancers that contribute to all-cause mortality and total cancer. For cardiovascular disease and all-cause mortality, the heterogeneity was driven more by differences in the strength of the associations than differences in the direction of the association. In the analysis of cardiovascular disease, there was between-subgroup heterogeneity when stratified by sex, with a stronger inverse association among men than women and no heterogeneity within subgroups. However, associations for coronary heart disease and stroke were similar when stratified by sex, thus we cannot exclude the possibility that chance could explain this sex difference for cardiovascular disease. There was also heterogeneity when stratified by adjustment for smoking, hypertension, and whole grains, with stronger associations in studies with adjustment for smoking and hypertension than in studies without such adjustment and a weaker association among studies with adjustment for whole grains than in studies without. In the analysis of all-cause mortality there was little evidence of heterogeneity between subgroups, with the exception of a slightly weaker association among studies with a longer compared to a shorter duration of follow-up. There was no evidence of heterogeneity in the analysis of stroke and total cancer, and moderate heterogeneity in the analysis of coronary heart disease. There was no evidence of heterogeneity when analyses were stratified by geographic location. Although there were some differences among the included studies with regard to the amounts and ranges of nut intake, this was taken into account in the dose–response analyses. Subjects with a high intake of nuts tend to be less likely to smoke, to be slimmer and more physically active, and to have a lower intake of red and processed meat and a higher intake of fruits and vegetables than persons with a low nut intake [13], thus confounding by other lifestyle and dietary factors is a potential source of bias. However, in subgroup analyses we found that the associations observed persisted among studies which adjusted for smoking, alcohol, physical activity, BMI, and dietary factors such as red meat and fruit and vegetables. In addition, in the Nurses’ Health Study and the Health Professionals Follow-up Study the inverse associations persisted when analyses were stratified by these and other potential confounding factors [13]. Measurement errors may have affected the findings, but none of the included studies made corrections for measurement error; however, because of the prospective design of the included studies such errors would most likely attenuate the strength of the observed associations [63].

There was a limited number of studies in the analyses of mortality from respiratory disease, diabetes, infections, neurodegenerative disease, and kidney disease and the potential for selective reporting cannot be entirely excluded. However, it is also possible that most of the included studies may have been too small to have adequate power to investigate these less common causes of death. Further studies are therefore needed on these and other less common causes of death. We did not identify any potentially relevant studies in non-English language, thus language bias is less likely to have influenced the findings. None of the included studies were identified solely from the screening of article references, thus it seems less likely that citation bias has had any influence on the results. Although the initial study selection was performed by one author, two authors independently assessed the potentially relevant studies for inclusion, and all studies included in previous reviews were identified by the search as well as additional studies that were missed by previous reviews, suggesting that selection bias is also less likely to be an issue.

The number of studies that investigated specific types of nuts was limited; thus, any further studies should try to clarify associations between specific subtypes of nuts and cardiovascular disease, cancer, and mortality. However, randomized controlled trials have suggested similar effects of different types of nuts on cardiovascular risk factors [4], which supports our findings with regard to peanuts and tree nuts in relation to coronary heart disease and cardiovascular disease and overall mortality, although only tree nuts were associated with reduced risk of cancer. The PREDIMED study suggested similar associations between walnuts and all other nuts in relation to cardiovascular disease and all-cause mortality; however, walnuts were more strongly associated with cancer mortality than all other nuts [21]. As in any meta-analysis of published studies, publication bias could have influenced the results; however, there was evidence of publication bias only in the analysis of all-cause mortality, but after exclusion of four studies with less than 500 deaths [10, 18, 21, 22], the test for publication bias was not significant, but the summary estimates remained similar, thus publication bias has most likely not substantially influenced the overall findings.

Although results from observational studies alone cannot be used to draw conclusions with regards to whether the observed associations are causal, one randomized trial (the PREDIMED study) also found a reduced risk of cardiovascular disease in subjects randomized to a Mediterranean diet with nuts compared to subjects randomized to a control diet [64]; however, it is not clear if this association is due to the Mediterranean diet component, nuts, or a combination of the two. In addition, several mechanisms might explain the beneficial effect observed between nut intake and cardiovascular disease, total cancer, and all-cause mortality. Nuts are good sources of unsaturated fatty acids, protein, fiber, vitamin E, potassium, magnesium, and phytochemicals. Intervention studies have shown that nut consumption reduces total cholesterol, low-density lipoprotein cholesterol, and the ratio of low- to high-density lipoprotein cholesterol, and ratio of total to high-density lipoprotein cholesterol, apolipoprotein B, and triglyceride levels in a dose–response manner [4, 65]. In addition, studies have shown reduced endothelial dysfunction [8], lipid peroxidation [7], and insulin resistance [6, 66] with a higher intake of nuts. Oxidative damage and insulin resistance are important pathogenic drivers of cancer [67, 68] and a number of specific causes of death [69]. Nuts and seeds and particularly walnuts, pecans, and sunflower seeds have a high antioxidant content [70], and could prevent cancer by reducing oxidative DNA damage [9], cell proliferation [71, 72], inflammation [73, 74], and circulating insulin-like growth factor 1 concentrations [75] and by inducing apoptosis [71], suppressing angiogenesis [76], and altering the gut microbiota [77]. Although nuts are high in total fat, they have been associated with lower weight gain [78–80] and lower risk of overweight and obesity [79] in observational studies and some randomized controlled trials [80]. However, the inverse associations observed between nut intake and chronic disease in this meta-analysis persisted among studies that adjusted for BMI, suggesting an association independent of BMI. In addition, it is possible that a high intake of nuts may reduce the severity of disease and progression to death as indicated by studies which found that patients with diabetes, coronary heart disease, and heart failure who consumed more nuts had a reduced risk of cardiovascular disease or all-cause mortality [81–83]. Although we observed similar associations for both incidence and mortality from coronary heart disease and cardiovascular disease, epidemiological studies on nut consumption and type 2 diabetes incidence have largely shown no association [29], while in the present meta-analysis we found a reduced risk of diabetes mortality, which might be due to reduced risk of cardio-metabolic risk factors and complications in diabetes patients with a high nut intake [83–85].

Strengths of the current meta-analysis include the comprehensive and up-to-date search strategy that identified several large additional studies; inclusion of prospective studies, which limits the possibilities for certain biases; the detailed dose–response analyses, which clarified the amount of nut intake needed to reduce the risk of cardiovascular disease, cancer, and all-cause and cause-specific mortality; the consistency and robustness of the findings in a number of subgroup and sensitivity analyses; the high study quality of the included studies; and the large number of participants, which provided statistical power to detect moderate associations between nut consumption and different health outcomes. The observation that nut intake was inversely associated with these outcomes in both men and women and when stratified by geographic location, is a further strength of the analysis.

We estimated that approximately 4.4 million deaths in the regions included may be attributable to nut consumption below 20 grams per day. As shown in the current meta-analysis, nut consumption appears to reduce the risk of coronary heart disease, cardiovascular disease overall, total cancer, all-cause mortality, and mortality from respiratory disease, diabetes and possibly other causes, so using data on nut intake and all-cause mortality will provide a more comprehensive assessment of the mortality burden due to low nut consumption than only analysing mortality from specific diseases (such as coronary heart disease and cancer), at least for regions with a similar distribution of specific causes of death as those included in the meta-analysis. Another limitation is that, for the calculation of the population-attributable risk, we did not find dietary surveys on nut intake from some regions, including Africa, the Middle East and West-Asia, and therefore, in a global context, these estimates are conservative.

These findings support recommendations to increase intake of nuts to reduce risk of chronic diseases and premature mortality in the general population. However, in regions of the world where nuts are a major source of aflatoxin [86], increasing nut intake should only be recommended as long as aflatoxin contamination is avoided in the affected countries. Any further studies should investigate associations with specific types of nuts and the association between nut intake and incidence and mortality from less common diseases, adjust for more dietary confounders, clarify mechanisms underlying non-cardiovascular causes of death, and investigate associations between biomarkers of nut consumption in relation to health outcomes [87, 88]. In addition, further studies from other regions of the world are also needed.

## Conclusions

In conclusion, our results provide further evidence that nut consumption may reduce the risk of coronary heart disease, stroke, cardiovascular disease, total cancer, and all-cause mortality, and possibly mortality from diabetes, respiratory disease, and infectious disease. In 2013, an estimated 4.4 million deaths may be attributable to a nut intake below 20 grams per day in North and South America, Europe, Southeast Asia, and the Western Pacific. These findings support dietary recommendations to increase nut consumption to reduce chronic disease risk and mortality.

## Additional file

Additional file 1: Supplementary figures and tables. (PDF 1540 kb)

## Abbreviations

BMI: Body mass index
CI: Confidence interval
RR: Relative risk
