# Supplementary material for: Nut consumption and risk of cardiovascular disease, total cancer, all-cause and cause-specific mortality: a systematic review and dose-response meta-analysis of prospective studies
Source: BMC Med. 2016 Dec 5;14:207. doi: 10.1186/s12916-016-0730-3 (PMC5137221; doi:10.1186/s12916-016-0730-3)
Supplement: Additional file 1: — Supplementary figures and tables. (PDF 1540 kb) [file 12916_2016_730_MOESM1_ESM.pdf]

Supplementary Figures and Tables for Aune D, Keum N, Giovannucci E, Fadnes LT, Boffetta P, Greenwood DC, Tonstad S, Vatten LJ, Riboli E. Norat T. Nut consumption and risk of cardiovascular disease, cancer, all-cause and cause-specific mortality – a systematic review and dose-response meta-analysis of prospective studies. BMC Medicine 2016; DOI 10.1186/s12916-016-0730-3.

Supplementary Table 1. Search strategy in PubMed

1. fruits
2. vegetables
3. fruit
4. vegetable
5. berry
6. berries
7. citrus
8. “citrus fruits”
9. cruciferae
10. “cruciferous vegetables”
11. cabbages
12. “allium vegetables”
13. strawberry
14. strawberries
15. tomato
16. tomatoes
17. cereal
18. cereals
19. “breakfast cereal”
20. grain
21. grains
22. “whole grain”
23. “whole grains”
24. rice
25. bread
26. nut
27. seed
28. peanut
29. peanuts
30. legumes
31. soy
32. soya
33. chickpeas
34. chickpea
35. bean
36. beans
37. lentil
38. legume
39. legumes
40. fiber
41. “dietary fiber”
42. “fruit fiber”
43. “vegetable fiber”
44. “legume fiber”
45. “cereal fiber”
46. fibre
47. “dietary fibre”
48. “fruit fibre”
49. “vegetable fibre”
50. “cereal fibre”
51. “DASH diet”
52. diet
53. foods
54. “dietary patterns”
55. “dietary pattern”
56. “dietary score”
57. “diet score”

58. “diet index”
59. “food index”
60. “nutrient index”
61. “Mediterranean diet”
62. “vitamin C”
63 “ascorbic acid”
64. “vitamin E”
65. carotenoids
66. carotenoid
67. flavonoid
68. flavonoids
69. (1 OR 2 OR 3 OR 4 OR 5 OR 6 OR 7 OR 8 OR 9 OR 10 OR 11 OR 12 OR 13 OR 14 OR 15 OR 16 OR 17 OR 18 OR 19 OR 20 OR 21 OR 22 OR 23 OR 24 OR 25 OR 26 OR 27 OR 28 OR 29 OR 30 OR 31 OR 32 OR 33 OR 34 OR 35 OR 36 OR 37 OR 38 OR 39 OR 40 OR 41 OR 42 OR 43 OR 44 OR 45 OR 46 OR 47 OR 48 OR 49 OR 50 OR 51 OR 52 OR 53 OR 54 OR 55 OR 56 OR 57 OR 58 OR 59 OR 60 OR 61 OR 62 OR 63 OR 64 OR 65 OR 66 OR 67 OR 68)
70. “coronary heart disease”
71. “heart disease”
72. “ischemic heart disease”
73. “ischaemic heart disease”
74. CHD
75. “coronary artery disease”
76. “myocardial infarction”
77. stroke
78. “ischemic stroke”
79. “haemorrhagic stroke”
80. “cardiovascular disease”
81. CVD
82. cancer
83. “total cancer”
84. mortality
85. “all-cause mortality”
86. “total mortality”
87. survival
88. (70 OR 71 OR 72 OR 73 OR 74 OR 75 OR 76 OR 77 OR 78 OR 79 OR 80 OR 81 OR 82 OR 83 OR 84 OR 85 OR 86 OR 87)
89. “case-control”
90. cohort
91. cohorts
92. prospective
93. longitudinal
94. retrospective
95. “follow-up”
96. “cross-sectional”
97. “population-based”
98. “relative risk
99. “odds ratio”
100 “hazard ratio”
101 “incidence rate ratio”
102 (89 OR 90 OR 91 OR 92 OR 93 OR 94 OR 95 OR 96 OR 97 OR 98 OR 99 OR 100 OR 101)
103. 69 AND 88 AND 102

Supplementary Table 2. List of excluded studies and reason for exclusion

Exclusion reason	Reference number
Abstract only publication	(1-3)
Duplicates	(4-13)
Ecological study	(14)
Letter	(15-17)
Meta-analysis	(18-29)
No risk estimates	(30;31)
Not relevant outcome	(32-34)
Not usable result	(35;36)
Patient population (diabetes, heart disease, heart failure	(37-39)
Review	(40-53)
Unspecific exposure (combined with fruits or legumes)	(54-60)

Reference List

1. Fernandez MA, Bes RM, Beunza JJ et al. Nut consumption and decreased risk of total mortality in the sun project. Annals of Nutrition and Metabolism 2013;63:1017.

2. Guasch-Ferre M, Bullo M, Martinez-Gonzalez M-A et al. Frequency of nut consumption and risk of total mortality in the predimed study. Annals of Nutrition and Metabolism 2013;62:17-8.

3. Djousse L, Petrone A, Gaziano J. Nut consumption is associated with a lower risk of death among us male physicians. Circulation 2014;129:25 (AP067).

4. Fraser GE, Lindsted KD, Beeson WL. Effect of risk factor values on lifetime risk of and age at first coronary event. The Adventist Health Study. Am J Epidemiol 1995;142:746-58.

5. Hu FB, Stampfer MJ, Manson JE et al. Frequent nut consumption and risk of coronary heart disease in women: prospective cohort study. BMJ 1998;317:1341-5.

6. Fraser GE. Nut consumption, lipids, and risk of a coronary event. Asia Pac J Clin Nutr 2000;9 Suppl 1:S28-S32.

7. Ellsworth JL, Kushi LH, Folsom AR. Frequent nut intake and risk of death from coronary heart disease and all causes in postmenopausal women: the Iowa Women's Health Study. Nutr Metab Cardiovasc Dis 2001;11:372-7.

8. He K, Merchant A, Rimm EB et al. Dietary fat intake and risk of stroke in male US healthcare professionals: 14 year prospective cohort study. BMJ 2003;327:777-82.

9. van den Brandt PA. The impact of a Mediterranean diet and healthy lifestyle on premature mortality in men and women. Am J Clin Nutr 2011;94:913-20.

10. Baer HJ, Glynn RJ, Hu FB et al. Risk factors for mortality in the nurses' health study: a competing risks analysis. Am J Epidemiol 2011;173:319-29.

11. Sluik D, Boeing H, Li K et al. Lifestyle factors and mortality risk in individuals with diabetes mellitus: are the associations different from those in individuals without diabetes? Diabetologia 2014;57:63-72.

12. Schroder H, Salas-Salvado J, Martinez-Gonzalez MA et al. Baseline adherence to the Mediterranean diet and major cardiovascular events: Prevencion con Dieta Mediterranea trial. JAMA Intern Med 2014;174:1690-2.

13. Kushi LH, Folsom AR, Prineas RJ, Mink PJ, Wu Y, Bostick RM. Dietary antioxidant vitamins and death from coronary heart disease in postmenopausal women. N Engl J Med 1996;334:1156-62.

14. Papandreou C, Tuomilehto H. Coronary heart disease mortality in relation to dietary, lifestyle and biochemical risk factors in the countries of the Seven Countries Study: a secondary dataset analysis. Journal of human nutrition and dietetics : the official journal of the British Dietetic Association 2014;27:168-75.

15. Kopel E, Kivity S, Sidi Y. Nut consumption and mortality [6]. New England Journal of Medicine 2014;370:881-2.

16. Temple NJ. Re. "Nut consumption and 5-y all-cause mortality in a Mediterranean cohort: The SUN project". Nutrition 2015;31:1299.

17. Fernandez-Montero A, Martinez-Gonzalez MA, Moreno-Galarraga L. Re. "Nut consumption and 5-y all-cause mortality in a Mediterranean cohort: The SUN project": Authors' response. Nutrition 2015;31:1299-300.

18. Mente A, de KL, Shannon HS, Anand SS. A systematic review of the evidence supporting a causal link between dietary factors and coronary heart disease. Arch Intern Med 2009;169:659-69.

19. Luo C, Zhang Y, Ding Y et al. Nut consumption and risk of type 2 diabetes, cardiovascular disease, and all-cause mortality: a systematic review and meta-analysis. Am J Clin Nutr 2014;100:256-69.

20. Ma L, Wang F, Guo W, Yang H, Liu Y, Zhang W. Nut consumption and the risk of coronary artery disease: a dose-response meta-analysis of 13 prospective studies. Thromb Res 2014;134:790-4.

21. Weng Y-Q, Yao J, Guo M-L, Qin Q-J, Li P. Association between nut consumption and coronary heart disease: A meta-analysis. Experimental and Clinical Cardiology 2014;20:NA.

22. Zhou D, Yu H, He F et al. Nut consumption in relation to cardiovascular disease risk and type 2 diabetes: a systematic review and meta-analysis of prospective studies. Am J Clin Nutr 2014;100:270-7.

23. Afshin A, Micha R, Khatibzadeh S, Mozaffarian D. Consumption of nuts and legumes and risk of incident ischemic heart disease, stroke, and diabetes: a systematic review and meta-analysis. Am J Clin Nutr 2014;100:278-88.

24. Shi ZQ, Tang JJ, Wu H, Xie CY, He ZZ. Consumption of nuts and legumes and risk of stroke: a meta-analysis of prospective cohort studies. Nutr Metab Cardiovasc Dis 2014;24:1262-71.

25. Grosso G, Yang J, Marventano S, Micek A, Galvano F, Kales SN. Nut consumption on all-cause, cardiovascular, and cancer mortality risk: a systematic review and meta-analysis of epidemiologic studies. Am J Clin Nutr 2015;101:783-93.

26. Wu L, Wang Z, Zhu J, Murad AL, Prokop LJ, Murad MH. Nut consumption and risk of cancer and type 2 diabetes: a systematic review and meta-analysis. Nutr Rev 2015;73:409-25.

27. Zhang Z, Xu G, Wei Y, Zhu W, Liu X. Nut consumption and risk of stroke. Eur J Epidemiol 2015;30:189-96.

28. Weng YQ, Yao J, Guo ML, Qin QJ, Li P. Association between nut consumption and coronary heart disease: a meta-analysis. Coron Artery Dis 2016;27:227-32.

29. Mayhew AJ, de Souza RJ, Meyre D, Anand SS, Mente A. A systematic review and meta-analysis of nut consumption and incident risk of CVD and all-cause mortality. Br J Nutr 2016;115:212-25.

30. Trichopoulou A, Kouris-Blazos A, Vassilakou T et al. Diet and survival of elderly Greeks: a link to the past. Am J Clin Nutr 1995;61:1346S-50S.

31. Fraser GE, Shavlik DJ. Ten years of life is it a matter of choice? Archives of Internal Medicine 2001;161:1645-52.

32. Nettleton JA, Steffen LM, Loehr LR, Rosamond WD, Folsom AR. Incident heart failure is associated with lower whole-grain intake and greater high-fat dairy and egg intake in the Atherosclerosis Risk in Communities (ARIC) study. J Am Diet Assoc 2008;108:1881-7.

33. Djousse L, Rudich T, Gaziano JM. Nut consumption and risk of hypertension in US male physicians. Clin Nutr 2009;28:10-4.

34. Greco G, Egorova NN, Moskowitz AJ et al. A model for predicting the risk of carotid artery disease. Ann Surg 2013;257:1168-73.

35. Akbaraly TN, Ferrie JE, Berr C et al. Alternative Healthy Eating Index and mortality over 18 y of follow-up: results from the Whitehall II cohort. Am J Clin Nutr 2011;94:247-53.

36. Reedy J, Krebs-Smith SM, Miller PE et al. Higher diet quality is associated with decreased risk of all-cause, cardiovascular disease, and cancer mortality among older adults. J Nutr 2014;144:881-9.

37. Li TY, Brennan AM, Wedick NM, Mantzoros C, Rifai N, Hu FB. Regular consumption of nuts is associated with a lower risk of cardiovascular disease in women with type 2 diabetes. J Nutr 2009;139:1333-8.

38. Trichopoulou A, Bamia C, Trichopoulos D. Mediterranean diet and survival among patients with coronary heart disease in Greece. Arch Intern Med 2005;165:929-35.

39. Levitan EB, Lewis CE, Tinker LF et al. Mediterranean and DASH Diet Scores and Mortality in Women with Heart Failure: The Women's Health Initiative. Circ Heart Fail 2013;6:1116-23.

40. Sabate J. Does nut consumption protect against ischaemic heart disease? Eur J Clin Nutr 1993;47 Suppl 1:S71-S75.

41. Hu FB, Stampfer MJ. Nut consumption and risk of coronary heart disease: a review of epidemiologic evidence. Curr Atheroscler Rep 1999;1:204-9.

42. Fraser GE. Nut consumption, lipids, and risk of a coronary event. Clin Cardiol 1999;22:III11-III15.

43. Fraser GE. Diet as primordial prevention in Seventh-Day Adventists. Prev Med 1999;29:S18-S23.

44. Fraser GE. Associations between diet and cancer, ischemic heart disease, and all-cause mortality in non-Hispanic white California Seventh-day Adventists. Am J Clin Nutr 1999;70:532S-8S.

45. Sabate J. Nut consumption, vegetarian diets, ischemic heart disease risk, and all-cause mortality: evidence from epidemiologic studies. Am J Clin Nutr 1999;70:500S-3S.

46. Hu FB, Willett WC. Optimal diets for prevention of coronary heart disease. JAMA 2002;288:2569-78.

47. Feldman EB. The scientific evidence for a beneficial health relationship between walnuts and coronary heart disease. J Nutr 2002;132:1062S-101S.

48. Hu FB. Plant-based foods and prevention of cardiovascular disease: an overview. Am J Clin Nutr 2003;78:544S-51S.

49. Megias-Rangil I, Garcia-Lorda P, Torres-Moreno M, Bullo M, Salas-Salvado J. Nutrient content and health effects of nuts. [Spanish]. Archivos latinoamericanos de nutricion 2004;54:83-6.

50. Kelly JH, Jr., Sabate J. Nuts and coronary heart disease: an epidemiological perspective. Br J Nutr 2006;96 Suppl 2:S61-S67.

51. Sabate J, Ang Y. Nuts and health outcomes: new epidemiologic evidence. Am J Clin Nutr 2009;89:1643S-8S.

52. Ros E, Tapsell LC, Sabate J. Nuts and berries for heart health. Current Atherosclerosis Reports 2010;12:397-406.

53. Grosso G, Estruch R. Nut consumption and age-related disease. Maturitas 2016;84:11-6.

54. Knoops KT, Groot de LC, Fidanza F, berti-Fidanza A, Kromhout D, van Staveren WA. Comparison of three different dietary scores in relation to 10-year mortality in elderly European subjects: the HALE project. Eur J Clin Nutr 2006;60:746-55.

55. Benetou V, Trichopoulou A, Orfanos P et al. Conformity to traditional Mediterranean diet and cancer incidence: the Greek EPIC cohort. Br J Cancer 2008;99:191-5.

56. Boggs DA, Ban Y, Palmer JR, Rosenberg L. Higher diet quality is inversely associated with mortality in African-American women. J Nutr 2015;145:547-54.

57. Kouris-Blazos A, Gnardellis C, Wahlqvist ML, Trichopoulos D, Lukito W, Trichopoulou A. Are the advantages of the Mediterranean diet transferable to other populations? A cohort study in Melbourne, Australia. Br J Nutr 1999;82:57-61.

58. Key TJ, Thorogood M, Appleby PN, Burr ML. Dietary habits and mortality in 11,000 vegetarians and health conscious people: results of a 17 year follow up. BMJ 1996;313:775-9.

59. Dilis V, Katsoulis M, Lagiou P, Trichopoulos D, Naska A, Trichopoulou A. Mediterranean diet and CHD: the Greek European Prospective Investigation into Cancer and Nutrition cohort. Br J Nutr 2012;108:699-709.

60. Estruch R, Ros E, Salas-Salvado J et al. Primary prevention of cardiovascular disease with a Mediterranean diet. N Engl J Med 2013;368:1279-90.

Supplementary table 3. Nut consumption and coronary heart disease

Author, publication year, country	Study name	Study period	Number of participants, gender, age, number of cases/deaths	Dietary assessment	Exposure and subgroup	Nut consumption frequency or amount	Relative risks (95% confidence intervals)	Adjustment for confounding factors
Fraser, 1992, USA	Adventist Health Study	1976-1982, 6 years follow-up	31208 men and women, age ≥25 years:134 incident nonfatal MI<br>260 definite fatal myocardial infarction<br>463 coronary deaths	Validated FFQ, 65 food items	Nuts, incident nonfatal MI<br><br><br>Nuts, definite fatal MI<br><br><br>Nuts, coronary deaths	<1/wk<br>1-4/wk<br>≥5/wk<br><1/wk<br>1-4/wk<br>≥5/wk<br><1/wk<br>1-4/wk<br>≥5/wk	1.00<br>0.78 (0.51-1.18)<br>0.49 (0.28-0.85)<br>1.00<br>0.76 (0.56-1.04)<br>0.52 (0.36-0.76)<br>1.00<br>0.82 (0.65-1.04)<br>0.59 (0.45-0.78)	Age, sex, smoking, exercise, relative weight, high blood pressure, type of grains – whole wheat vs. white, beef
Mann JI, 1997, England	The Oxford Vegetarian Study	1980-1984 – 1995, 13.3 years follow-up	10802 men and women, age 16-79 years: 64 IHD deaths	FFQ	Nuts	<1/wk<br>1-4<br>≥5	1.00<br>1.19 (0.68-2.10)<br>0.87 (0.45-1.68)	Age, sex, smoking, social class
Albert CM et al, 2002, USA	Physicians' Health Study	1982-NA, 17 years follow-up	21454 men, age 40-84 years: 566 CHD deaths<br>1037 nonfatal MIs	FFQ, 20 food items (validated in other studies)	Nuts, CHD death<br><br><br><br>Nuts, nonfatal MI	<1/mo<br>1-3/mo<br>1/wk<br>≥2/wk<br><1/mo<br>1-3/mo<br>1/wk<br>≥2/wk	1.00<br>0.89 (0.67-1.16)<br>0.90 (0.67-1.22)<br>0.70 (0.50-0.98)<br>1.00<br>1.22 (1.00-1.51)<br>1.20 (0.96-1.50)<br>1.04 (0.82-1.33)	Age, aspirin and beta-carotene assignment, cardiovascular disease, cigarette smoking, alcohol intake, physical activity, BMI, DM, high cholesterol, hypertension, use of multivitamins, vitamin E, vitamin C, fish, red meat, fruit/vegetables, dairy intake
Blomhoff R et al, 2006, USA	Iowa Women's Health Study	1986-2001, 15 years follow-up	31778 women, age 55-69 years: 948 CHD deaths	FFQ, 127 food items (validated in other studies)	Nuts and peanut butter	0.0/wk<br>0.5<br>1.5<br>7.0	1.00<br>1.03 (0.84-1.26)<br>0.82 (0.68-0.98)<br>0.71 (0.55-0.91)	Age, energy intake, BMI, WHR, physical activity, HRT, multivitamin supplements, alcohol, whole grain, refined grain, red meat, fish and seafood, total fruits and vegetables
Bernstein AM et al, 2010, USA	Nurses' Health Study	1980-2006, 26 years follow-up	84136 women, age 34-59 years: 2210 nonfatal MIs and 952 CHD deaths	Validated FFQ	Nuts	0.0 serv/d<br>0.04<br>0.07<br>0.12	1.00<br>0.73 (0.65-0.82)<br>0.91 (0.82-1.00)<br>0.76 (0.67-0.84)	Age, time period, total energy, alcohol, trans fat, BMI, cigarette smoking status an cigarettes per day, menopausal status, HRT, parental history of MI <65

						0.40<br>Per 1 serv/d	0.68 (0.60-0.76)<br>0.78 (0.66-0.93)	years age, multivitamin use, vitamin E supplement use, aspirin use, physical exercise
Bao Y et al, 2013, USA	Nurses' Health Study	1980-2010, 30 years follow-up	76464 women, age 34-59 years: 2208 CHD deaths	Validated FFQ, 61-116 food items	Nuts, heart disease<br><br><br><br><br>Peanuts, heart disease<br><br><br><br>Tree nuts	Never<br><1/wk<br>1<br>2-4<br>≥5<br>Never<br><1/wk<br>1<br>≥2<br>Never<br><1/wk<br>1<br>≥2	1.00<br>0.84 (0.76-0.94)<br>0.79 (0.68-0.90)<br>0.76 (0.65-0.88)<br>0.72 (0.55-0.94)<br>1.00<br>0.89 (0.79-1.00)<br>0.91 (0.75-1.11)<br>0.81 (0.65-1.01)<br>1.00<br>0.83 (0.74-0.93)<br>1.00 (0.82-1.23)<br>0.73 (0.56-0.96)	Age, race, BMI, physical activity, smoking, screening, multivitamin use, aspirin use, FH – DM, MI or cancer, history of DM, hypertension, or hypercholesterolemia, total energy, alcohol, red or processed meat, fruits, vegetables, menopausal status, HRT
Bao Y et al, 2013, USA	Health Professionals Follow-up Study	1986-2010, 24 years follow-up	42498 men, age 45-70 years: 2698 CHD deaths	Validated FFQ, 131 food items	Nuts, heart disease<br><br><br><br><br>Peanuts, heart disease<br><br><br><br>Tree nuts	Never<br><1/wk<br>1<br>2-4<br>≥5<br>Never<br><1/wk<br>1<br>≥2<br>Never<br><1/wk<br>1<br>≥2	1.00<br>0.83 (0.74-0.94)<br>0.78 (0.69-0.88)<br>0.74 (0.66-0.84)<br>0.71 (0.61-0.83)<br>1.00<br>0.84 (0.76-0.93)<br>0.83 (0.73-0.94)<br>0.74 (0.66-0.84)<br>1.00<br>0.85 (0.77-0.93)<br>0.85 (0.74-0.97)<br>0.76 (0.67-0.87)	Age, race, BMI, physical activity, smoking, screening, multivitamin use, aspirin use, FH – DM, MI or cancer, history of DM, hypertension, or hypercholesterolemia, total energy, alcohol, red or processed meat, fruits, vegetables
Haring B et al, 2014, USA	Atherosclerosis Risk In Communities Study (ARIC)	1987-1989 - 2010, 22 years follow-up	12066 men and women, age 45-64 years: 1147 CHD cases	FFQ, 66 food items	Nuts	0.0 serv/d<br>0.1<br>0.2<br>0.4<br>1.0	1.00<br>0.89 (0.75-1.06)<br>0.86 (0.71-1.05)<br>0.83 (0.68-1.01)<br>0.91 (0.74-1.12)	Age, sex, race, study center, total energy intake, smoking, education, SBP, antihypertensive medication, HDL cholesterol, total cholesterol, use of lipid lowering medication, BMI, WHR, alcohol, sports-related physical activity, leisure-time physical activity, carbohydrate intake, fiber intake, magnesium intake
Hshieh TT et al,	Physicians'	1999-2002	20742 men, mean	FFQ, 19	Nuts	<1 serv/mo	1.00	Age, BMI, alcohol, smoking, exercise,

2015, USA	Health Study	– NA, 9.6 years follow-up	age 66 years: 405 CAD deaths	food items (validated in other cohorts)		1-3<br>1 serv/wk<br>2-4<br>≥5	1.02 (0.80-1.30)<br>0.88 (0.66-1.19)<br>0.72 (0.50-1.04)<br>0.85 (0.56-1.28)	calories, SFA, fruit and vegetables, red meat, prevalent DM, hypertension
Luu HN et al, 2015, USA	Southern Community Cohort Study	2002-2009-NA, 5.4 years follow-up	71764 men and women, age 40-79 years: 793 CHD deaths	FFQ, 89 food items	Total nuts and peanut butter, African Americans<br><br><br><br><br>Total nuts and peanut butter, Caucasians	<0.95 g/d<br>0.95-<3.08<br>3.08-<7.30<br>7.30-<18.45<br>≥18.45<br><0.95 g/d<br>0.95-<3.08<br>3.08-<7.30<br>7.30-<18.45<br>≥18.45	1.00<br>0.67 (0.51-0.88)<br>0.95 (0.72-1.25)<br>0.74 (0.55-0.98)<br>0.62 (0.45-0.85)<br>1.00<br>0.85 (0.59-1.24)<br>0.73 (0.50-1.06)<br>0.65 (0.44-0.97)<br>0.60 (0.39-0.92)	Age, sex, race, education, occupation, household income, marital status, smoking pack-years, alcohol, BMI, physical activity, vitamin supplement use, Charlson Comorbidity Index, metabolic conditions (hypertension, heart disease, DM, obesity, hypercholesterolemia), total energy, red meat, chicken and duck intake, seafood, vegetables, fruits
Luu HN et al, 2015, China	Shanghai Men's Health Study	2002-2006 - NA, 6.5 years follow-up	61123 men, age 40-74 years: 306 CHD deaths	Validated FFQ, 84 food items	Peanuts	<0.14 g/d<br>0.14-<0.72<br>0.72-<1.45<br>1.45-<2.54<br>・2.54	1.00<br>0.93 (0.60-1.44)<br>0.66 (0.44-0.97)<br>0.81 (0.59-1.10)<br>0.80 (0.58-1.11)	Age, race, education, occupation, household income, marital status, smoking pack-years, alcohol, BMI, physical activity, vitamin supplement use, Charlson Comorbidity Index, metabolic conditions (hypertension, heart disease, DM, obesity, dyslipidemia), total energy, red meat, chicken and duck intake, seafood, vegetables, fruits
Luu HN et al, 2015, China	Shanghai Women's Health Study	1996-2000 – NA, 12.2 years follow-up	73142 women, age 40-70 years: 325 CHD deaths	Validated FFQ, 87 food items	Peanuts	<0.14 g/d<br>0.14-<0.72<br>0.72-<1.45<br>1.45-<2.54<br>≥2.54	1.00<br>0.91 (0.66-1.26)<br>0.83 (0.61-1.14)<br>0.70 (0.50-0.97)<br>0.58 (0.39-0.87)	Age, race, education, occupation, household income, marital status, smoking pack-years, alcohol, BMI, physical activity, vitamin supplement use, Charlson Comorbidity Index, metabolic conditions (hypertension, heart disease, DM, obesity, dyslipidemia), total energy, red meat, chicken and duck intake, seafood, vegetables, fruits
van den Brandt PA et al, 2015, Netherlands	Netherlands Cohort Study	1986-1996, 10 years follow-up	3202 subcohort members, men and women, age 55-69 years: 1488 IHD deaths	Validated FFQ, 150 food items	Total nuts, all<br><br><br><br>Peanuts	0 g/d<br>0.1-<5<br>5-<10<br>≥10<br>0 g/d	1.00<br>0.90 (0.76-1.07)<br>0.67 (0.52-0.88)<br>0.83 (0.67-1.04)<br>1.00	Age, sex, cigarette smoking, number of cigarettes per day, years of smoking, hypertension, DM, body height, BMI, non-occupational physical activity, highest level of education, alcohol,

						0.1-<5<br>≥5	0.86 (0.73-1.03)<br>0.79 (0.64-0.96)	vegetables, fruits, energy, nutritional supplement use, women: HRT
					Tree nuts	0 g/d<br>0.1-<5<br>≥5	1.00<br>0.88 (0.74-1.06)<br>1.03 (0.72-1.46)	
					Peanut butter	0 g/d<br>0.1-<5<br>≥5	1.00<br>1.04 (0.86-1.26)<br>0.97 (0.75-1.24)	
Bonaccio M et al, 2015, Italy	The Moli-sani Study	2005-2010 – 2011, 4.3 years follow-up	19386 men and women, mean age 54.5 years: 39 CHD deaths	Validated FFQ, 188 food items	Nuts	Never<br>Ever	1.00<br>0.74 (0.38-1.45)	Age, sex, education, smoking status, leisure-time physical activity, BMI, energy intake, Mediterranean diet score without nuts
Gopinath B et al, 2015, Australia	The Blue Mountains Eye Study	1992-1994 - 2007, 15 years follow-up	2893 men and women, age ≥49 years: 430 IHD deaths	Validated FFQ, 145 food items	Nuts, all	0-0.50 g/d<br>0.90-4.55<br>4.90-100	1.00<br>0.77 (0.60-0.98)<br>0.95 (0.74-1.21)	Age, sex, qualifications, total diet score (including food groups – vegetables, fruit, cereals and breads, meat, fish, poultry, dairy, sodium, alcohol, sugar, extra foods and energy intake and physical activity level), BMI, current smoking status, alcohol, self-rated health, walking disability, hypertension, diabetes, doctor-diagnosed history of cancer, angina, stroke, acute myocardial infarction
					Nuts, women	0-0.50 g/d<br>0.90-4.55<br>4.90-100	1.00<br>0.66 (0.45-0.97)<br>1.02 (0.70-1.49)	
					Nuts, men	0-0.50 g/d<br>0.90-4.55<br>4.90-100	1.00<br>0.81 (0.59-1.11)<br>0.91 (0.66-1.25)	
Wang JB et al, 2016, China	Linxian Nutrition Intervention Trial cohort	1984-1991 - 2010, 19-26 years follow-up	2445 men and women, age 40-69 years: 355 heart disease deaths	FFQ, 64 food items	Nuts	Per 3 times/mo	0.89 (0.82-0.98)	Age, sex, commune, smoking, drinking, season, BMI

BMI=body mass index, CHD=coronary heart disease, DM=diabetes mellitus, FFQ=food frequency questionnaire, FH=family history, HRT=hormone replacement therapy, IHD=ischemic heart disease, MI=myocardial infarction, NA=not available, SFA=saturated fatty acids, WHR=waist-to-hip ratio

Supplementary table 4. Nut consumption and stroke

Author, publication year, country	Study name	Study period	Number of participants, gender, age, number of cases/deaths	Dietary assessment	Exposure and subgroup	Nut consumption frequency or amount	Relative risks (95% confidence intervals)	Adjustment for confounding factors
Yochum LA et al, 2000, USA	Iowa Women's Health Study	1986-1997, ~11.4 years follow-up	34492 women, age 55-69 years: 215 stroke deaths	FFQ, 127 food items (validated in other cohorts)	Nuts and seeds	0 times/mo<br>1-2<br>3-4<br>>4	1.00<br>0.85 (0.61-1.18)<br>0.79 (0.50-1.24)<br>0.73 (0.41-1.29)	Age, total energy intake, BMI, WHR, high blood pressure, DM, HRT, alcohol, education, marital status, pack-years of smoking, physical activity, cholesterol, SFA, fish, vitamin C, carotenoids, dietary fiber, whole grains
Djousse L et al, 2010, USA	Physicians' Health Study	1982-2008, 21.1 years follow-up	21078 men and women, age 40.7-86.7 years: 1424 stroke cases	FFQ, 19 food items (validated in other cohorts)	Nuts, total stroke	0 times/wk<br><1<br>1<br>2-4<br>5-6<br>≥7	1.00<br>0.91 (0.79-1.05)<br>0.95 (0.81-1.11)<br>0.90 (0.75-1.08)<br>1.11 (0.85-1.46)<br>1.07 (0.79-1.46)	Age, aspirin assignment, BMI, alcohol, smoking, fruit and vegetables, regular exercise, breakfast cereal, red meat, fish, dairy, hypertension, DM, atrial fibrillation, coronary heart disease
					Nuts, ischemic stroke	0 times/wk<br><1<br>1<br>2-4<br>5-6<br>≥7	1.00<br>0.86 (0.74-1.01)<br>0.94 (0.79-1.11)<br>0.97 (0.80-1.18)<br>1.06 (0.79-1.43)<br>0.93 (0.65-1.34)	
					Nuts, hemorrhagic stroke	0 times/wk<br><1<br>1<br>2-4<br>5-6<br>≥7	1.00<br>1.13 (0.78-1.62)<br>1.05 (0.70-1.58)<br>0.49 (0.27-0.89)<br>1.50 (0.79-2.84)<br>1.84 (0.95-3.57)	
Yaemsiri S et al, 2012, USA	Women's Health Initiative	1994-1998 – 2005, 7.6 years follow-up	87025 women, age 50-79 years: 1049 ischemic stroke cases	Validated FFQ, 122 food items	Nuts, total ischemic stroke	Per 1 serv/d	0.85 (0.64-1.14)	Age, race, education, family income, smoking status and years as regular smoker, HRT use, total metabolic equivalent task hours per week, alcohol intake, history of coronary heart disease, atrial fibrillation, DM, aspirin use, antihypertensive medication, cholesterol-lowering medication, BMI, systolic blood pressure, total energy
					Nuts, atherothrombotic stroke	Per 1 serv/d	0.89 (0.35-2.24)	
					Nuts, lacunar stroke	Per 1 serv/d	0.79 (0.44-1.42)	
					Nuts, cardioembolic stroke	Per 1 serv/d	0.78 (0.41-1.45)	

Bernstein AM et al, 2012, USA	Health Professionals Follow-up Study	1986-2008, 22 years follow-up	43150 men, age 40-75 years: 1397 stroke cases	Validated FFQ, 131 food items	Nuts	0.00 serv/d<br>0.07<br>0.14<br>0.25<br>0.60<br>Per 1 serv/d	1.00<br>0.94 (0.79-1.12)<br>0.95 (0.80-1.13)<br>1.01 (0.86-1.20)<br>0.92 (0.77-1.09)<br>0.89 (0.68-1.16)	Age, time period, BMI, cigarette smoking status and cigarettes per day, physical exercise, parental history of MI <60 years, multivitamin use, vitamin E supplement use, aspirin use, total energy, cereal fiber, alcohol, transfat, fruit and vegetables, legumes, eggs, low-fat dairy, high-fat dairy, fish, poultry, unprocessed red meat, processed red meat
Bernstein AM et al, 2012, USA	Nurses' Health Study	1980-2006, 26 years follow-up	84010 women, age 34-59 years: 2633 stroke cases	Validated FFQ, 61/131 food items	Nuts	0.00 serv/d<br>0.04<br>0.07<br>0.12<br>0.34<br>Per 1 serv/d	1.00<br>0.94 (0.83-1.06)<br>0.91 (0.80-1.04)<br>0.97 (0.85-1.10)<br>0.86 (0.75-0.98)<br>0.71 (0.51-1.00)	Age, time period, BMI, cigarette smoking status and cigarettes per day, physical exercise, parental history of MI <60 years, multivitamin use, vitamin E supplement use, aspirin use, total energy, cereal fiber, alcohol, transfat, fruit and vegetables, legumes, eggs, low-fat dairy, high-fat dairy, fish, poultry, unprocessed red meat, processed red meat, menopausal status, HRT use
Bernstein AM et al, 2012, USA	Health Professionals Follow-up Study & Nurses' Health Study	1986-2008, 22 years follow-up<br>1980-2006, 26 years follow-up	43150 men, age 40-75 years: 218 hemorrhagic stroke cases<br>829 ischemic stroke cases<br>84010 women, age 34-59 years: 475 hemorrhagic stroke cases<br>1383 ischemic stroke cases	Validated FFQ, 131 food items<br>Validated FFQ, 61/131 food items	Nuts (men/women), hemorrhagic stroke<br><br><br><br><br><br>Nuts (men/women), ischemic stroke	0.00/0.00 serv/d<br>0.07/0.04<br>0.14/0.07<br>0.25/0.12<br>0.60/0.34<br>Per 1 serv/d<br>0.00/0.00 serv/d<br>0.07/0.04<br>0.14/0.07<br>0.25/0.12<br>0.60/0.34<br>Per 1 serv/d	1.00<br>1.04 (0.76-1.43)<br>0.81 (0.57-1.13)<br>0.86 (0.62-1.20)<br>0.83 (0.69-1.16)<br>0.66 (0.34-1.26)<br>1.00<br>0.97 (0.84-1.11)<br>1.00 (0.86-1.15)<br>1.03 (0.89-1.18)<br>0.97 (0.84-1.12)<br>0.93 (0.70-1.22)	Age, time period, BMI, cigarette smoking status and cigarettes per day, physical exercise, parental history of MI <60 years, multivitamin use, vitamin E supplement use, aspirin use, total energy, cereal fiber, alcohol, transfat, fruit and vegetables, legumes, eggs, low-fat dairy, high-fat dairy, fish, poultry, unprocessed red meat, processed red meat, women: menopausal status, HRT use
Bao Y et al, 2013, USA	Nurses' Health Study	1980-2010, 30 years follow-up	76464 women, age 34-59 years: 878 stroke deaths	Validated FFQ, 61-116 food items	Nuts<br><br><br><br><br>Peanuts	Never<br><1/wk<br>1<br>2-4<br>≥5<br>Never	1.00<br>0.88 (0.74-1.05)<br>0.90 (0.72-1.13)<br>0.98 (0.77-1.24)<br>1.05 (0.73-1.52)<br>1.00	Age, race, BMI, physical activity, smoking, screening, multivitamin use, aspirin use, FH – DM, MI or cancer, history of DM, hypertension, or hypercholesterolemia, total energy, alcohol, red or processed meat, fruits,

					 <br><br>Tree nuts	<1/wk<br>1<br>≥2<br>Never<br><1/wk<br>1<br>≥2	0.92 (0.76-1.10)<br>0.94 (0.70-1.26)<br>1.18 (0.88-1.59)<br>1.00<br>0.87 (0.73-1.04)<br>0.84 (0.60-1.18)<br>1.09 (0.77-1.53)	vegetables, menopausal status, HRT
Bao Y et al, 2013, USA	Health Professional's Follow-up Study	1986-2010, 24 years follow-up	42498 men, age 45-70 years: 687 stroke deaths	Validated FFQ, 131 food items	Nuts<br><br><br><br><br>Peanuts<br><br><br><br>Tree nuts	Never<br><1/wk<br>1<br>2-4<br>≥5<br>Never<br><1/wk<br>1<br>≥2<br>Never<br><1/wk<br>1<br>≥2	1.00<br>0.74 (0.58-0.94)<br>0.97 (0.77-1.24)<br>0.88 (0.69-1.12)<br>0.78 (0.58-1.06)<br>1.00<br>0.89 (0.73-1.08)<br>0.82 (0.64-1.07)<br>0.81 (0.65-1.03)<br>1.00<br>1.02 (0.85-1.23)<br>1.11 (0.86-1.42)<br>0.90 (0.69-1.17)	Age, race, BMI, physical activity, smoking, screening, multivitamin use, aspirin use, FH – DM, MI or cancer, history of DM, hypertension, or hypercholesterolemia, total energy, alcohol, red or processed meat, fruits, vegetables
Di Giuseppe R et al, 2014, Germany	European Prospective Investigation into Cancer and Nutrition – Potsdam Study	1994-1998 – 2008, 8.3 years follow-up	26285 men and women, mean age 49.2/52.5 years: 288 stroke cases	Validated FFQ, 146 food items	Nuts, total stroke<br><br><br><br>Nuts, ischemic stroke<br><br><br><br>Nuts, hemorrhagic stroke<br><br><br>Nuts, fatal stroke	0 g/d<br>0.82<br>4.11<br>14.2<br>0 g/d<br>0.82<br>4.11<br>14.2<br>0 g/d<br>0.82<br>4.11<br>14.2<br>0 g/d<br>0.82<br>4.11<br>14.2	1.56 (1.17-2.08)<br>1.00<br>1.06 (0.75-1.52)<br>1.37 (0.92-2.05)<br>1.50 (1.09-2.07)<br>1.00<br>1.16 (0.79-1.72)<br>1.62 (1.05-2.49)<br>1.48 (1.09-2.01)<br>1.00<br>1.04 (0.71-1.53)<br>1.47 (0.97-2.24)<br>2.18 (1.00-4.78)<br>1.00<br>1.22 (0.47-3.18)<br>0.67 (0.15-2.97)	Age, sex, BMI, waist circumference, hypertension, hyperlipidemia, DM, smoking status, education, sport activity, alcohol, red meat, whole-grain breads, fruit, vegetables, fish, cakes and cookies, confectionary, fried potatoes, other beverages, total energy
Hshieh TT et al, 2015, USA	Physicians' Health Study	1999-2002 – NA, 9.6 years	20742 men, mean age 66 years: 142 stroke deaths	FFQ, 19 food items (validated in	Nuts	<1 serv/mo<br>1-3<br>1 serv/wk	1.00<br>0.91 (0.60-1.39)<br>0.82 (0.50-1.36)	Age, BMI, alcohol, smoking, exercise, calories, SFA, fruit and vegetables, red meat, prevalent DM, hypertension

		follow-up		other cohorts)		2-4<br>≥5	0.84 (0.48-1.47)<br>0.64 (0.32-1.30)	
Luu HN et al, 2015, USA	Southern Community Cohort Study	2002-2009 - NA, 5.4 years follow-up	71764 men and women, age 40-79 years: 217 stroke deaths	FFQ, 89 food items	Total nuts and peanut butter, ischemic stroke, African Americans<br><br><br><br><br>Total nuts and peanut butter, ischemic stroke, Caucasians<br><br><br><br><br>Total nuts and peanut butter, hemorrhagic stroke, African Americans<br><br><br><br><br>Total nuts and peanut butter, hemorrhagic stroke, Caucasian	<0.95 g/d<br>0.95-<3.08<br>3.08-<7.30<br>7.30-<18.45<br>≥18.45<br><0.95 g/d<br>0.95-<3.08<br>3.08-<7.30<br>7.30-<18.45<br>≥18.45<br><0.95 g/d<br>0.95-<3.08<br>3.08-<7.30<br>7.30-<18.45<br>≥18.45<br><0.95 g/d<br>0.95-<3.08<br>3.08-<7.30<br>7.30-<18.45<br>≥18.45	1.00<br>0.89 (0.49-1.62)<br>0.72 (0.35-1.46)<br>0.85 (0.44-1.64)<br>0.89 (0.45-1.74)<br>1.00<br>0.39 (0.10-1.55)<br>0.38 (0.10-1.46)<br>0.43 (0.12-1.54)<br>0.47 (0.12-1.76)<br>1.00<br>0.81 (0.37-1.75)<br>0.93 (0.43-2.02)<br>0.80 (0.36-1.74)<br>1.37 (0.67-2.80)<br>1.00<br>1.44 (0.36-5.68)<br>0.29 (0.05-1.72)<br>0.74 (0.17-3.23)<br>0.62 (0.12-3.26)	Age, sex, race, education, occupation, household income, marital status, smoking pack-years, alcohol, BMI, physical activity, vitamin supplement use, Charlson Comorbidity Index, metabolic conditions (hypertension, heart disease, DM, obesity, hypercholesterolemia), total energy, red meat, chicken and duck intake, seafood, vegetables, fruits
Luu HN et al, 2015, China	Shanghai Men's Health Study	2002-2006 - NA, 6.5 years follow-up	61123 men, age 40-74 years: 234/245 ischemic/ hemorrhagic stroke deaths	Validated FFQ, 84 food items	Peanuts, ischemic stroke<br><br><br><br><br>Peanuts, hemorrhagic stroke	<0.14 g/d<br>0.14-<0.72<br>0.72-<1.45<br>1.45-<2.54<br>≥2.54<br><0.14 g/d<br>0.14-<0.72<br>0.72-<1.45<br>1.45-<2.54<br>≥2.54	1.00<br>1.22 (0.79-1.90)<br>0.72 (0.47-1.10)<br>0.58 (0.39-0.86)<br>0.79 (0.54-1.14)<br>1.00<br>1.38 (0.90-2.12)<br>0.85 (0.56-1.27)<br>0.74 (0.51-1.07)<br>0.80 (0.55-1.16)	Age, race, education, occupation, household income, marital status, smoking pack-years, alcohol, BMI, physical activity, vitamin supplement use, Charlson Comorbidity Index, metabolic conditions (hypertension, heart disease, DM, obesity, dyslipidemia), total energy, red meat, chicken and duck intake, seafood, vegetables, fruits
Luu HN et al, 2015, China	Shanghai Women's Health Study	1996-2000 – NA, 12.2 years follow-up	73142 women, age 40-70 years: 354/352 ischemic/ hemorrhagic stroke deaths	Validated FFQ, 87 food items	Peanuts, ischemic stroke<br><br><br><br><br>Peanuts, hemorrhagic stroke	<0.14 g/d<br>0.14-<0.72<br>0.72-<1.45<br>1.45-<2.54<br>≥2.54<br><0.14 g/d<br>0.14-<0.72	1.00<br>0.80 (0.58-1.10)<br>0.84 (0.62-1.14)<br>0.71 (0.52-0.97)<br>0.72 (0.51-1.03)<br>1.00<br>0.74 (0.54-1.01)	Age, race, education, occupation, household income, marital status, smoking pack-years, alcohol, BMI, physical activity, vitamin supplement use, Charlson Comorbidity Index, metabolic conditions (hypertension, heart disease, DM, obesity,

						0.72-<1.45<br>1.45-<2.54<br>≥2.54	0.63 (0.44-0.86)<br>0.66 (0.49-0.91)<br>0.77 (0.55-1.07)	dyslipidemia), total energy, red meat, chicken and duck intake, seafood, vegetables, fruits
van den Brandt PA et al, 2015, Netherlands	Netherlands Cohort Study	1986-1996, 10 years follow-up	3202 subcohort members, men and women, age 55-69 years: 565 stroke deaths	Validated FFQ, 150 food items	Total nuts, all<br><br><br><br>Peanuts<br><br><br>Tree nuts<br><br><br>Peanut butter	0 g/d<br>0.1-<5<br>5-<10<br>≥10<br>0 g/d<br>0.1-<5<br>≥5<br>0 g/d<br>0.1-<5<br>≥5<br>0 g/d<br>0.1-<5<br>≥5	1.00<br>0.80 (0.63-1.01)<br>0.68 (0.48-0.97)<br>0.76 (0.56-1.02)<br>1.00<br>0.79 (0.62-0.99)<br>0.71 (0.54-0.94)<br>1.00<br>0.90 (0.70-1.15)<br>0.74 (0.44-1.24)<br>1.00<br>0.84 (0.64-1.11)<br>0.86 (0.60-1.23)	Age, sex, cigarette smoking, number of cigarettes per day, years of smoking, hypertension, DM, body height, BMI, non-occupational physical activity, highest level of education, alcohol, vegetables, fruits, energy, nutritional supplement use, women: HRT
Haring B et al, 2015, USA	Atherosclerosis Risk in Communities Study (ARIC)	1987-1989 - 2006, 22.7 years follow-up	11601 men and women, age 45-64 years: 699 stroke cases	FFQ, 66 food items	Nuts and peanut butter	0.00 serv/d<br>0.07<br>0.21<br>0.43<br>1.00	1.00<br>0.83 (0.65-1.05)<br>1.03 (0.81-1.32)<br>1.04 (0.81-1.33)<br>1.00 (0.77-1.31)	Age, sex, race, study center, total energy intake, smoking, cigarette years, education, SBP, use of antihypertensive medication, HDL-cholesterol, total cholesterol, use of lipid lowering medication, BMI, WHR, alcohol, sports-related physical activity, leisure-time physical activity, carbohydrates, fiber, fat, magnesium
Bonaccio M et al, 2015, Italy	The Moli-sani Study	2005-2010 – 2011, 4.3 years follow-up	19386 men and women, mean age 54.5 years: 19 stroke deaths	Validated FFQ, 188 food items	Nuts	Never<br>Ever	1.00<br>0.98 (0.36-2.66)	Age, sex, education, smoking status, leisure-time physical activity, BMI, energy intake, Mediterranean diet score without nuts
Gopinath B et al, 2015, Australia	The Blue Mountains Eye Study	1992-1994 - 2007, 15 years follow-up	2893 men and women, age ≥49 years: 430 stroke deaths	Validated FFQ, 145 food items	Nuts, all<br><br><br>Nuts, women<br><br><br>Nuts, men	0-0.50 g/d<br>0.90-4.55<br>4.90-100<br>0-0.50 g/d<br>0.90-4.55<br>4.90-100<br>0-0.50 g/d<br>0.90-4.55<br>4.90-100	1.00<br>0.70 (0.47-1.02)<br>0.88 (0.60-1.29)<br>1.00<br>0.52 (0.30-0.88)<br>0.67 (0.40-1.14)<br>1.00<br>0.86 (0.46-1.59)<br>1.30 (0.75-2.28)	Age, sex, qualifications, total diet score, BMI, current smoking status, alcohol, self-rated health, walking disability, hypertension, diabetes, doctor-diagnosed history of cancer, angina, stroke, acute myocardial infarction

Wang JB et al, 2016, China	Linxian Nutrition Intervention Trial cohort	1984-1991 - 2010, 19-26 years follow-up	2445 men and women, age 40-69 years: 452 stroke deaths	FFQ, 64 food items	Nuts	Per 3 times/mo	0.99 (0.93-1.05)	Age, sex, commune, smoking, drinking, season, BMI

BMI=body mass index, DM=diabetes mellitus, FFQ=food frequency questionnaire, FH=family history, HRT=hormone replacement therapy, MI=myocardial infarction, NA=not available, SFA=saturated fatty acids, WHR=waist-to-hip ratio

Supplementary table 5. Nut consumption and cardiovascular disease

Author, publication year, country	Study name	Study period	Number of participants, gender, age, number of cases/deaths	Dietary assessment	Exposure and subgroup	Nut consumption frequency or amount	Relative risks (95% confidence intervals)	Adjustment for confounding factors
Blomhoff R et al, 2006, USA	Iowa Women's Health Study	1986-2001, 15 years follow-up	31778 women, age 55-69 years: 1675 CVD deaths	FFQ, 127 food items (validated in other studies)	Nuts and peanut butter	0.0/wk<br>0.5<br>1.5<br>7.0	1.00<br>1.00 (0.86-1.17)<br>0.84 (0.73-0.96)<br>0.72 (0.60-0.88)	Age, energy intake, BMI, WHR, physical activity, HRT, multivitamin supplements, alcohol, whole grain, refined grain, red meat, fish and seafood, total fruits and vegetables
Fitzgerald KC et al, 2012, USA	Women's Health Study	1992-1994 - NA, 14.6 years follow-up	34827 women, age ≥45 years: 1094 CVD cases	Validated FFQ, 133 food items	Nuts	<0.13 serv/d<br>0.13-0.20<br>0.21-0.34<br>0.35-0.57<br>≥0.58	1.00<br>0.93 (0.75-1.15)<br>0.99 (0.83-1.17)<br>0.94 (0.79-1.13)<br>1.02 (0.85-1.23)	Age, randomization status, smoking, postmenopausal status, HRT, alcohol intake, energy, physical activity, cigarettes per day, highest education level
Von Ruesten A et al, 2013, Germany	European Prospective Investigation into Cancer and Nutrition–Potsdam study	1994/1998–NA,<br>8 years follow-up	23,531 men and women, age 35-65 years: 363 CVD cases	Validated FFQ,<br>148 food items	Nuts	Per 5 g/d	1.00 (0.92-1.08)	Age, sex, smoking status, pack-years of smoking, alcohol, leisure-time physical activity, BMI, WHR, prevalent hypertension, high blood lipid levels, education, vitamin supplementation, total energy, non-consumption of the food group, other food groups
Bao Y et al, 2013, USA	Nurses' Health Study	1980-2010, 30 years follow-up	76464 women, age 34-59 years: 3086 CVD deaths	Validated FFQ, 61-116 food items	Nuts	Never<br><1/wk<br>1<br>2-4<br>≥5	1.00<br>0.85 (0.78-0.93)<br>0.82 (0.72-0.92)<br>0.82 (0.72-0.93)<br>0.82 (0.66-1.01)	Age, race, BMI, physical activity, smoking, screening, multivitamin use, aspirin use, FH – DM, MI or cancer, history of DM, hypertension, or hypercholesterolemia, total energy, alcohol, red or processed meat, fruits, vegetables, menopausal status, HRT
Bao Y et al, 2013, USA	Health Professional's Follow-up Study	1986-2010, 24 years follow-up	42498 men, age 45-70 years: 3385 CVD deaths	Validated FFQ, 131 food items	Nuts	Never<br><1/wk<br>1<br>2-4<br>≥5	1.00<br>0.82 (0.74-0.91)<br>0.83 (0.75-0.93)<br>0.77 (0.69-0.86)<br>0.73 (0.64-0.83)	Age, race, BMI, physical activity, smoking, screening, multivitamin use, aspirin use, FH – DM, MI or cancer, history of DM, hypertension, or hypercholesterolemia, total energy, alcohol, red or processed meat, fruits, vegetables

Guasch-Ferre M et al, 2013, Spain	PREDIMED Study	NA-NA, 4.8 years follow-up	7216 men and women, age 55-80 years: 81 CVD deaths	Validated FFQ, 137 food items	Nuts<br><br><br>Walnuts<br><br><br>Other nuts (excluding walnuts)	Never<br>1-3 serv/wk<br>>3<br>Never<br>1-3 serv/wk<br>>3<br>Never<br>1-3 serv/wk<br>>3	1.00<br>0.42 (0.24-0.74)<br>0.45 (0.25-0.81)<br>1.00<br>0.41 (0.23-0.73)<br>0.53 (0.29-0.98)<br>1.00<br>0.74 (0.45-1.23)<br>0.42 (0.20-0.89)	Age, sex, and intervention group, BMI, smoking status, education, leisure time physical activity, DM, hypercholesterolemia, oral antidiabetic medication, antihypertensive medication, use of statins, total energy, vegetables, fruits, red meat, eggs, fish, alcohol, Mediterranean diet adherence
Chiuve SE et al, 2014, USA	Nurses' Health Study	1986-2010, 24 years follow-up	40680 women, age 40-65 years: 2525 CVD events	Validated FFQ, 131 food items	Nuts	0 serv/wk<br>0.1-1<br>>1	1.00<br>0.89 (0.80-0.99)<br>0.80 (0.67-0.95)	Age, smoking status, BMI, alcohol, exercise, fruits, vegetables, sugar-sweetened beverages, red and processed meats, cereal fiber
Chiuve SE et al, 2014, USA	Health Professionals Follow-up Study	1986-2010, 24 years follow-up	23026 men, age 40-75 years: 2375 CVD events	Validated FFQ, 131 food items	Nuts	0 serv/wk<br>0.1-1<br>>1	1.00<br>1.06 (0.94-1.20)<br>0.88 (0.74-1.04)	Age, smoking status, BMI, alcohol, exercise, fruits, vegetables, sugar-sweetened beverages, red and processed meats, cereal fiber
Hshieh TT et al, 2015, USA	Physicians' Health Study	1999-2002 – NA, 9.6 years follow-up	20742 men, mean age 66 years: 760 CVD deaths	FFQ, 19 food items (validated in other cohorts)	Nuts	<1 serv/mo<br>1-3<br>1 serv/wk<br>2-4<br>≥5	1.00<br>0.98 (0.82-1.17)<br>0.89 (0.72-1.11)<br>0.80 (0.62-1.03)<br>0.74 (0.55-1.02)	Age, BMI, alcohol, smoking, exercise, calories, SFA, fruit and vegetables, red meat, prevalent DM, hypertension
Luu HN et al, 2015, USA	Southern Community Cohort Study	2002-2009 - NA, 5.4 years follow-up	71764 men and women, age 40-79 years: 1857 CVD deaths	FFQ, 89 food items	Total nuts and peanut butter, African Americans<br><br><br><br>Total nuts and peanut butter, Caucasians	<0.95 g/d<br>0.95-<3.08<br>3.08-<7.30<br>7.30-<18.45<br>≥18.45<br><0.95 g/d<br>0.95-<3.08<br>3.08-<7.30<br>7.30-<18.45<br>≥18.45	1.00<br>0.85 (0.72-1.00)<br>0.82 (0.68-0.99)<br>0.81 (0.68-0.97)<br>0.77 (0.63-0.92)<br>1.00<br>0.80 (0.61-1.06)<br>0.74 (0.56-0.97)<br>0.66 (0.49-0.87)<br>0.62 (0.46-0.84)	Age, sex, race, education, occupation, household income, marital status, smoking pack-years, alcohol, BMI, physical activity, vitamin supplement use, Charlson Comorbidity Index, metabolic conditions (hypertension, heart disease, DM, obesity, hypercholesterolemia), total energy, red meat, chicken and duck intake, seafood, vegetables, fruits
Luu HN et al, 2015, China	Shanghai Men's Health Study	2002-2006 - NA, 6.5 years follow-up	61123 men, age 40-74 years: 1108 CVD deaths	Validated FFQ, 84 food items	Peanuts	<0.14 g/d<br>0.14-<0.72<br>0.72-<1.45<br>1.45-<2.54<br>≥2.54	1.00<br>0.94 (0.75-1.17)<br>0.70 (0.57-0.85)<br>0.66 (0.56-0.79)<br>0.78 (0.66-0.93)	Age, race, education, occupation, household income, marital status, smoking pack-years, alcohol, BMI, physical activity, vitamin supplement use, Charlson Comorbidity Index,

								metabolic conditions (hypertension, heart disease, DM, obesity, dyslipidemia), total energy, red meat, chicken and duck intake, seafood, vegetables, fruits
Luu HN et al, 2015, China	Shanghai Women's Health Study	1996-2000 – NA, 12.2 years follow-up	73142 women, age 40-70 years: 1479 CVD deaths	Validated FFQ, 87 food items	Peanuts	<0.14 g/d<br>0.14-<0.72<br>0.72-<1.45<br>1.45-<2.54<br>≥2.54	1.00<br>0.79 (0.67-0.92)<br>0.78 (0.67-0.91)<br>0.71 (0.61-0.83)<br>0.72 (0.61-0.86)	Age, race, education, occupation, household income, marital status, smoking pack-years, alcohol, BMI, physical activity, vitamin supplement use, Charlson Comorbidity Index, metabolic conditions (hypertension, heart disease, DM, obesity, dyslipidemia), total energy, red meat, chicken and duck intake, seafood, vegetables, fruits
van den Brandt PA et al, 2015, Netherlands	Netherlands Cohort Study	1986-1996, 10 years follow-up	3202 subcohort members, men and women, age 55-69 years: 2985 CVD deaths	Validated FFQ, 150 food items	Total nuts, all<br><br><br><br>Peanuts<br><br><br>Tree nuts<br><br><br>Peanut butter	0 g/d<br>0.1-<5<br>5-<10<br>≥10<br>0 g/d<br>0.1-<5<br>≥5<br>0 g/d<br>0.1-<5<br>≥5<br>0 g/d<br>0.1-<5<br>≥5	1.00<br>0.89 (0.76-1.03)<br>0.74 (0.59-0.91)<br>0.83 (0.69-1.00)<br>1.00<br>0.86 (0.74-0.99)<br>0.78 (0.66-0.93)<br>1.00<br>0.91 (0.78-1.07)<br>0.91 (0.68-1.23)<br>1.00<br>1.00 (0.85-1.17)<br>0.99 (0.80-1.23)	Age, sex, cigarette smoking, number of cigarettes per day, years of smoking, hypertension, DM, body height, BMI, non-occupational physical activity, highest level of education, alcohol, vegetables, fruits, energy, nutritional supplement use, women: HRT
Bonaccio M et al, 2015, Italy	The Moli-sani Study	2005-2010 – 2011, 4.3 years follow-up	19386 men and women, mean age 54.5 years: 104 CVD deaths	Validated FFQ, 188 food items	Nuts	Never<br>Ever	1.00<br>0.87 (0.57-1.32)	Age, sex, education, smoking status, leisure-time physical activity, BMI, energy intake, Mediterranean diet score without nuts
Gopinath B et al, 2015, Australia	The Blue Mountains Eye Study	1992-1994 - 2007, 15 years follow-up	2893 men and women, age ≥49 years: 546 CVD deaths	Validated FFQ, 145 food items	Nuts, all<br><br><br>Nuts, women<br><br><br>Nuts, men	0-0.50 g/d<br>0.90-4.55<br>4.90-100<br>0-0.50 g/d<br>0.90-4.55<br>4.90-100<br>0-0.50 g/d	1.00<br>0.76 (0.61-0.94)<br>0.90 (0.73-1.12)<br>1.00<br>0.61 (0.44-0.85)<br>0.81 (0.59-1.13)<br>1.00	Age, sex, qualifications, total diet score, BMI, current smoking status, alcohol, self-rated health, walking disability, hypertension, diabetes, doctor-diagnosed history of cancer, angina, stroke, acute myocardial infarction

						0.90-4.55<br>4.90-100	0.86 (0.64-1.15)<br>1.00 (0.75-1.34)	
Wang JB et al, 2016, China	Linxian Nutrition Intervention Trial cohort	1984-1991 - 2010, 19-26 years follow-up	2445 men and women, age 40-69 years: 807 CVD deaths	FFQ, 64 food items	Nuts	Per 3 times/mo	0.96 (0.91-1.01)	Age, sex, commune, smoking, drinking, season, BMI
Eslamparast T et al, 2016, Iran	Golestan Cohort Study	2004 - 2013, 7 years follow-up	50045 men and women, age ≥40 years: 2016 CVD deaths	Validated FFQ	Nuts, all<br><br><br><br>Nuts, women<br><br><br><br>Nuts, men	Never<br><1 serv/wk<br>1-<3<br>≥3<br>Never<br><1 serv/wk<br>1-<3<br>≥3<br>Never<br><1 serv/wk<br>1-<3<br>≥3	1.00<br>0.87 (0.79-0.97)<br>0.75 (0.63-0.89)<br>0.77 (0.58-1.01)<br>1.00<br>0.83 (0.71-0.96)<br>0.65 (0.49-0.86)<br>0.55 (0.33-0.91)<br>1.00<br>0.92 (0.80-1.05)<br>0.83 (0.66-1.03)<br>0.90 (0.66-1.26)	Age, sex, BMI, education, place of residence, smoking status, opium, alcohol, physical activity, wealth score, diabetes, hypertension, total energy, fish, red meat, chicken, fruits, vegetables, dairy products, eggs, total fiber, magnesium, zinc, copper

BMI=body mass index, CVD=cardiovascular disease, DM=diabetes mellitus, FFQ=food frequency questionnaire, FH=family history, HRT=hormone replacement therapy, MI=myocardial infarction, NA=not available, SFA=saturated fatty acids, WHR=waist-to-hip ratio

Supplementary table 6. Nut consumption and total cancer

Author, publication year, country	Study name	Study period	Number of participants, gender, age, number of cases/deaths	Dietary assessment	Exposure and subgroup	Nut consumption frequency or amount	Relative risks (95% confidence intervals)	Adjustment for confounding factors
Von Ruesten A et al, 2013, Germany	European Prospective Investigation into Cancer and Nutrition–Potsdam study	1994/1998–NA, 8 years follow-up	23,531 men and women, age 35–65 years: 844 cancer cases	Validated FFQ, 148 food items	Nuts	Per 5 g/d	1.01 (0.96-1.05)	Age, sex, smoking status, pack-years of smoking, alcohol, leisure-time physical activity, BMI, WHR, prevalent hypertension, high blood lipid levels, education, vitamin supplementation, total energy, non-consumption of the food group, other food groups
Bao Y et al, 2013, USA	Nurses' Health Study	1980-2010, 30 years follow-up	76464 women, age 34-59 years: 6535 cancer deaths	Validated FFQ, 61-116 food items	Nuts	Never<br><1/wk<br>1<br>2-4<br>≥5	1.00<br>0.94 (0.88-1.01)<br>0.91 (0.84-0.99)<br>0.89 (0.82-0.98)<br>0.94 (0.82-1.09)	Age, race, BMI, physical activity, smoking, screening, multivitamin use, aspirin use, FH – DM, MI or cancer, history of DM, hypertension, or hypercholesterolemia, total energy, alcohol, red or processed meat, fruits, vegetables, menopausal status, HRT
					Peanuts	Never<br><1/wk<br>1<br>≥2	1.00<br>0.94 (0.88-1.00)<br>0.93 (0.83-1.04)<br>0.97 (0.86-1.09)	
					Tree nuts	Never<br><1/wk<br>1<br>≥2	1.00<br>0.95 (0.89-1.01)<br>0.96 (0.85-1.09)<br>0.83 (0.72-0.96)	

Bao Y et al, 2013, USA	Health Professional's Follow-up Study	1986-2010, 24 years follow-up	42498 men, age 45-70 years: 3758 cancer deaths	Validated FFQ, 131 food items	Nuts<br><br><br><br><br>Peanuts<br><br><br><br>Tree nuts	Never<br><1/wk<br>1<br>2-4<br>≥5<br>Never<br><1/wk<br>1<br>≥2<br>Never<br><1/wk<br>1<br>≥2	1.00<br>0.91 (0.82-1.01)<br>0.98 (0.88-1.09)<br>0.95 (0.85-1.05)<br>0.86 (0.75-0.98)<br>1.00<br>0.91 (0.83-0.99)<br>1.00 (0.89-1.11)<br>0.93 (0.84-1.03)<br>1.00<br>0.91 (0.84-0.98)<br>0.95 (0.85-1.06)<br>0.82 (0.73-0.92)	Age, race, BMI, physical activity, smoking, screening, multivitamin use, aspirin use, FH – DM, MI or cancer, history of DM, hypertension, or hypercholesterolemia, total energy, alcohol, red or processed meat, fruits, vegetables
Guasch-Ferre M et al, 2013, Spain	PREDIMED Study	NA-NA, 4.8 years follow-up	7216 men and women, age 55-80 years: 130 cancer deaths	Validated FFQ, 137 food items	Nuts<br><br><br>Walnuts<br><br><br>Other nuts (excluding walnuts)	Never<br>1-3 serv/wk<br>>3<br>Never<br>1-3 serv/wk<br>>3<br>Never<br>1-3 serv/wk<br>>3	1.00<br>0.79 (0.52-1.20)<br>0.60 (0.37-0.98)<br>1.00<br>0.76 (0.51-1.12)<br>0.46 (0.27-0.79)<br>1.00<br>0.79 (0.53-1.18)<br>0.75 (0.44-1.27)	Age, sex, and intervention group, BMI, smoking status, education, leisure time physical activity, DM, hypercholesterolemia, oral antidiabetic medication, antihypertensive medication, use of statins, total energy, vegetables, fruits, red meat, eggs, fish, alcohol, Mediterranean diet adherence
Hshieh TT et al, 2015, USA	Physicians' Health Study	1999-2002 – NA, 9.6 years follow-up	20742 men, mean age 66 years: 868 cancer deaths	FFQ, 19 food items (validated in other cohorts)	Nuts	<1 serv/mo<br>1-3<br>1 serv/wk<br>2-4<br>≥5	1.00<br>0.91 (0.77-1.08)<br>0.88 (0.72-1.07)<br>0.87 (0.68-1.09)<br>0.87 (0.66-1.15)	Age, BMI, alcohol, smoking, exercise, calories, SFA, fruit and vegetables, red meat, prevalent DM, hypertension
Luu HN et al, 2015, USA	Southern Community Cohort Study	2002-2009 - NA, 5.4 years follow-up	71764 men and women, age 40-79 years: 1551 cancer deaths	FFQ, 89 food items	Total nuts and peanut butter, African Americans<br><br><br><br><br>Total nuts and peanut butter, Caucasians	<0.95 g/d<br>0.95-<3.08<br>3.08-<7.30<br>7.30-<18.45<br>≥18.45<br><0.95 g/d<br>0.95-<3.08<br>3.08-<7.30<br>7.30-<18.45<br>≥18.45	1.00<br>0.89 (0.73-1.07)<br>0.85 (0.69-1.05)<br>0.91 (0.74-1.11)<br>0.74 (0.60-0.92)<br>1.00<br>0.98 (0.72-1.32)<br>0.89 (0.65-1.21)<br>0.68 (0.48-0.94)<br>0.93 (0.68-1.29)	Age, sex, race, education, occupation, household income, marital status, smoking pack-years, alcohol, BMI, physical activity, vitamin supplement use, Charlson Comorbidity Index, metabolic conditions (hypertension, heart disease, DM, obesity, hypercholesterolemia), total energy, red meat, chicken and duck intake, seafood, vegetables, fruits

Luu HN et al, 2015, China	Shanghai Men's Health Study	2002-2006 - NA, 6.5 years follow-up	61123 men, age 40-74 years: 1492 cancer deaths	Validated FFQ, 84 food items	Peanuts	<0.14 g/d<br>0.14-<0.72<br>0.72-<1.45<br>1.45-<2.54<br>≥2.54	1.00<br>0.91 (0.72-1.13)<br>0.88 (0.74-1.05)<br>1.01 (0.88-1.17)<br>0.96 (0.83-1.11)	Age, race, education, occupation, household income, marital status, smoking pack-years, alcohol, BMI, physical activity, vitamin supplement use, Charlson Comorbidity Index, metabolic conditions (hypertension, heart disease, DM, obesity, dyslipidemia), total energy, red meat, chicken and duck intake, seafood, vegetables, fruits
Luu HN et al, 2015, China	Shanghai Women's Health Study	1996-2000 – NA, 12.2 years follow-up	73142 women, age 40-70 years: 2040 cancer deaths	Validated FFQ, 87 food items	Peanuts	<0.14 g/d<br>0.14-<0.72<br>0.72-<1.45<br>1.45-<2.54<br>≥2.54	1.00<br>0.87 (0.76-1.00)<br>0.83 (0.61-1.06)<br>0.92 (0.81-1.06)<br>0.97 (0.84-1.12)	Age, race, education, occupation, household income, marital status, smoking pack-years, alcohol, BMI, physical activity, vitamin supplement use, Charlson Comorbidity Index, metabolic conditions (hypertension, heart disease, DM, obesity, dyslipidemia), total energy, red meat, chicken and duck intake, seafood, vegetables, fruits
van den Brandt PA et al, 2015, Netherlands	Netherlands Cohort Study	1986-1996, 10 years follow-up	3202 subcohort members, men and women, age 55-69 years: 3917 cancer deaths	Validated FFQ, 150 food items	Total nuts, all<br><br><br><br>Peanuts<br><br><br>Tree nuts<br><br><br>Peanut butter	0 g/d<br>0.1-<5<br>5-<10<br>≥10<br>0 g/d<br>0.1-<5<br>≥5<br>0 g/d<br>0.1-<5<br>≥5<br>0 g/d<br>0.1-<5<br>≥5	1.00<br>0.92 (0.81-1.05)<br>0.82 (0.68-0.98)<br>0.79 (0.67-0.93)<br>1.00<br>0.93 (0.82-1.05)<br>0.80 (0.69-0.93)<br>1.00<br>0.97 (0.85-1.11)<br>0.81 (0.62-1.05)<br>1.00<br>1.04 (0.90-1.20)<br>0.98 (0.82-1.17)	Age, sex, cigarette smoking, number of cigarettes per day, years of smoking, hypertension, DM, body height, BMI, non-occupational physical activity, highest level of education, alcohol, vegetables, fruits, energy, nutritional supplement use, women: HRT
Bonaccio M et al, 2015, Italy	The Moli-sani Study	2005-2010 – 2011, 4.3 years follow-up	19386 men and women, mean age 54.5 years: 124 cancer deaths	Validated FFQ, 188 food items	Nuts	Never<br>Ever	1.00<br>0.64 (0.44-0.94)	Age, sex, education, smoking status, leisure-time physical activity, BMI, energy intake, Mediterranean diet score without nuts

Eslamparast T et al, 2016, Iran	Golestan Cohort Study	2004 - 2013, 7 years follow-up	50045 men and women, age ≥40 years: 887 cancer deaths	Validated FFQ	Nuts, all<br><br><br><br>Nuts, women<br><br><br><br>Nuts, men	Never<br><1 serv/wk<br>1-<3<br>≥3<br>Never<br><1 serv/wk<br>1-<3<br>≥3<br>Never<br><1 serv/wk<br>1-<3<br>≥3	1.00<br>0.96 (0.82-1.11)<br>0.84 (0.65-1.07)<br>0.62 (0.38-1.01)<br>1.00<br>0.91 (0.73-1.14)<br>0.72 (0.48-1.07)<br>0.43 (0.18-1.01)<br>1.00<br>0.98 (0.80-1.20)<br>0.90 (0.65-1.25)<br>0.73 (0.41-1.33)	Age, sex, BMI, education, place of residence, smoking status, opium, alcohol, physical activity, wealth score, diabetes, hypertension, total energy, fish, red meat, chicken, fruits, vegetables, dairy products, eggs, total fiber, magnesium, zinc, copper

BMI=body mass index, DM=diabetes mellitus, FFQ=food frequency questionnaire, FH=family history, HRT=hormone replacement therapy, MI=myocardial infarction, NA=not available, SFA=saturated fatty acids, WHR=waist-to-hip ratio

Supplementary table 7. Nut consumption and all-cause mortality

Author, publication year, country, region	Study name	Study period	Number of participants, gender, age, number of deaths	Dietary assessment	Exposure and subgroup	Nut consumption frequency or amount	Relative risks (95% confidence intervals)	Adjustment for confounding factors
Fraser GE et al, 1997, USA	Adventist Health Study	1974-1976 – 1985, ~9.5 years follow-up	1668 black men and women, age ≥25 years: 416 deaths	FFQ, 65 food items	Nuts<br><br><br>Nuts, men<br><br><br>Nuts, women	<1/wk<br>1-4/wk<br>≥5/wk<br><1/wk<br>1-4/wk<br>≥5/wk<br><1/wk<br>1-4/wk<br>≥5/wk	1.0<br>0.6 (0.4-0.9)<br>0.6 (0.3-1.0)<br>1.0<br>0.7 (0.4-1.4)<br>0.6 (0.2-1.3)<br>1.0<br>0.4 (0.2-0.9)<br>0.5 (0.2-1.2)	Age, smoking, exercise
Fraser GE et al, 1997, USA	Adventist Health Study	1974-1976 – 1988, 12 years follow-up	603 white men and women, age >84 years at baseline + additional subjects who became >84 years during follow-up (number not available): 1387 deaths	FFQ, 65 food items	Nuts<br><br><br>Nuts, men<br><br><br>Nuts, women	<1/wk<br>1-4/wk<br>≥5/wk<br><1/wk<br>1-4/wk<br>≥5/wk<br><1/wk<br>1-4/wk<br>≥5/wk	1.00<br>0.88 (0.76-1.01)<br>0.82 (0.70-0.96)<br>1.00<br>0.84 (0.64-1.09)<br>0.77 (0.58-1.02)<br>1.00<br>0.89 (0.75-1.06)<br>0.84 (0.70-1.01)	Age, sex, DM, smoking, exercise, fruits, bread, donuts, sweet desserts, beef, fish
Mann JI, 1997, England	The Oxford Vegetarian Study	1980-1984 – 1995, 13.3 years follow-up	10802 men and women, age 16-79 years: 392 deaths	FFQ	Nuts	<1/wk<br>1-4<br>≥5	1.00<br>0.99 (0.79-1.25)<br>0.77 (0.58-1.01)	Age, sex, smoking, social class
Blomhoff R et al, 2006, USA	Iowa Women's Health Study	1986-2001, 15 years follow-up	31778 women, age 55-69 years: 5451 deaths	FFQ, 127 food items (validated in other studies)	Nuts and peanut butter	0.0/wk<br>0.5<br>1.5<br>7.0	1.00<br>0.93 (0.85-1.02)<br>0.88 (0.81-0.95)<br>0.89 (0.81-0.99)	Age, energy intake, BMI, WHR, physical activity, HRT, multivitamin supplements, alcohol, whole grain, refined grain, red meat, fish and seafood, total fruits and vegetables
Leenders M et al, 2013, Europe	European Prospective Investigation into Cancer and Nutrition	1992-2000 – 2010, 13 years follow-up	451151 men and women, age 25-70 years: 25682 deaths	Validated FFQ, 7-day record	Nuts and seeds	1<br>2<br>3<br>4	1.16 (1.11-1.21)<br>1.00<br>1.02 (0.97-1.06)<br>1.03 (0.99-1.08)	Age, smoking status, smoking duration, time since stopped smoking, number of cigarettes per day, alcohol, BMI, physical activity, education, processed meat

Bao Y et al, 2013, USA	Nurses' Health Study	1980-2010, 30 years follow-up	76464 women, age 34-59 years: 16200 deaths	Validated FFQ, 61-116 food items	Nuts<br><br><br><br><br><br>Peanuts<br><br><br><br>Tree nuts	Never<br><1/wk<br>1<br>2-4<br>5-6<br>≥7<br>Never<br><1/wk<br>1<br>≥2<br>Never<br><1/wk<br>1<br>≥2	1.00<br>0.94 (0.90-0.98)<br>0.88 (0.83-0.92)<br>0.85 (0.80-0.90)<br>0.88 (0.78-0.98)<br>0.79 (0.68-0.91)<br>1.00<br>0.92 (0.88-0.96)<br>0.88 (0.82-0.94)<br>0.91 (0.84-0.98)<br>1.00<br>0.95 (0.92-1.00)<br>0.90 (0.83-0.97)<br>0.82 (0.75-0.90)	Age, race, BMI, physical activity, smoking, screening, multivitamin use, aspirin use, FH – DM, MI or cancer, history of DM, hypertension, or hypercholesterolemia, total energy, alcohol, red or processed meat, fruits, vegetables, menopausal status, HRT
Bao Y et al, 2013, USA	Health Professionals Follow-up Study	1986-2010, 24 years follow-up	42498 men, age 45-70 years: 11229 deaths	Validated FFQ, 131 food items	Nuts<br><br><br><br><br><br>Peanuts<br><br><br><br>Tree nuts	Never<br><1/wk<br>1<br>2-4<br>5-6<br>≥7<br>Never<br><1/wk<br>1<br>≥2<br>Never<br><1/wk<br>1<br>≥2	1.00<br>0.91 (0.85-0.96)<br>0.91 (0.86-0.97)<br>0.89 (0.83-0.94)<br>0.83 (0.76-0.91)<br>0.80 (0.73-0.88)<br>1.00<br>0.91 (0.87–0.96)<br>0.93 (0.88–0.99)<br>0.86 (0.82–0.92)<br>1.00<br>0.94 (0.90–0.98)<br>0.95 (0.89–1.01)<br>0.84 (0.78–0.89)	Age, race, BMI, physical activity, smoking, screening, multivitamin use, aspirin use, FH – DM, MI or cancer, history of DM, hypertension, or hypercholesterolemia, total energy, alcohol, red or processed meat, fruits, vegetables
Guasch-Ferre M et al, 2013, Spain	PREDIMED Study	NA-NA, 4.8 years follow-up	7216 men and women, age 55-80 years: 323 deaths	Validated FFQ, 137 food items	Nuts<br><br><br>Walnuts<br><br><br>Other nuts (excluding walnuts)	Never<br>1-3 serv/wk<br>>3<br>Never<br>1-3 serv/wk<br>>3<br>Never<br>1-3 serv/wk<br>>3	1.00<br>0.71 (0.54- 0.93)<br>0.61 (0.45- 0.83)<br>1.00<br>0.66 (0.51-0.86)<br>0.55 (0.40-0.76)<br>1.00<br>0.80 (0.62-1.03)<br>0.66 (0.46-0.93)	Age, sex, and intervention group, BMI, smoking status, education, leisure time physical activity, DM, hypercholesterolemia, oral antidiabetic medication, antihypertensive medication, use of statins, total energy, vegetables, fruits, red meat, eggs, fish, alcohol, Mediterranean diet adherence
Fernandez-Montero A et al, 2014, Spain	The SUN Study	1999 – 2012, 5	17184 men and women, mean age	Validated FFQ, 136	Nuts	0 g/d<br>2.3	1.00<br>0.81 (0.44-1.50)	Age, sex, BMI, smoking, alcohol intake, adherence to Mediterranean

		years<br>follow-up	38-42 years: 119 deaths	food items	 <br><br>Nuts	4.3<br>6.4<br>20.1<br>Never/almost never<br>1-3/mo<br>1/wk<br>≥2/wk	0.99 (0.56-1.77)<br>0.82 (0.46-1.46)<br>0.56 (0.30-1.06)<br>1.00<br>0.82 (0.51-1.30)<br>0.81 (0.46-1.43)<br>0.44 (0.23-0.86)	diet, use of special diets, marital status, hypercholesterolemia, hypertension, physical activity, length of television watching, cancer, CVD, DM, total energy
Hshieh TT et al, 2015, USA	Physicians' Health Study	1999-2002 – NA, 9.6 years follow-up	20742 men, mean age 66 years: 2732 deaths	FFQ, 19 food items (validated in other cohorts)	Nuts	<1 serv/mo<br>1-3<br>1 serv/wk<br>2-4<br>≥5	1.00<br>0.92 (0.83-1.01)<br>0.84 (0.75-0.95)<br>0.86 (0.75-0.95)<br>0.76 (0.64-0.89)	Age, BMI, alcohol, smoking, exercise, calories, SFA, fruit and vegetables, red meat, prevalent DM, hypertension
Luu HN et al, 2015, USA	Southern Community Cohort Study	2002-2009, 5.4 years follow-up	71764 men and women, age 40-79 years: 6256 deaths	FFQ, 89 food items	Total nuts and peanut butter, all<br><br><br><br>Nuts<br><br><br><br><br>Peanut butter intake only<br><br><br><br>Total nuts and peanut butter, men<br><br><br><br>Nuts<br><br><br><br><br>Peanut butter intake	<0.95 g/d<br>0.95-<3.08<br>3.08-<7.30<br>7.30-<18.45<br>≥18.45<br><0.36 g/d<br>0.36-<0.66<br>0.66-<4.14<br>4.14-<8.63<br>≥8.63<br><0.19 g/d<br>0.19-<0.59<br>0.59-<2.18<br>2.18-<6.32<br>≥6.32<br><0.95 g/d<br>0.95-<3.08<br>3.08-<7.30<br>7.30-<18.45<br>≥18.45<br><0.36 g/d<br>0.36-<0.66<br>0.66-<4.14<br>4.14-<8.63<br>≥8.63<br><0.19 g/d	1.00<br>0.90 (0.83-0.97)<br>0.84 (0.77-0.91)<br>0.84 (0.77-0.91)<br>0.79 (0.73-0.86)<br>1.00<br>0.92 (0.85-0.99)<br>0.81 (0.74-0.88)<br>0.78 (0.72-0.84)<br>0.73 (0.67-0.79)<br>1.00<br>0.91 (0.83-0.99)<br>0.80 (0.72-0.91)<br>1.00 (0.91-1.09)<br>0.86 (0.79-0.94)<br>1.00<br>0.83 (0.74-0.94)<br>0.84 (0.74-0.95)<br>0.77 (0.68-0.87)<br>0.72 (0.64-0.81)<br>1.00<br>0.88 (0.80-0.98)<br>0.76 (0.66-0.87)<br>0.72 (0.64-0.81)<br>0.70 (0.62-0.79)<br>1.00	Age, sex, race, education, occupation, household income, marital status, smoking pack-years, alcohol, BMI, physical activity, vitamin supplement use, Charlson Comorbidity Index, metabolic conditions (hypertension, heart disease, DM, obesity, hypercholesterolemia), total energy, red meat, chicken and duck intake, seafood, vegetables, fruits

					only	0.19-<0.59<br>0.59-<2.18<br>2.18-<6.32<br>≥6.32	0.96 (0.85-1.08)<br>0.90 (0.77-1.04)<br>0.97 (0.86-1.09)<br>0.88 (0.78-1.00)	
					Total nuts and peanut butter, women	<0.95 g/d<br>0.95-<3.08<br>3.08-<7.30<br>7.30-<18.45<br>≥18.45	1.00<br>0.89 (0.80-0.99)<br>0.79 (0.70-0.89)<br>0.87 (0.77-0.98)<br>0.77 (0.67-0.88)	
					Nuts	<0.36 g/d<br>0.36-<0.66<br>0.66-<4.14<br>4.14-<8.63<br>≥8.63	1.00<br>0.83 (0.74-0.93)<br>0.84 (0.75-0.95)<br>0.77 (0.68-0.87)<br>0.75 (0.66-0.85)	
					Peanut butter intake only	<0.19 g/d<br>0.19-<0.59<br>0.59-<2.18<br>2.18-<6.32<br>≥6.32	1.00<br>0.86 (0.76-0.97)<br>0.81 (0.71-0.92)<br>0.94 (0.81-1.08)<br>0.86 (0.76-0.98)	
Luu HN et al, 2015, China	Shanghai Men's Health Study	2002-2006 - NA, 6.5 years follow-up	61123 men, age 40-74 years: 3387 deaths	Validated FFQ, 84 food items	Peanuts	<0.14 g/d<br>0.14-<0.72<br>0.72-<1.45<br>1.45-<2.54<br>≥2.54	1.00<br>0.87 (0.76-1.00)<br>0.77 (0.69-0.87)<br>0.82 (0.75-0.91)<br>0.83 (0.75-0.91)	Age, race, education, occupation, household income, marital status, smoking pack-years, alcohol, BMI, physical activity, vitamin supplement use, Charlson Comorbidity Index, metabolic conditions (hypertension, heart disease, DM, obesity, dyslipidemia), total energy, red meat, chicken and duck intake, seafood, vegetables, fruits
Luu HN et al, 2015, China	Shanghai Women's Health Study	1996-2000 – NA, 12.2 years follow-up	73142 women, age 40-70 years: 4757 deaths	Validated FFQ, 87 food items	Peanuts	<0.14 g/d<br>0.14-<0.72<br>0.72-<1.45<br>1.45-<2.54<br>≥2.54	1.00<br>0.80 (0.73-0.87)<br>0.79 (0.72-0.86)<br>0.80 (0.73-0.87)<br>0.83 (0.75-0.91)	Age, race, education, occupation, household income, marital status, smoking pack-years, alcohol, BMI, physical activity, vitamin supplement use, Charlson Comorbidity Index, metabolic conditions (hypertension, heart disease, DM, obesity, dyslipidemia), total energy, red meat, chicken and duck intake, seafood, vegetables, fruits

van den Brandt PA et al, 2015, Netherlands	Netherlands Cohort Study	1986-1996, 10 years follow-up	3202 subcohort members, men and women, age 55-69 years: 8823 deaths	Validated FFQ, 150 food items	Total nuts, all<br><br><br><br>Peanuts<br><br><br>Tree nuts<br><br><br>Peanut butter<br><br><br>Tree nuts, men<br><br><br><br>Tree nuts, women	0 g/d<br>0.1-<5<br>5-<10<br>≥10<br>0 g/d<br>0.1-<5<br>≥5<br>0 g/d<br>0.1-<5<br>≥5<br>0 g/d<br>0.1-<5<br>≥5<br>0 g/d<br>0.1-<5<br>5-<10<br>≥10<br>0 g/d<br>0.1-<5<br>5-<10<br>≥10	1.00<br>0.88 (0.78-0.99)<br>0.74 (0.63-0.88)<br>0.77 (0.66-0.89)<br>1.00<br>0.87 (0.77-0.98)<br>0.76 (0.66-0.87)<br>1.00<br>0.93 (0.82-1.05)<br>0.83 (0.66-1.06)<br>1.00<br>1.03 (0.90-1.17)<br>0.97 (0.81-1.15)<br>1.00<br>0.86 (0.72-1.02)<br>0.71 (0.57-0.88)<br>0.76 (0.63-0.92)<br>1.00<br>0.87 (0.74-1.02)<br>0.79 (0.61-1.01)<br>0.79 (0.63-1.00)	Age, sex, cigarette smoking, number of cigarettes per day, years of smoking, hypertension, DM, body height, BMI, non-occupational physical activity, highest level of education, alcohol, vegetables, fruits, energy, nutritional supplement use, women: HRT
Gopinath B et al, 2015, Australia	The Blue Mountains Eye Study	1992-1994 - 2007, 15 years follow-up	2893 men and women, age ≥49 years: 1044 deaths	Validated FFQ, 145 food items	Nuts, all<br><br><br>Nuts, women<br><br><br>Nuts, men	0-0.50 g/d<br>0.90-4.55<br>4.90-100<br>0-0.50 g/d<br>0.90-4.55<br>4.90-100<br>0-0.50 g/d<br>0.90-4.55<br>4.90-100	1.00<br>0.76 (0.65-0.89)<br>0.93 (0.72-1.08)<br>1.00<br>0.73 (0.58-0.92)<br>0.93 (0.73-1.18)<br>1.00<br>0.78 (0.63-0.97)<br>0.89 (0.72-1.10)	Age, sex, qualifications, total diet score, BMI, current smoking status, alcohol, self-rated health, walking disability, hypertension, diabetes, doctor-diagnosed history of cancer, angina, stroke, acute myocardial infarction
Bonaccio M et al, 2015, Italy	The Moli-sani Study	2005-2010 – 2011, 4.3 years follow-up	19386 men and women, mean age 54.5 years: 334 deaths	Validated FFQ, 188 food items	Nuts	Never<br>≤2 times/mo<br>3-7/mo<br>≥8/mo	1.00<br>0.68 (0.43-0.87)<br>0.56 (0.31-1.00)<br>0.53 (0.32-0.90)	Age, sex, education, smoking status, leisure-time physical activity, BMI, energy intake, Mediterranean diet score without nuts (including vegetables, legumes, fruit, dairy products, cereals, meat and meat products, fish, alcohol, MUFA/PUFA)
Wang JB et al, 2016, China	Linxian Nutrition	1984-1991 - 2010, 19-	2445 men and women, age 40-69	FFQ, 64 food items	Nuts	Per 3 times/mo	0.99 (0.96-1.02)	Age, sex, commune, smoking, drinking, season, BMI

	Intervention Trial cohort	26 years follow-up	years: 1501 deaths					
Eslamparast T et al, 2016, Iran	Golestan Cohort Study	2004 - 2013, 7 years follow-up	50045 men and women, age ≥40 years: 3981 deaths	Validated FFQ	Nuts, all<br><br><br><br>Nuts, women<br><br><br><br>Nuts, men	Never<br><1 serv/wk<br>1-<3<br>≥3<br>Never<br><1 serv/wk<br>1-<3<br>≥3<br>Never<br><1 serv/wk<br>1-<3<br>≥3	1.00<br>0.89 (0.82-0.95)<br>0.75 (0.67-0.85)<br>0.71 (0.58-0.86)<br>1.00<br>0.82 (0.74-0.91)<br>0.65 (0.54-0.79)<br>0.49 (0.34-0.71)<br>1.00<br>0.94 (0.85-1.03)<br>0.82 (0.70-0.96)<br>0.84 (0.66-1.07)	Age, sex, BMI, education, place of residence, smoking status, opium, alcohol, physical activity, wealth score, diabetes, hypertension, total energy, fish, red meat, chicken, fruits, vegetables, dairy products, eggs, total fiber, magnesium, zinc, copper

BMI=body mass index, CVD=cardiovascular disease, DM=diabetes mellitus, FFQ=food frequency questionnaire, FH=family history, HRT=hormone replacement therapy, MI=myocardial infarction, NA=not available, SFA=saturated fatty acids, WHR=waist-to-hip ratio

Supplementary table 8. Nut consumption and respiratory disease mortality

Author, publication year, country	Study name	Study period	Number of participants, gender, age, number of deaths	Dietary assessment	Exposure and subgroup	Nut consumption frequency or amount	Relative risks (95% confidence intervals)	Adjustment for confounding factors
Bao Y et al, 2013, USA	Nurses' Health Study	1980-2010, 30 years follow-up	76464 women, age 34-59 years: 809 respiratory disease deaths	Validated FFQ, 61-116 food items	Nuts<br><br><br><br><br>Peanuts<br><br><br><br>Tree nuts	Never<br><1/wk<br>1<br>2-4<br>≥5<br>Never<br><1/wk<br>1<br>≥2<br>Never<br><1/wk<br>1<br>≥2	1.00<br>0.99 (0.84-1.15)<br>0.86 (0.71-1.04)<br>0.74 (0.60-0.92)<br>0.65 (0.45-0.94)<br>1.00<br>0.89 (0.77-1.03)<br>0.73 (0.56-0.96)<br>0.79 (0.61-1.04)<br>1.00<br>1.11 (0.96-1.29)<br>0.72 (0.53-0.98)<br>0.82 (0.59-1.15)	Age, race, BMI, physical activity, smoking, screening, multivitamin use, aspirin use, FH – DM, MI or cancer, history of DM, hypertension, or hypercholesterolemia, total energy, alcohol, red or processed meat, fruits, vegetables, menopausal status, HRT
Bao Y et al, 2013, USA	Health Professionals Follow-up Study	1986-2010, 24 years follow-up	42498 men, age 45-70 years: 1192 respiratory disease deaths	Validated FFQ, 131 food items	Nuts<br><br><br><br><br>Peanuts<br><br><br><br>Tree nuts	Never<br><1/wk<br>1<br>2-4<br>≥5<br>Never<br><1/wk<br>1<br>≥2<br>Never<br><1/wk<br>1<br>≥2	1.00<br>0.89 (0.71-1.11)<br>0.99 (0.78-1.24)<br>0.94 (0.75-1.18)<br>0.84 (0.64-1.11)<br>1.00<br>0.94 (0.78-1.13)<br>1.03 (0.81-1.30)<br>0.87 (0.70-1.08)<br>1.00<br>1.03 (0.86-1.22)<br>1.00 (0.79-1.27)<br>0.94 (0.74-1.20)	Age, race, BMI, physical activity, smoking, screening, multivitamin use, aspirin use, FH – DM, MI or cancer, history of DM, hypertension, or hypercholesterolemia, total energy, alcohol, red or processed meat, fruits, vegetables
van den Brandt PA et al, 2015, Netherlands	Netherlands Cohort Study	1986-1996, 10 years follow-up	3202 subcohort members, men and women, age 55-69 years: 550 respiratory disease deaths	Validated FFQ, 150 food items	Total nuts, all<br><br><br><br>Peanuts	0 g/d<br>0.1-<5<br>5-<10<br>≥10<br>0 g/d<br>0.1-<5<br>≥5	1.00<br>0.67 (0.52-0.88)<br>0.58 (0.39-0.87)<br>0.61 (0.43-0.87)<br>1.00<br>0.68 (0.52-0.88)<br>0.61 (0.44-0.83)	Age, sex, cigarette smoking, number of cigarettes per day, years of smoking, hypertension, DM, body height, BMI, non-occupational physical activity, highest level of education, alcohol, vegetables, fruits, energy, nutritional supplement use, women: HRT

					Tree nuts	0 g/d	1.00	
						0.1-<5	0.75 (0.56-1.01)	
						≥5	0.82 (0.44-1.54)	
					Peanut butter	0 g/d	1.00	
						0.1-<5	1.23 (0.92-1.64)	
						≥5	0.77 (0.50-1.18)	

BMI=body mass index, DM=diabetes mellitus, FFQ=food frequency questionnaire, FH=family history, HRT=hormone replacement therapy, MI=myocardial infarction

Supplementary table 9. Nut consumption and diabetes mortality

Author, publication year, country	Study name	Study period	Number of participants, gender, age, number of deaths	Dietary assessment	Exposure and subgroup	Nut consumption frequency or amount	Relative risks (95% confidence intervals)	Adjustment for confounding factors
Bao Y et al, 2013, USA	Nurses' Health Study	1980-2010, 30 years follow-up	76464 women, age 34-59 years: 220 diabetes deaths	Validated FFQ, 61-116 food items	Nuts<br><br><br><br><br>Peanuts<br><br><br><br>Tree nuts	Never<br><1/wk<br>1<br>2-4<br>≥5<br>Never<br><1/wk<br>1<br>≥2<br>Never<br><1/wk<br>1<br>≥2	1.00<br>0.86 (0.62-1.20)<br>0.68 (0.42-1.10)<br>0.70 (0.41-1.18)<br>0.83 (0.35-1.96)<br>1.00<br>0.76 (0.52-1.10)<br>1.08 (0.58-2.00)<br>0.98 (0.47-2.03)<br>1.00<br>0.68 (0.46-1.00)<br>0.98 (0.48-2.02)<br>1.15 (0.54-2.48)	Age, race, BMI, physical activity, smoking, screening, multivitamin use, aspirin use, FH – DM, MI or cancer, history of DM, hypertension, or hypercholesterolemia, total energy, alcohol, red or processed meat, fruits, vegetables, menopausal status, HRT
Bao Y et al, 2013, USA	Health Professionals Follow-up Study	1986-2010, 24 years follow-up	42498 men, age 45-70 years: 84 diabetes deaths	Validated FFQ, 131 food items	Nuts<br><br><br><br><br>Peanuts<br><br><br><br>Tree nuts	Never<br><1/wk<br>1<br>2-4<br>≥5<br>Never<br><1/wk<br>1<br>≥2<br>Never<br><1/wk<br>1<br>≥2	1.00<br>1.32 (0.65-2.65)<br>0.76 (0.34-1.68)<br>1.02 (0.49-2.12)<br>0.85 (0.35-2.11)<br>1.00<br>0.84 (0.47-1.51)<br>0.89 (0.43-1.84)<br>0.63 (0.31-1.28)<br>1.00<br>0.95 (0.54-1.66)<br>1.12 (0.53-2.37)<br>0.89 (0.42-1.89)	Age, race, BMI, physical activity, smoking, screening, multivitamin use, aspirin use, FH – DM, MI or cancer, history of DM, hypertension, or hypercholesterolemia, total energy, alcohol, red or processed meat, fruits, vegetables
Luu HN et al, 2015, USA	Southern Community Cohort Study	2002-2009 - NA, 5.4 years follow-up	71764 men and women, age 40-79 years: 338 diabetes deaths	FFQ, 89 food items	Total nuts and peanut butter, African Americans<br><br><br><br>Total nuts and peanut butter, Caucasians	<0.95 g/d<br>0.95-<3.08<br>3.08-<7.30<br>7.30-<18.45<br>≥18.45<br><0.95 g/d<br>0.95-<3.08	1.00<br>0.81 (0.56-1.19)<br>0.84 (0.56-1.26)<br>0.78 (0.52-1.17)<br>0.55 (0.35-0.88)<br>1.00<br>1.00 (0.48-2.08)	Age, sex, race, education, occupation, household income, marital status, smoking pack-years, alcohol, BMI, physical activity, vitamin supplement use, Charlson Comorbidity Index, metabolic conditions (hypertension, heart disease, DM, obesity,

						3.08-<7.30<br>7.30-<18.45<br>≥18.45	0.65 (0.29-1.46)<br>1.35 (0.68-2.67)<br>0.76 (0.34-1.70)	hypercholesterolemia), total energy, red meat, chicken and duck intake, seafood, vegetables, fruits
Luu HN et al, 2015, China	Shanghai Men's Health Study	2002-2006 - NA, 6.5 years follow-up	61123 men, age 40-74 years: 125 diabetes deaths	Validated FFQ, 84 food items	Peanuts	<0.14 g/d<br>0.14-<0.72<br>0.72-<1.45<br>1.45-<2.54<br>・2.54	1.00<br>1.43 (0.74-2.74)<br>1.02 (0.56-1.85)<br>0.86 (0.50-1.47)<br>1.38 (0.83-2.30)	Age, race, education, occupation, household income, marital status, smoking pack-years, alcohol, BMI, physical activity, vitamin supplement use, Charlson Comorbidity Index, metabolic conditions (hypertension, heart disease, DM, obesity, dyslipidemia), total energy, red meat, chicken and duck intake, seafood, vegetables, fruits
Luu HN et al, 2015, China	Shanghai Women's Health Study	1996-2000 – NA, 12.2 years follow-up	73142 women, age 40-70 years: 314 diabetes deaths	Validated FFQ, 87 food items	Peanuts	<0.14 g/d<br>0.14-<0.72<br>0.72-<1.45<br>1.45-<2.54<br>・2.54	1.00<br>0.71 (0.51-1.00)<br>0.48 (0.33-0.70)<br>0.91 (0.67-1.25)<br>0.84 (0.59-1.20)	Age, race, education, occupation, household income, marital status, smoking pack-years, alcohol, BMI, physical activity, vitamin supplement use, Charlson Comorbidity Index, metabolic conditions (hypertension, heart disease, DM, obesity, dyslipidemia), total energy, red meat, chicken and duck intake, seafood, vegetables, fruits
van den Brandt PA et al, 2015, Netherlands	Netherlands Cohort Study	1986-1996, 10 years follow-up	3202 subcohort members, men and women, age 55-69 years: 158 diabetes deaths	Validated FFQ, 150 food items	Total nuts, all<br><br><br><br>Peanuts<br><br><br>Tree nuts<br><br><br>Peanut butter	0 g/d<br>0.1-<5<br>5-<10<br>≥10<br>0 g/d<br>0.1-<5<br>≥5<br>0 g/d<br>0.1-<5<br>≥5<br>0 g/d<br>0.1-<5<br>≥5	1.00<br>0.45 (0.24-0.83)<br>0.22 (0.08-0.63)<br>0.70 (0.32-1.51)<br>1.00<br>0.40 (0.22-0.75)<br>0.45 (0.21-0.96)<br>1.00<br>0.79 (0.38-1.64)<br>2.00 (0.76-5.27)<br>1.00<br>0.68 (0.29-1.60)<br>0.56 (0.22-1.45)	Age, sex, cigarette smoking, number of cigarettes per day, years of smoking, hypertension, DM, body height, BMI, non-occupational physical activity, highest level of education, alcohol, vegetables, fruits, energy, nutritional supplement use, women: HRT

BMI=body mass index, DM=diabetes mellitus, FFQ=food frequency questionnaire, FH=family history, HRT=hormone replacement therapy, MI=myocardial infarction, NA=not available

Supplementary table 10. Nut consumption and neurodegenerative disease mortality

Author, publication year, country	Study name	Study period	Number of participants, gender, age, number of deaths	Dietary assessment	Exposure and subgroup	Nut consumption frequency or amount	Relative risks (95% confidence intervals)	Adjustment for confounding factors
Bao Y et al, 2013, USA	Nurses' Health Study	1980-2010, 30 years follow-up	76464 women, age 34-59 years: 1315 neurodegenerative disease deaths	Validated FFQ, 61-116 food items	Nuts<br><br><br><br><br>Peanuts<br><br><br><br>Tree nuts	Never<br><1/wk<br>1<br>2-4<br>≥5<br>Never<br><1/wk<br>1<br>≥2<br>Never<br><1/wk<br>1<br>≥2	1.00<br>1.07 (0.92-1.25)<br>1.04 (0.87-1.26)<br>0.88 (0.72-1.08)<br>0.95 (0.70-1.29)<br>1.00<br>1.08 (0.94-1.24)<br>1.03 (0.81-1.30)<br>0.91 (0.70-1.20)<br>1.00<br>1.06 (0.93-1.22)<br>0.65 (0.48-0.88)<br>0.81 (0.60-1.11)	Age, race, BMI, physical activity, smoking, screening, multivitamin use, aspirin use, FH – DM, MI or cancer, history of DM, hypertension, or hypercholesterolemia, total energy, alcohol, red or processed meat, fruits, vegetables, menopausal status, HRT
Bao Y et al, 2013, USA	Health Professionals Follow-up Study	1986-2010, 24 years follow-up	42498 men, age 45-70 years: 654 neurodegenerative disease deaths	Validated FFQ, 131 food items	Nuts<br><br><br><br><br>Peanuts<br><br><br><br>Tree nuts	Never<br><1/wk<br>1<br>2-4<br>≥5<br>Never<br><1/wk<br>1<br>≥2<br>Never<br><1/wk<br>1<br>≥2	1.00<br>1.23 (0.94-1.59)<br>1.20 (0.92-1.57)<br>1.17 (0.90-1.53)<br>1.01 (0.74-1.39)<br>1.00<br>1.11 (0.90-1.38)<br>1.27 (0.98-1.64)<br>1.11 (0.87-1.42)<br>1.00<br>1.37 (1.13-1.67)<br>1.22 (0.93-1.59)<br>1.09 (0.83-1.42)	Age, race, BMI, physical activity, smoking, screening, multivitamin use, aspirin use, FH – DM, MI or cancer, history of DM, hypertension, or hypercholesterolemia, total energy, alcohol, red or processed meat, fruits, vegetables
van den Brandt PA et al, 2015, Netherlands	Netherlands Cohort Study	1986-1996, 10 years follow-up	3202 subcohort members, men and women, age 55-69 years: 87 neurodegenerative disease deaths	Validated FFQ, 150 food items	Total nuts, all<br><br><br><br>Peanuts	0 g/d<br>0.1-<5<br>5-<10<br>≥10<br>0 g/d<br>0.1-<5	1.00<br>0.64 (0.38-1.09)<br>0.36 (0.13-0.97)<br>0.53 (0.25-1.14)<br>1.00<br>0.70 (0.41-1.18)	Age, sex, cigarette smoking, number of cigarettes per day, years of smoking, hypertension, DM, body height, BMI, non-occupational physical activity, highest level of education, alcohol, vegetables, fruits, energy, nutritional

						≥5	0.56 (0.28-1.15)	supplement use, women: HRT
					Tree nuts	0 g/d	1.00	
						0.1-<5	0.97 (0.56-1.68)	
						≥5	0.68 (0.20-2.37)	
					Peanut butter	0 g/d	1.00	
						0.1-<5	1.01 (0.53-1.91)	
						≥5	0.98 (0.44-2.19)	

BMI=body mass index, DM=diabetes mellitus, FFQ=food frequency questionnaire, FH=family history, HRT=hormone replacement therapy, MI=myocardial infarction

Supplementary table 11. Nut consumption and infectious disease mortality

Author, publication year, country	Study name	Study period	Number of participants, gender, age, number of deaths	Dietary assessment	Exposure and subgroup	Nut consumption frequency or amount	Relative risks (95% confidence intervals)	Adjustment for confounding factors
Bao Y et al, 2013, USA	Nurses' Health Study	1980-2010, 30 years follow-up	76464 women, age 34-59 years: 217 infectious disease deaths	Validated FFQ, 61-116 food items	Nuts	Never	1.00	Age, race, BMI, physical activity, smoking, screening, multivitamin use, aspirin use, FH – DM, MI or cancer, history of DM, hypertension, or hypercholesterolemia, total energy, alcohol, red or processed meat, fruits, vegetables, menopausal status, HRT
						<1/wk	0.97 (0.68-1.40)	
						1	0.79 (0.49-1.25)	
						2-4	0.71 (0.43-1.18)	
						≥5	0.65 (0.28-1.56)	
					Peanuts	Never	1.00	
						<1/wk	0.71 (0.51-1.00)	
						1	0.44 (0.22-0.88)	
						≥2	0.67 (0.36-1.26)	
					Tree nuts	Never	1.00	
						<1/wk	0.90 (0.64-1.27)	
						1	0.58 (0.29-1.18)	
						≥2	0.67 (0.32-1.43)	
Bao Y et al, 2013, USA	Health Professionals Follow-up Study	1986-2010, 24 years follow-up	42498 men, age 45-70 years: 180 infectious disease deaths	Validated FFQ, 131 food items	Nuts	Never	1.00	Age, race, BMI, physical activity, smoking, screening, multivitamin use, aspirin use, FH – DM, MI or cancer, history of DM, hypertension, or hypercholesterolemia, total energy, alcohol, red or processed meat, fruits, vegetables
						<1/wk	1.28 (0.79-2.05)	
						1	0.96 (0.58-1.60)	
						2-4	0.91 (0.55-1.51)	
						≥5	0.84 (0.44-1.58)	
					Peanuts	Never	1.00	
						<1/wk	0.98 (0.67-1.45)	
						1	0.90 (0.55-1.46)	
						≥2	0.68 (0.42-1.12)	
					Tree nuts	Never	1.00	
						<1/wk	1.13 (0.79-1.63)	
						1	1.17 (0.73-1.89)	
						≥2	0.76 (0.44-1.31)	

BMI=body mass index, DM=diabetes mellitus, FFQ=food frequency questionnaire, FH=family history, MI=myocardial infarction

Supplementary table 12. Nut consumption and kidney disease mortality

Author, publication year, country	Study name	Study period	Number of participants, gender, age, number of deaths	Dietary assessment	Exposure and subgroup	Nut consumption frequency or amount	Relative risks (95% confidence intervals)	Adjustment for confounding factors
Bao Y et al, 2013, USA	Nurses' Health Study	1980-2010, 30 years follow-up	76464 women, age 34-59 years: 191 kidney disease deaths	Validated FFQ, 61-116 food items	Nuts<br><br><br><br><br>Peanuts<br><br><br><br>Tree nuts	Never<br><1/wk<br>1<br>2-4<br>≥5<br>Never<br><1/wk<br>1<br>≥2<br>Never<br><1/wk<br>1<br>≥2	1.00<br>0.92 (0.63-1.33)<br>0.83 (0.51-1.35)<br>0.95 (0.57-1.60)<br>0.89 (0.37-2.11)<br>1.00<br>0.86 (0.59-1.25)<br>0.71 (0.36-1.40)<br>0.75 (0.37-1.54)<br>1.00<br>0.96 (0.66-1.39)<br>0.95 (0.46-1.94)<br>0.84 (0.35-1.98)	Age, race, BMI, physical activity, smoking, screening, multivitamin use, aspirin use, FH – DM, MI or cancer, history of DM, hypertension, or hypercholesterolemia, total energy, alcohol, red or processed meat, fruits, vegetables, menopausal status, HRT
Bao Y et al, 2013, USA	Health Professionals Follow-up Study	1986-2010, 24 years follow-up	42498 men, age 45-70 years: 176 kidney disease deaths	Validated FFQ, 131 food items	Nuts<br><br><br><br><br>Peanuts<br><br><br><br>Tree nuts	Never<br><1/wk<br>1<br>2-4<br>≥5<br>Never<br><1/wk<br>1<br>≥2<br>Never<br><1/wk<br>1<br>≥2	1.00<br>0.84 (0.54-1.31)<br>0.91 (0.58-1.43)<br>0.52 (0.31-0.86)<br>0.49 (0.26-0.92)<br>1.00<br>0.76 (0.52-1.09)<br>0.82 (0.51-1.31)<br>0.39 (0.23-0.66)<br>1.00<br>0.74 (0.52-1.06)<br>1.00 (0.62-1.62)<br>0.58 (0.33-1.00)	Age, race, BMI, physical activity, smoking, screening, multivitamin use, aspirin use, FH – DM, MI or cancer, history of DM, hypertension, or hypercholesterolemia, total energy, alcohol, red or processed meat, fruits, vegetables

BMI=body mass index, DM=diabetes mellitus, FFQ=food frequency questionnaire, FH=family history, HRT=hormone replacement therapy, MI=myocardial infarction

Supplementary table 13. Nut consumption and inflammatory disease mortality

Author, publication year, country	Study name	Study period	Number of participants, gender, age, number of deaths	Dietary assessment	Exposure and subgroup	Nut consumption frequency or amount	Relative risks (95% confidence intervals)	Adjustment for confounding factors
Gopinath B et al, 2011, Australia	Blue Mountains Eye Study	1992-1994 - 2007, 15 years follow-up	2514 men and women, age ≥49 years: 214 inflammatory disease deaths	FFQ, 145 food items	Nuts	0.00-0.90 g/d<br>1.40-4.55<br>4.90-100.0	1.00<br>0.49 (0.33-0.72)<br>0.68 (0.48-0.98)	Age, sex, current smoking, alcohol, poor self-rated health, BMI, diabetes, total fiber, glycemic index, use of corticosteroid drugs, white blood cell count

Supplementary Table 14. Relative risks from nonlinear dose-response analysis of peanuts and coronary heart disease, stroke, cardiovascular disease, total cancer, and all-cause mortality

	**Coronary heart disease**			**Stroke**			**Cardiovascular disease**		
	**Incidence/ mortality**	**Incidence**	**Mortality**	**Incidence/ mortality**	**Incidence**	**Mortality**	**Incidence/ mortality**	**Incidence**	**Mortality**
**g/d**	**RR (95% CI)**	**RR (95% CI)**	**RR (95% CI)**	**RR (95% CI)**	**RR (95% CI)**	**RR (95% CI)**	**RR (95% CI)**	**RR (95% CI)**	**RR (95% CI)**
0	1.00	1.00	1.00	1.00	1.00	1.00	1.00	1.00	1.00
5	0.89 (0.87-0.90)	0.88 (0.84-0.94)	0.88 (0.86-0.90)	0.94 (0.92-0.96)	0.96 (0.93-0.99)	0.97 (0.92-1.01)	0.88 (0.87-0.90)	0.88 (0.83-0.92)	0.88 (0.87-0.89)
10	0.82 (0.79-0.84)	0.83 (0.77-0.88)	0.81 (0.78-0.83)	0.92 (0.89-0.95)	0.95 (0.92-0.99)	0.95 (0.90-1.01)	0.83 (0.81-0.84)	0.85 (0.80-0.91)	0.82 (0.80-0.84)
15	0.78 (0.76-0.81)	0.81 (0.76-0.86)	0.77 (0.74-0.79)	0.94 (0.91-0.97)	0.96 (0.92-1.00)	0.96 (0.90-1.02)	0.81 (0.79-0.83)	0.87 (0.82-0.93)	0.80 (0.79-0.82)
20	0.77 (0.74-0.79)	0.81 (0.74-0.89)	0.75 (0.72-0.77)	0.97 (0.94-1.01)	0.97 (0.92-1.02)	0.97 (0.91-1.04)	0.82 (0.81-0.84)	0.89 (0.84-0.95)	0.81 (0.80-0.83)
25	0.76 (0.73-0.79)	0.83 (0.70-0.98)	0.74 (0.71-0.77)	1.02 (0.98-1.07)	0.98 (0.91-1.04)	0.99 (0.91-1.08)	0.85 (0.83-0.87)		0.84 (0.82-0.86)
28	0.75 (0.71-0.80)	0.84 (0.68-1.05)	0.73 (0.70-0.77)	1.05 (1.00-1.12)	0.98 (0.91-1.06)		0.86 (0.84-0.89)		0.85 (0.83-0.88)
$p_{nonlinearity}$	<0.0001	0.04	<0.0001	<0.0001	0.05	0.18	<0.0001	<0.0001	<0.0001

Supplementary Table 15. Relative risks from nonlinear dose-response analysis of peanuts and mortality from total cancer, all causes, respiratory disease, diabetes, neurodegenerative disease.

	**Total cancer**	**All-cause mortality**	**Respiratory disease**	**Diabetes**	**Neurodegenerative disease**
**g/d**	**RR (95% CI)**	**RR (95% CI)**	**RR (95% CI)**	**RR (95% CI)**	**RR (95% CI)**
0	1.00	1.00	1.00	1.00	1.00
5	0.93 (0.91-0.96)	0.89 (0.87-0.91)	0.78 (0.69-0.90)	0.80 (0.69-0.93)	0.99 (0.93-1.06)
10	0.90 (0.86-0.94)	0.84 (0.81-0.87)	0.72 (0.63-0.82)	0.72 (0.60-0.86)	0.82 (0.75-0.90)
15	0.88 (0.85-0.92)	0.82 (0.79-0.85)	0.67 (0.56-0.80)	0.71 (0.62-0.82)	0.67 (0.55-0.80)
20	0.88 (0.85-0.91)	0.82 (0.80-0.84)	0.63 (0.49-0.81)	0.74 (0.63-0.86)	0.55 (0.42-0.73)
25	0.89 (0.85-0.92)	0.84 (0.82-0.85)	-	0.76 (0.60-0.97)	-
28	-	0.85 (0.83-0.86)	-	-	-
$p_{nonlinearity}$	0.003	<0.0001	0.10	0.07	0.009

Supplementary table 16: Subgroup analyses of nuts and coronary heart disease, stroke, and cardiovascular disease, per 28 grams per day

		**Coronary heart disease**					**Stroke**					**Cardiovascular disease**				
		n	RR (95% CI)	I^2	P_h^1	P_h^2	*n*	RR (95% CI)	I^2	P_h^1	P_h^2	*n*	RR (95% CI)	I^2	P_h^1	P_h^2
All studies		11	0.71 (0.63-0.80)	47.4	0.04		11	0.93 (0.83-1.05)	13.7	0.31		12	0.79 (0.70-0.88)	59.6	0.004	
Duration of follow-up																
<10 yrs follow-up		2	0.57 (0.47-0.69)	0	0.42	0.05	3	0.94 (0.74-1.18)	0	0.39	0.77	5	0.67 (0.54-0.84)	46.6	0.11	0.09
≥10 yrs follow-up		9	0.75 (0.67-0.84)	30.5	0.18		8	0.92 (0.79-1.07)	27.6	0.21		7	0.85 (0.76-0.95)	47.7	0.08	
Outcome																
Incidence		4	0.80 (0.65-0.99)	54.8	0.09	0.05	6	0.96 (0.82-1.11)	35.1	0.17	0.92	4	0.83 (0.64-1.08)	50.2	0.11	0.48
Mortality		9	0.69 (0.63-0.75)	0	0.54		7	0.95 (0.79-1.15)	0	0.64		9	0.76 (0.67-0.86)	64.9	0.004	
Outcome subtype																
Myocardial infarction		2	0.63 (0.35-1.13)	57.7	0.12	0.87	-				-	-				-
Coronary heart disease		10	0.67 (0.57-0.79)	65.8	0.002		-					-				
Ischemic stroke		-				-	6	1.01 (0.88-1.17)	15.1	0.32	0.46	-				-
Hemorrhagic stroke		-					5	1.15 (0.86-1.54)	9.1	0.35		-				
Gender																
Men		4	0.70 (0.62-0.80)	0	0.79	0.74/ 0.96	5	1.07 (0.74-1.55)	58.5	0.05	0.48/ 0.23	5	0.73 (0.66-0.81)	0	0.79	0.75 / 0.02
Women		4	0.71 (0.61-0.82)	0	0.80		6	0.88 (0.78-1.00)	16.2	0.31		6	0.86 (0.72-1.03)	62.1	0.02	
Men and women		5	0.69 (0.52-0.91)	75.7	0.002		2	1.08 (0.66-1.78)	0	0.32		4	0.72 (0.54-0.95)	40.7	0.17	
Geographic location																
Europe		2	0.74 (0.60-0.92)	0	0.96	0.72	2	0.94 (0.44-2.02)	62.8	0.10	0.77	3	0.73 (0.50-1.06)	57.6	0.10	0.60
America		6	0.68 (0.62-0.75)	0	0.47		7	0.95 (0.83-1.09)	24.6	0.24		6	0.83 (0.73-0.93)	66.9	0.01	
Asia		1	0.31 (0.13-0.81)				1	0.90 (0.48-1.64)				2	0.55 (0.39-0.78)	0	0.41	
Australia		1	0.98 (0.38-2.53)				1	0.75 (0.17-3.25)				1	0.79 (0.35-1.79)			
Number of cases																
Cases <500		3	0.64 (0.37-1.10)	47.4	0.15	0.21	5	1.01 (0.73-1.41)	0	0.63	0.59	2	0.67 (0.29-1.55)	78.7	0.03	0.40
Cases 500-<1000		3	0.62 (0.53-0.74)	24.5	0.27		2	0.90 (0.58-1.38)	70.6	0.07		3	0.67 (0.52-0.87)	0	0.92	
Cases ≥1000		5	0.77 (0.68-0.88)	40.8	0.15		4	0.90 (0.75-1.08)	42.6	0.16		7	0.81 (0.72-0.92)	68.5	0.004	
Study quality																
0-3 stars		0				0.60	0				0.93	0				0.32
4-6		2	0.53 (0.23-1.22)	69.0	0.07		1	0.90 (0.48-1.64)				2	0.66 (0.50-0.87)	0	0.88	
7-9		9	0.72 (0.64-0.81)	48.3	0.05		10	0.93 (0.82-1.06)	22.1	0.24		10	0.80 (0.71-0.91)	62.9	0.004	
Adjustment for confounding factors																
Age	Yes	11	0.71 (0.63-0.80)	47.4	0.04	NC	11	0.93 (0.83-1.05)	13.7	0.31	NC	12	0.79 (0.70-0.88)	59.6	0.004	NC
	No	0					0					0				
Education	Yes	4	0.79 (0.61-1.01)	59.7	0.06	0.20	7	0.94 (0.79-1.12)	10.9	0.35	0.89	6	0.79 (0.66-0.96)	50.2	0.07	0.86
	No	7	0.68 (0.61-0.76)	18.0	0.29		4	0.92 (0.75-1.12)	38.0	0.18		6	0.78 (0.67-0.91)	68.4	0.007	

Family history of CHD	Yes	2	0.72 (0.63-0.81)	0	0.84	0.94	2	0.81 (0.66-1.01)	5.8	0.30	0.18	2	0.78 (0.70-0.87)	0	0.41	0.89
	No	9	0.70 (0.59-0.83)	57.7	0.02		9	0.99 (0.88-1.12)	0	0.44		10	0.77 (0.66-0.90)	63.3	0.004	
Body mass index	Yes	10	0.71 (0.62-0.80)	52.6	0.03	0.85	11	0.93 (0.83-1.05)	13.7	0.31	NC	11	0.77 (0.68-0.86)	59.1	0.007	0.15
	No	1	0.74 (0.54-1.01)				0					1	1.04 (0.80-1.36)			
Smoking	Yes	10	0.71 (0.62-0.81)	52.5	0.03	0.99	11	0.93 (0.83-1.05)	13.7	0.31	NC	11	0.76 (0.69-0.85)	36.1	0.11	0.02
	No	1	0.71 (0.57-0.89)				0					1	0.94 (0.86-1.02)			
Alcohol	Yes	9	0.73 (0.65-0.83)	40.8	0.10	0.27	11	0.93 (0.83-1.05)	13.7	0.31	NC	12	0.79 (0.70-0.88)	59.6	0.004	NC
	No	2	0.62 (0.44-0.86)	59.5	0.12		0					0				
Physical activity	Yes	8	0.71 (0.63-0.81)	53.6	0.04	0.80	9	0.93 (0.81-1.07)	30.2	0.18	0.85	10	0.79 (0.70-0.89)	65.6	0.002	0.59
	No	3	0.64 (0.37-1.10)	47.4	0.15		2	0.88 (0.50-1.55)	0	0.81		2	0.68 (0.44-1.05)	0	0.68	
Hypertension	Yes	8	0.72 (0.62-0.83)	54.6	0.03	0.70	8	0.99 (0.86-1.14)	11.3	0.34	0.18	9	0.75 (0.68-0.82)	18.5	0.28	0.00 7
	No	3	0.67 (0.51-0.88)	39.5	0.19		3	0.82 (0.68-1.00)	0	0.56		3	0.93 (0.81-1.08)	27.4	0.25	
Hypercholesterolemia, serum cholesterol	Yes	5	0.75 (0.64-0.87)	56.4	0.06	0.34	4	1.01 (0.85-1.19)	0	0.45	0.33	5	0.77 (0.68-0.85)	31.4	0.21	0.69
	No	6	0.66 (0.55-0.79)	32.9	0.19		7	0.87 (0.73-1.04)	25.2	0.24		7	0.79 (0.66-0.95)	59.8	0.02	
Coffee, caffeine	Yes	0				NC	0				NC	1	1.00 (0.62-1.55)			0.42
	No	11	0.71 (0.63-0.80)	47.4	0.04		11	0.93 (0.83-1.05)	13.7	0.31		11	0.78 (0.69-0.87)	62.4	0.003	
Sugar-sweetened beverages	Yes	0				NC	0				NC	1	1.00 (0.62-1.55)			0.42
	No	11	0.71 (0.63-0.80)	47.4	0.04		11	0.93 (0.83-1.05)	13.7	0.31		11	0.78 (0.69-0.87)	62.4	0.003	
Red or processed meat	Yes	6	0.70 (0.64-0.78)	0	0.91	0.83	6	0.95 (0.80-1.13)	19.9	0.28	0.69	9	0.77 (0.67-0.88)	65.3	0.003	0.58
	No	5	0.69 (0.52-0.91)	75.7	0.002		5	0.90 (0.74-1.10)	22.7	0.27		3	0.84 (0.64-1.09)	51.5	0.13	
Fish	Yes	4	0.68 (0.59-0.80)	0	0.74	0.76	7	0.94 (0.79-1.12)	20.4	0.27	0.77	6	0.74 (0.59-0.94)	72.3	0.003	0.81
	No	7	0.71 (0.60-0.84)	64.7	0.009		4	0.91 (0.75-1.11)	24.0	0.27		6	0.80 (0.71-0.89)	23.9	0.25	
Fruits and vegetables	Yes	7	0.71 (0.64-0.78)	0	0.95	0.95	7	0.91 (0.77-1.08)	28.9	0.21	0.77	10	0.77 (0.68-0.87)	61.6	0.005	0.40
	No	4	0.66 (0.45-0.96)	81.8	0.001		4	0.97 (0.81-1.16)	0.3	0.39		2	0.86 (0.54-1.37)	63.7	0.10	
Whole grains	Yes	2	0.62 (0.46-0.84)	62.0	0.11	0.25	5	0.93 (0.73-1.18)	45.3	0.12	0.91	2	0.94 (0.86-1.02)	0	0.79	0.01
	No	9	0.74 (0.65-0.84)	39.8	0.10		6	0.94 (0.80-1.09)	0	0.52		10	0.75 (0.68-0.84)	37.3	0.11	
Dairy	Yes	2	0.74 (0.52-1.05)	0	0.53	0.78	4	0.91 (0.74-1.13)	39.0	0.18	0.87	3	0.72 (0.43-1.19)	57.6	0.10	0.63
	No	9	0.70 (0.62-0.80)	56.9	0.02		7	0.94 (0.80-1.11)	9.9	0.35		9	0.80 (0.71-0.89)	62.9	0.006	
Energy intake	Yes	7	0.75 (0.67-0.84)	38.1	0.14	0.15	9	0.89 (0.78-1.03)	11.9	0.34	0.25	11	0.79 (0.71-0.89)	61.8	0.004	0.53
	No	4	0.61 (0.47-0.79)	43.5	0.15		2	1.07 (0.88-1.30)	0	0.57		1	0.64 (0.39-1.07)			

*n*denotes the number of studies.

[1] P for heterogeneity within each subgroup,

[2] P for heterogeneity between subgroups with meta-regression analysis,

[3] P for heterogeneity between men and women (studies with genders mixed were excluded),

NC = not calculable

Supplementary table 17: Subgroup analyses of nuts and total cancer and all-cause mortality, per 28 grams per day

		**Total cancer**					**All-cause mortality**				
		n	RR (95% CI)	I^2	P_h[1]	P_h[2]	*n*	RR (95% CI)	I^2	P_h[1]	P_h[2]
All studies		8	0.85 (0.76-0.94)	41.8	0.10		16	0.78 (0.72-0.84)	66.0	<0.0001	
Duration of follow-up											
<10 yrs follow-up		5	0.82 (0.66-1.01)	51.2	0.09	0.84	7	0.61 (0.49-0.76)	72.2	0.001	0.03
≥10 yrs follow-up		3	0.86 (0.77-0.97)	44.1	0.17		9	0.84 (0.79-0.90)	43.4	0.08	
Outcome											
Incidence		1	1.06 (0.79-1.32)			0.22	-	-	-	-	-
Mortality		7	0.83 (0.75-0.92)	36.3	0.15		16	0.78 (0.72-0.84)	66.0	<0.0001	
Gender											
Men		4	0.87 (0.78-0.97)	0	0.53	0.68/ 0.93	8	0.76 (0.70-0.83)	25.3	0.23	0.70/ 0.67
Women		3	0.82 (0.61-1.09)	59.6	0.08		8	0.76 (0.64-0.88)	79.8	<0.0001	
Men and women		2	0.83 (0.48-1.44)	73.2	0.05		6	0.74 (0.58-0.95)	59.9	0.03	
Geographic location											
Europe		3	0.80 (0.58-1.10)	69.9	0.04	0.99	6	0.70 (0.56-0.89)	62.4	0.02	0.96
America		4	0.88 (0.82-0.95)	0	0.72		7	0.82 (0.77-0.88)	55.4	0.04	
Asia		1	0.41 (0.18-0.91)				2	0.62 (0.29-1.31)	90.7	0.001	
Australia		0					1	0.82 (0.37-1.79)			
Number of cases											
Cases <500		1	0.60 (0.36-1.00)			0.80	5	0.60 (0.46-0.79)	27.4	0.24	0.11
Cases 500-<1000		3	0.85 (0.60-1.21)	61.8	0.07		0				
Cases ≥1000		4	0.85 (0.78-0.94)	30.4	0.23		11	0.80 (0.74-0.87)	68.9	<0.0001	
Study quality											
0-3 stars		0				0.92	0				0.88
4-6		1	0.87 (0.65-1.16)				4	0.75 (0.66-0.86)	0	0.44	
7-9		7	0.84 (0.75-0.95)	50.1	0.06		12	0.78 (0.71-0.86)	72.3	<0.0001	
Adjustment for confounding factors											
Age	Yes	8	0.85 (0.76-0.94)	41.8	0.10	NC	16	0.78 (0.72-0.84)	66.0	<0.0001	NC
	No	0					0				
Education	Yes	4	0.80 (0.64-1.01)	63.4	0.04	0.40	6	0.69 (0.55-0.88)	78.5	<0.0001	0.46
	No	4	0.89 (0.82-0.97)	0	0.50		10	0.81 (0.75-0.88)	50.7	0.03	
Family history of CHD	Yes	2	0.90 (0.83-0.99)	0	0.97	0.28	2	0.82 (0.78-0.87)	15.6	0.28	0.54
	No	6	0.80 (0.67-0.94)	48.6	0.08		14	0.75 (0.67-0.84)	69.7	<0.0001	
Body mass index	Yes	8	0.85 (0.76-0.94)	41.8	0.10	NC	13	0.78 (0.71-0.85)	71.2	<0.0001	0.82
	No	0					3	0.77 (0.66-0.90)	0	0.42	

Smoking	Yes	8	0.85 (0.76-0.94)	41.8	0.10	NC	15	0.76 (0.70-0.83)	58.0	0.003	0.20
	No	0					1	0.94 (0.86-1.02)			
Alcohol	Yes	8	0.85 (0.76-0.94)	41.8	0.10	NC	12	0.79 (0.72-0.86)	70.0	<0.0001	0.59
	No	0					4	0.68 (0.50-0.93)	53.4	0.09	
Physical activity	Yes	8	0.85 (0.76-0.94)	41.8	0.10	NC	13	0.77 (0.71-0.84)	72.3	<0.0001	0.69
	No	0					3	0.82 (0.66-1.01)	0	0.66	
Hypertension	Yes	8	0.85 (0.76-0.94)	41.8	0.10	NC	9	0.74 (0.68-0.82)	64.6	0.004	0.18
	No	0					7	0.85 (0.74-0.97)	52.4	0.05	
Hypercholesterolemia	Yes	5	0.89 (0.81-0.98)	25.8	0.25	0.18	5	0.81 (0.76-0.86)	29.0	0.23	0.90
	No	3	0.73 (0.57-0.95)	40.3	0.19		11	0.75 (0.65-0.87)	74.7	<0.0001	
Coffee	Yes	1	1.06 (0.79-1.32)			0.22	0				NC
	No	7	0.83 (0.75-0.92)	36.3	0.15		16	0.78 (0.72-0.84)	66.0	<0.0001	
Sugar-sweetened beverages	Yes	1	1.06 (0.79-1.32)			0.22	0				NC
	No	7	0.83 (0.75-0.92)	36.3	0.15		16	0.78 (0.72-0.84)	66.0	<0.0001	
Red or processed meat	Yes	7	0.87 (0.79-0.97)	33.3	0.17	0.21	11	0.79 (0.73-0.87)	72.6	<0.0001	0.53
	No	1	0.72 (0.57-0.90)				5	0.72 (0.62-0.85)	9.5	0.35	
Fish	Yes	4	0.78 (0.58-1.04)	63.2	0.04	0.78	7	0.73 (0.61-0.87)	80.9	<0.0001	0.62
	No	4	0.87 (0.79-0.95)	16.2	0.31		9	0.80 (0.74-0.86)	35.2	0.14	
Fruit and vegetables	Yes	8	0.85 (0.76-0.94)	41.8	0.10	NC	11	0.77 (0.70-0.84)	73.2	<0.0001	0.66
	No	0					5	0.81 (0.65-0.99)	38.7	0.16	
Whole grains	Yes	1	1.06 (0.79-1.32)			0.22	2	0.44 (0.07-2.58)	83.1	0.02	0.34
	No	7	0.83 (0.75-0.92)	36.3	0.15		14	0.77 (0.71-0.83)	54.2	0.008	
Dairy	Yes	2	0.71 (0.28-1.79)	79.6	0.03	0.51	3	0.44 (0.23-0.86)	55.6	0.11	0.008
	No	6	0.85 (0.78-0.92)	19.0	0.29		13	0.81 (0.76-0.86)	50.4	0.02	
Energy intake	Yes	8	0.85 (0.76-0.94)	41.8	0.10	NC	11	0.76 (0.69-0.83)	74.5	<0.0001	0.43
	No	0					5	0.84 (0.74-0.95)	16.4	0.31	

*n*denotes the number of studies.
[1] P for heterogeneity within each subgroup,
[2] P for heterogeneity between subgroups with meta-regression analysis,
[3] P for heterogeneity between men and women (studies with genders mixed were excluded),
NC = not calculable

Supplementary Table 18. Newcastle-Ottawa score for studies included in the dose-response analysis of nut consumption and coronary heart disease

Author, publication year	Represen-tativeness	Selection of non-exposed cohort	Exposure-ascertainment[1]	Demonstration of outcome not present at start[2]	Adjustment for age	Adjustment for any other factor	Assess-ment of outcome	Long enough follow-up[3]	Adequacy of follow-up[4]	Total score
Fraser, 1992	0	1	1	1	1	1	1	1	1	8
Mann, 1997	0	1	0	1	1	1	1	1	0	6
Albert, 2002	0	1	1	1	1	1	1	1	0	7
Blomhoff, 2006	1	1	0	1	1	1	1	1	0	7
Bao, 2013, HPFS	0	1	1	1	1	1	1	1	1	8
Bao, 2013, NHS	0	1	1	1	1	1	1	1	1	8
Haring, 2014	1	1	1	1	1	1	1	1	0	8
Van den Brandt, 2015	1	1	1	1	1	1	1	1	1	9
Luu, 2015	1	1	1	0	1	1	1	1	1	8
Hshieh, 2015	0	1	1	0	1	1	1	1	0	6
Gopinath, 2015	1	1	1	0	1	1	1	1	0	7
Wang, 2016	0	1	0	0	1	1	1	1	0	5

[1] 1 point for validated self-reported questionnaires or interview
[2] 1 point for excluding prevalent coronary heart disease or cardiovascular disease cases
[3] 1 point for loss-to-follow-up less than 10%
[4] 1 point for 3 years or more

Supplementary Table 19. Newcastle-Ottawa score for studies included in the dose-response analysis of nut consumption and stroke

Author, publication year	Represen-tativeness	Selection of non-exposed cohort	Exposure-ascertainment[1]	Demonstration of outcome not present at start[2]	Adjustment for age	Adjustment for any other factor	Assess-ment of outcome	Long enough follow-up[3]	Adequacy of follow-up[4]	Total score
Yochum, 2000	1	1	1	1	1	1	1	1	0	8
Djousse, 2010	0	1	1	1	1	1	1	1	0	7
Yaemsiri, 2012	1	1	1	1	1	1	1	1	0	8
Bernstein, 2012, HPFS	0	1	1	1	1	1	1	1	1	8
Bernstein, 2012, NHS	0	1	1	1	1	1	1	1	1	8
Di Giuseppe, 2014	1	1	1	1	1	1	1	1	1	9
Van den Brandt, 2015	1	1	1	1	1	1	1	1	1	9
Luu, 2015	1	1	1	0	1	1	1	1	1	8
Haring, 2015	1	1	1	1	1	1	1	1	0	8
Gopinath, 2015	1	1	1	0	1	1	1	1	0	7
Wang, 2016	0	1	0	0	1	1	1	1	0	5

[1] 1 point for validated self-reported questionnaires or interview
[2] 1 point for excluding prevalent stroke or cardiovascular disease cases
[3] 1 point for loss-to-follow-up less than 10%
[4] 1 point for 3 years or more

Supplementary Table 20. Newcastle-Ottawa score for studies included in the dose-response analysis of nut consumption and cardiovascular disease

Author, publication year	Represen-tativeness	Selection of non-exposed cohort	Exposure-ascertainment[1]	Demonstration of outcome not present at start[2]	Adjustment for age	Adjustment for any other factor	Assess-ment of outcome	Long enough follow-up[3]	Adequacy of follow-up[4]	Total score
Blomhoff, 2006	1	1	0	1	1	1	1	1	0	7
Fitzgerald, 2012	1	1	1	1	1	1	1	1	1	9
Von Ruesten, 2013	1	1	1	1	1	1	1	1	1	9
Guasch-Ferre, 2013	0	1	1	1	1	1	1	1	0	7
Bao, 2013, HPFS	0	1	1	1	1	1	1	1	1	8
Bao, 2013, NHS	0	1	1	1	1	1	1	1	1	8
Van den Brandt, 2015	1	1	1	1	1	1	1	1	1	9
Luu, 2015	1	1	1	0	1	1	1	1	1	8
Hshieh, 2015	0	1	1	0	1	1	1	1	0	6
Gopinath, 2015	1	1	1	0	1	1	1	1	0	7
Wang, 2016	0	1	0	0	1	1	1	1	0	5
Eslemparast, 2016	1	1	1	0	1	1	1	1	1	8

[1] 1 point for validated self-reported questionnaires or interview
[2] 1 point for excluding prevalent coronary heart disease, stroke or cardiovascular disease cases
[3] 1 point for loss-to-follow-up less than 10%
[4] 1 point for 3 years or more

Supplementary Table 21. Newcastle-Ottawa score for studies included in the dose-response analysis of nut consumption and total cancer

Author, publication year	Represen-tativeness	Selection of non-exposed cohort	Exposure-ascertainment[1]	Demonstration of outcome not present at start[2]	Adjustment for age	Adjustment for any other factor	Assess-ment of outcome	Long enough follow-up[3]	Adequacy of follow-up[4]	Total score
Von Ruesten, 2013	1	1	1	1	1	1	1	1	1	9
Guasch-Ferre, 2013	0	1	1	1	1	1	1	1	0	7
Bao, 2013, HPFS	0	1	1	1	1	1	1	1	1	8
Bao, 2013, NHS	0	1	1	1	1	1	1	1	1	8
Van den Brandt, 2015	1	1	1	1	1	1	1	1	1	9
Luu, 2015	1	1	1	1	1	1	1	1	1	9
Hshieh, 2015	0	1	1	0	1	1	1	1	0	6
Eslemparast, 2016	1	1	1	0	1	1	1	1	1	8

[1] 1 point for validated self-reported questionnaires or interview
[2] 1 point for excluding prevalent cancer cases
[3] 1 point for loss-to-follow-up less than 10%
[4] 1 point for 3 years or more

Supplementary Table 22. Newcastle-Ottawa score for studies included in the dose-response analysis of nut consumption and all-cause mortality

Author, publication year	Representativeness	Selection of non-exposed cohort	Exposure-ascertainment[1]	Demonstration of outcome not present at start[2]	Adjustment for age	Adjustment for any other factor	Assessment of outcome	Long enough follow-up[3]	Adequacy of follow-up[4]	Total score
Mann, 1997	0	1	0	1	1	1	1	1	0	6
Fraser, 1997, whites	0	1	0	1	1	1	1	1	1	7
Fraser, 1997, blacks	0	1	0	0	1	1	1	1	1	6
Blomhoff, 2006	1	1	0	1	1	1	1	1	0	7
Leenders, 2013	1	1	1	1	1	1	1	1	1	9
Guasch-Ferre, 2013	0	1	1	1	1	1	1	1	0	7
Bao, 2013, HPFS	0	1	1	1	1	1	1	1	1	8
Bao, 2013, NHS	0	1	1	1	1	1	1	1	1	8
Fernandez-Montero, 2014	0	1	1	0	1	1	1	1	1	7
Van den Brandt, 2015	1	1	1	1	1	1	1	1	1	9
Luu, 2015	1	1	1	1	1	1	1	1	1	9
Hshieh, 2015	0	1	1	0	1	1	1	1	0	6
Gopinath, 2015	1	1	1	0	1	1	1	1	0	7
Bonaccio, 2015	1	1	1	1	1	1	1	1	1	9

Wang, 2016	0	1	0	0	1	1	1	1	0	5
Eslemparast, 2016	1	1	1	0	1	1	1	1	1	8

[1] 1 point for validated self-reported questionnaires or interview
[2] 1 point for excluding prevalent cardiovascular disease or cancer cases
[3] 1 point for loss-to-follow-up less than 10%
[4] 1 point for 3 years or more

Supplementary Table 23. Attributable fractions and number of deaths due to coronary heart disease, cancer, respiratory disease, diabetes, and total mortality by country attributable to a nut intake below 20 g/d in North and South America, Europe, Southeast Asia and the Western Pacific

		Coronary heart disease		Total cancer		Respiratory disease		Diabetes		All-cause mortality	
		20 g/d		20 g/d		20 g/d		20 g/d		20 g/d	
Region	Country	%	N	%	N	%	N	%	N	%	N
America A	Canada	13.3	7087	5.2	4096	21.6	3349	11.7	951	8.6	23191
	Cuba	13.3	2958	5.2	1200	21.6	894	11.7	243	8.6	8195
	United States	13.3	71718	5.2	32872	21.6	39338	11.7	8762	8.6	227203
America B	Antigua and Barbuda	18.8	16	7.4	7	27.9	4	13.5	7	11.4	62
	Argentina	18.8	9932	7.4	5241	27.9	9627	13.5	1101	11.4	37016
	Barbados	18.8	63	7.4	33	27.9	18	13.5	33	11.4	265
	Belize	18.8	46	7.4	21	27.9	17	13.5	20	11.4	200
	Brazil	18.8	34231	7.4	15834	27.9	20286	13.5	6541	11.4	136313
	Chile	18.8	2223	7.4	1979	27.9	1789	13.5	367	11.4	12200
	Colombia	18.8	6906	7.4	3223	27.9	4888	13.5	985	11.4	26080
	Costa Rica	18.8	580	7.4	362	27.9	393	13.5	53	11.4	2348
	Dominica	18.8	11	7.4	6	27.9	5	13.5	6	11.4	55
	Dominican Republic	18.8	2183	7.4	673	27.9	665	13.5	295	11.4	6462
	El Salvador	18.8	1282	7.4	353	27.9	606	13.5	215	11.4	4328
	Grenada	18.8	25	7.4	10	27.9	7	13.5	10	11.4	94
	Guyana	18.8	167	7.4	53	27.9	33	13.5	55	11.4	606
	Honduras	18.8	1410	7.4	323	27.9	978	13.5	132	11.4	4296
	Jamaica	18.8	395	7.4	241	27.9	198	13.5	272	11.4	1995
	Mexico	18.8	15007	7.4	6189	27.9	10584	13.5	6844	11.4	69940
	Panama	18.8	448	7.4	245	27.9	277	13.5	110	11.4	2031
	Paraguay	18.8	999	7.4	426	27.9	320	13.5	274	11.4	3701
	Saint Lucia	18.8	34	7.4	17	27.9	14	13.5	17	11.4	153
	Saint Vincent and the Grenadines	18.8	23	7.4	9	27.9	5	13.5	8	11.4	82
	Suriname	18.8	94	7.4	44	27.9	31	13.5	30	11.4	416
	The Bahamas	18.8	78	7.4	38	27.9	21	13.5	28	11.4	317
	Trinidad and Tobago	18.8	346	7.4	134	27.9	92	13.5	209	11.4	1343
	Uruguay	18.8	859	7.4	643	27.9	619	13.5	91	11.4	3778

	Venezuela	18.8	4385	7.4	1960	27.9	1836	13.5	905	11.4	16828
America D	Bolivia	18.8	1168	7.4	553	27.9	605	13.5	122	11.4	5105
	Ecuador	18.8	1955	7.4	1018	27.9	962	13.5	455	11.4	8867
	Guatemala	18.8	1287	7.4	607	27.9	753	13.5	482	11.4	7389
	Haiti	18.8	1109	7.4	498	27.9	355	13.5	422	11.4	64687318
	Nicaragua	18.8	693	7.4	220	27.9	370	13.5	154	11.4	2439
	Peru	18.8	3008	7.4	1891	27.9	2278	13.5	429	11.4	14295
Europa A	Andorra	16.5	21	6.0	11	26.2	10	13.2	1	10.3	67
	Austria	16.5	3658	6.0	1311	26.2	907	13.2	328	10.3	8709
	Belgium	16.5	3385	6.0	1780	26.2	2041	13.2	252	10.3	11698
	Croatia	16.5	2181	6.0	777	26.2	680	13.2	132	10.3	5325
	Czech Republic	16.5	5229	6.0	1706	26.2	1085	13.2	268	10.3	11502
	Denmark	17.1	1526	6.3	875	26.9	1179	13.9	194	10.7	5760
	Finland	16.5	2029	6.0	726	26.2	456	13.2	54	10.3	5445
	France	16.0	14881	5.8	10454	25.4	6353	12.8	1620	9.9	58477
	Germany	16.7	37138	6.0	14470	26.4	12149	13.4	2770	10.4	95450
	Greece	17.2	6573	6.3	2033	27.1	2490	14.0	221	10.8	14647
	Iceland	16.5	65	6.0	33	26.2	26	13.2	3	10.3	197
	Ireland	16.5	985	6.0	507	26.2	561	13.2	66	10.3	3166
	Israel	16.5	1190	6.0	653	26.2	494	13.2	297	10.3	4372
	Italy	17.4	19120	6.4	11464	27.5	8104	14.2	2980	11.0	69682
	Luxembourg	16.5	135	6.0	69	26.2	63	13.2	7	10.3	408
	Malta	16.5	127	6.0	47	26.2	35	13.2	11	10.3	292
	Netherlands	13.4	3174	4.4	2195	21.4	2105	9.7	324	8.0	12071
	Norway	16.2	1232	5.9	664	25.7	667	13.0	91	10.1	4252
	Portugal	16.5	2655	6.0	1653	26.2	1572	13.2	534	10.3	11288
	Slovenia	16.5	589	6.0	333	26.2	278	13.2	47	10.3	2157
	Spain	15.6	11868	5.5	6280	24.9	7309	12.2	1276	9.7	41093
	Sweden	19.5	4452	6.8	1565	29.8	1280	15.2	317	12.1	11285
	Switzerland	16.5	2217	6.0	1103	26.2	689	13.2	214	10.3	6695
	United Kingdom	19.5	18457	6.8	11083	29.8	12520	15.2	901	12.1	70049
Europe B	Albania	16.5	717	6.0	255	26.2	373	13.2	30	10.3	2431
	Armenia	16.5	1448	6.0	320	26.2	339	13.2	168	10.3	2854
	Azerbaijan	16.5	3008	6.0	430	26.2	588	13.2	169	10.3	5502
	Bosnia and Herzegovina	16.5	1065	6.0	484	26.2	572	13.2	268	10.3	3719
	Bulgaria	16.5	4680	6.0	1037	26.2	2465	13.2	252	10.3	11288

	Georgia	16.5	2885	6.0	367	26.2	1053	13.2	109	10.3	5457
	Kyrgyzstan	16.5	1662	6.0	214	26.2	589	13.2	37	10.3	3443
	Macedonia	16.5	524	6.0	212	26.2	185	13.2	93	10.3	1848
	Montenegro	16.5	215	6.0	71	26.2	12	13.2	18	10.3	630
	Poland	16.5	13048	6.0	5745	26.2	6188	13.2	879	10.3	40811
	Romania	16.5	10877	6.0	2485	26.2	2228	13.2	258	10.3	24538
	Serbia	16.5	2479	6.0	1168	26.2	1143	13.2	362	10.3	9482
	Slovakia	16.5	3163	6.0	740	26.2	345	13.2	91	10.3	5473
	Tajikistan	16.5	1519	6.0	197	26.2	422	13.2	84	10.3	3237
	Turkey	16.5	11025	6.0	5534	26.2	10559	13.2	2535	10.3	39610
	Turkmenistan	16.5	1713	6.0	223	26.2	364	13.2	64	10.3	3059
	Uzbekistan	16.5	8958	6.0	852	26.2	1723	13.2	468	10.3	16326
Europe C	Belarus	16.5	11134	6.0	1273	26.2	1227	13.2	70	10.3	14950
	Estonia	16.5	958	6.0	202	26.2	80	13.2	18	10.3	1626
	Hungary	16.5	6066	6.0	1922	26.2	1536	13.2	300	10.3	12979
	Kazakhstan	16.5	7281	6.0	1318	26.2	1648	13.2	190	10.3	15001
	Latvia	16.5	1672	6.0	309	26.2	121	13.2	42	10.3	2759
	Lithuania	16.5	2797	6.0	436	26.2	268	13.2	28	10.3	4016
	Moldova	16.5	2785	6.0	320	26.2	331	13.2	31	10.3	3998
	Russia	16.5	111226	6.0	17385	26.2	9107	13.2	1199	10.3	189142
	Ukraine	16.5	57197	6.0	5420	26.2	4361	13.2	304	10.3	68634
South-East Asia B	Indonesia	17.9	33518	6.0	8698	27.7	35280	13.4	10584	10.9	154589
	Sri Lanka	17.9	4126	6.0	552	27.7	4455	13.4	1431	10.9	13760
	Thailand	17.9	11297	6.0	5720	27.7	10147	13.4	3246	10.9	55079
	Timor-Leste	17.9	125	6.0	23	27.7	72	13.4	14	10.9	359
South-East Asia D	Bangladesh	17.9	12690	6.0	6518	27.7	24887	13.4	3900	10.9	89654
	Bhutan	17.9	89	6.0	22	27.7	115	13.4	24	10.9	424
	India	17.9	222354	6.0	39138	27.7	384446	13.4	31769	10.9	961811
	Maldives	17.9	21	6.0	5	27.7	29	13.4	4	10.9	103
	Myanmar	17.9	4862	6.0	3897	27.7	9763	13.4	1815	10.9	42922
	Nepal	17.9	3203	6.0	766	27.7	4313	13.4	588	10.9	14469
	South Korea	15.5	6290	6.0	6035	24.8	5784	13.4	2285	10.0	34832
Western Pacific A	Australia	13.4	3734	4.0	1921	21.2	2287	8.9	403	7.8	12734
	Brunei	18.3	37	6.1	17	28.2	27	13.8	20	11.2	145
	Japan	18.3	26562	6.1	25537	28.2	26469	13.8	1319	11.2	149548
	New Zealand	18.3	1105	6.1	588	28.2	639	13.8	101	7.8	2557
	Singapore	18.3	804	6.1	379	28.2	308	13.8	70	11.2	2617

Western Pacific B	Cambodia	19.5	3802	6.9	744	29.4	2500	14.7	502	12.0	12182
	China	19.5	232554	6.9	145187	29.4	313550	14.7	19280	12.0	1066120
	Federated States of Micronesia	19.5	16	6.9	3	29.4	18	14.7	17	12.0	83
	Fiji	19.5	238	6.9	45	29.4	124	14.7	235	12.0	829
	Kiribati	19.5	13	6.9	3	29.4	21	14.7	18	12.0	97
	Laos	19.5	1078	6.9	260	29.4	820	14.7	202	12.0	4106
	Malaysia	19.5	4070	6.9	1455	29.4	2589	14.7	633	12.0	16917
	Marshall Islands	19.5	10	6.9	2	29.4	10	14.7	12	12.0	53
	Mongolia	19.5	651	6.9	301	29.4	120	14.7	17	12.0	2344
	North Korea	19.5	7793	6.9	3246	29.4	6107	14.7	455	12.0	24490
	Oceania	19.5	2092	6.9	459	29.4	1945	14.7	1006	12.0	8269
	Papua New Guinea	19.5	1648	6.9	371	29.4	1574	14.7	581	12.0	6352
	Philippines	19.5	13004	6.9	4603	29.4	8336	14.7	3250	12.0	55203
	Samoa	19.5	33	6.9	7	29.4	27	14.7	28	12.0	128
	Solomon Islands	19.5	81	6.9	14	29.4	104	14.7	72	12.0	443
	Taiwan	19.5	2519	6.9	3162	29.4	3221	14.7	1564	12.0	19515
	Tonga	19.5	16	6.9	7	29.4	17	14.7	14	12.0	82
	Vanuatu	19.5	38	6.9	7	29.4	50	14.7	30	12.0	201
	Vietnam	19.5	6599	6.9	7789	29.4	9129	14.7	1809	12.0	60785
Regional	America A	13.3	81763	5.2	38168	21.6	43581	11.7	9956	8.6	258589
	America B	18.8	81743	7.4	38064	27.9	53313	11.7	18608	11.4	330909
	America D	18.8	9220	7.4	4787	27.9	5323	11.7	2064	11.4	45413
	Europe A	16.9	142887	6.0	71792	26.7	63053	13.4	12908	10.5	454087
	Europe B	16.5	68986	6.0	20335	26.2	27425	13.2	5417	10.3	179708
	Europe C	16.5	201116	6.0	28586	26.2	18679	13.2	2182	10.3	313105
	South-East Asia B	17.9	49066	6.0	14993	27.7	49954	13.4	15275	10.9	223787
	South-East Asia D	17.9	249509	6.0	56381	27.7	429337	13.4	40385	10.9	1144215
	Western Pacific A	17.6	32242	5.9	28442	27.5	29730	12.3	1913	10.8	167601
	Western Pacific B	19.5	276255	6.9	167665	29.4	350262	14.7	29725	12.0	1278200
Total (regions covered)		17.4	1192787	6.3	469213	27.8	1072380	13.2	138901	11.0	4395614

Supplementary Figure 1. Nuts and coronary heart disease, high vs. low analysis

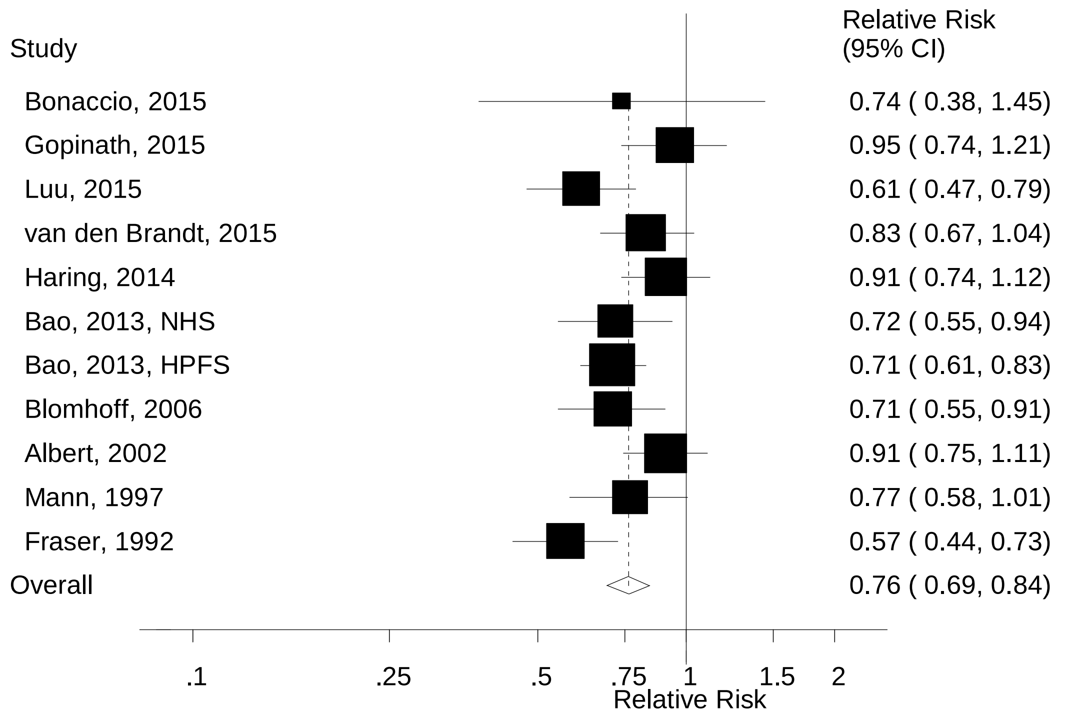

Supplementary Figure 2. Nuts and stroke, high vs. low analysis

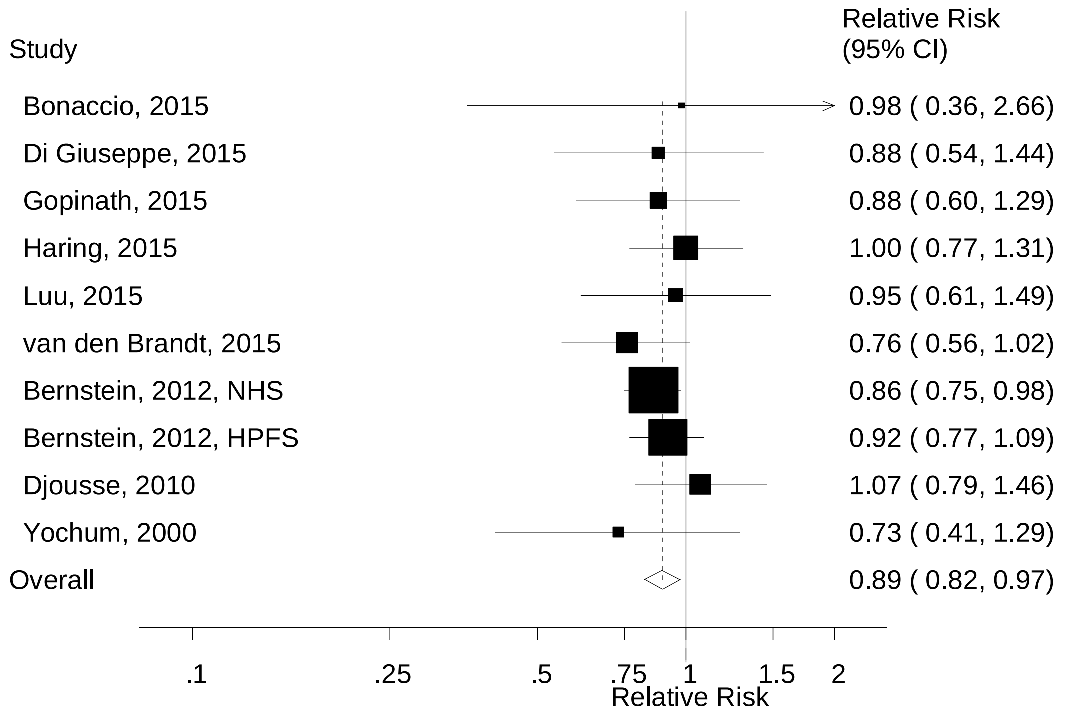

Supplementary Figure 3. Nuts and cardiovascular disease, high vs. low analysis

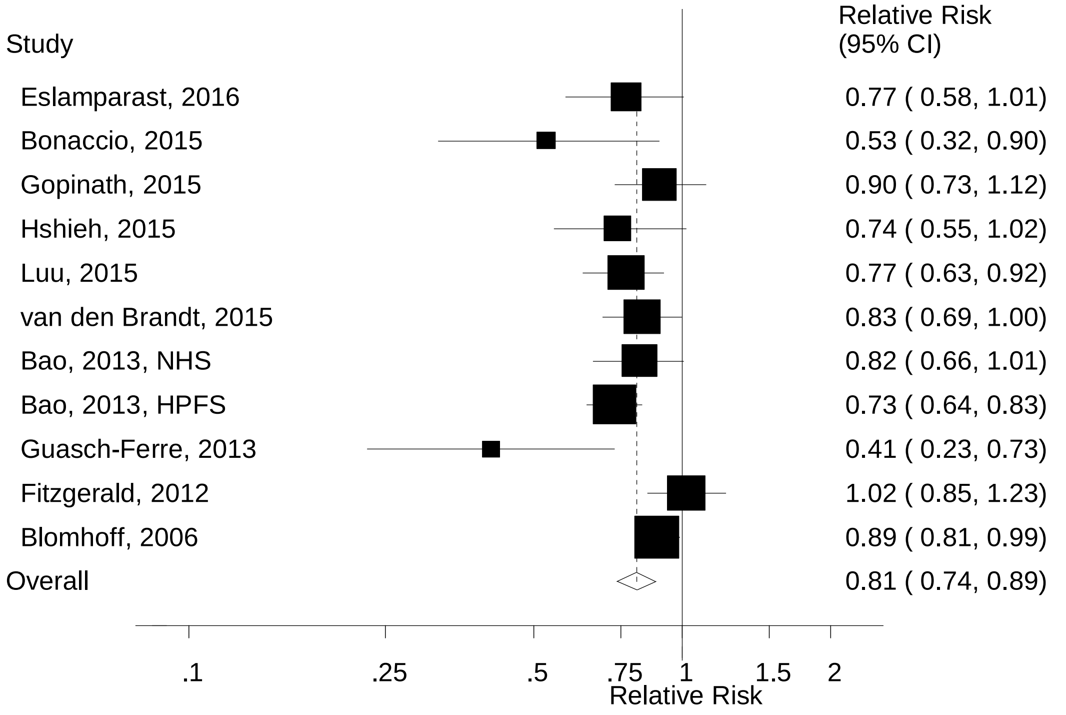

Supplementary Figure 4. Nuts and total cancer, high vs. low analysis

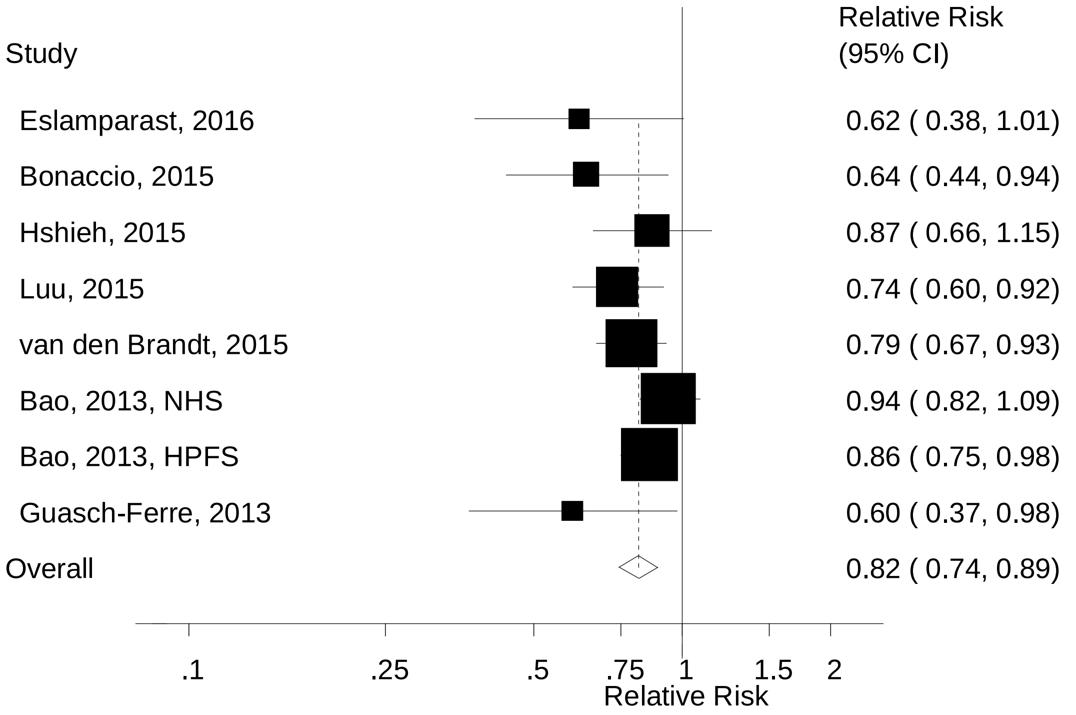

Supplementary Figure 5. Nuts and all-cause mortality, high vs. low analysis

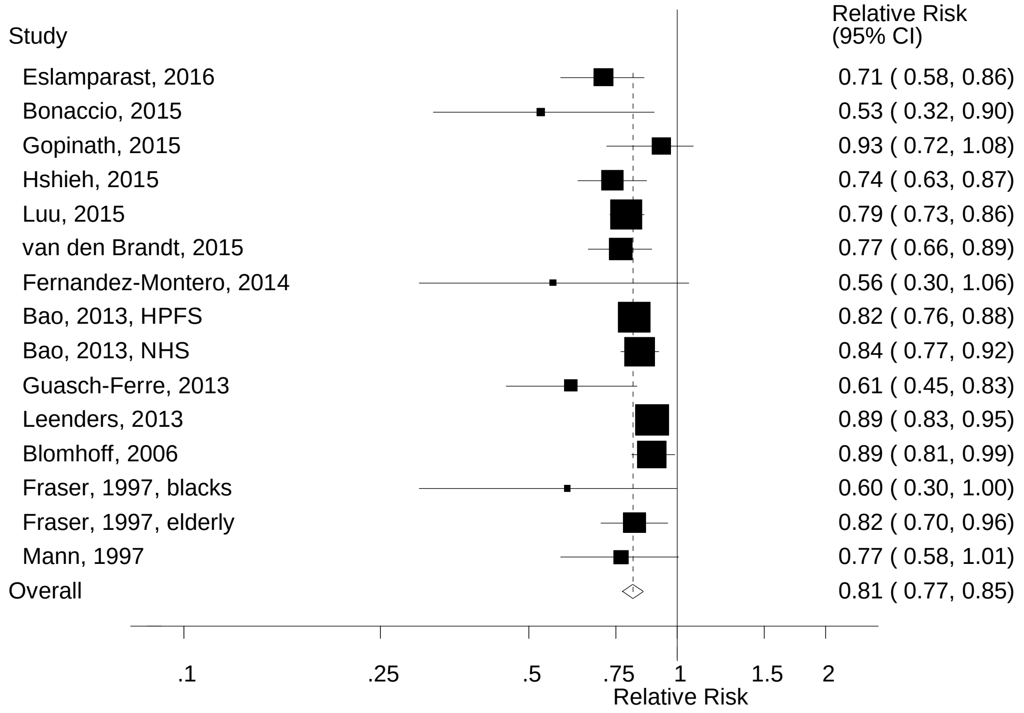

Supplementary Figure 6. Nuts and respiratory disease mortality, high vs. low analysis

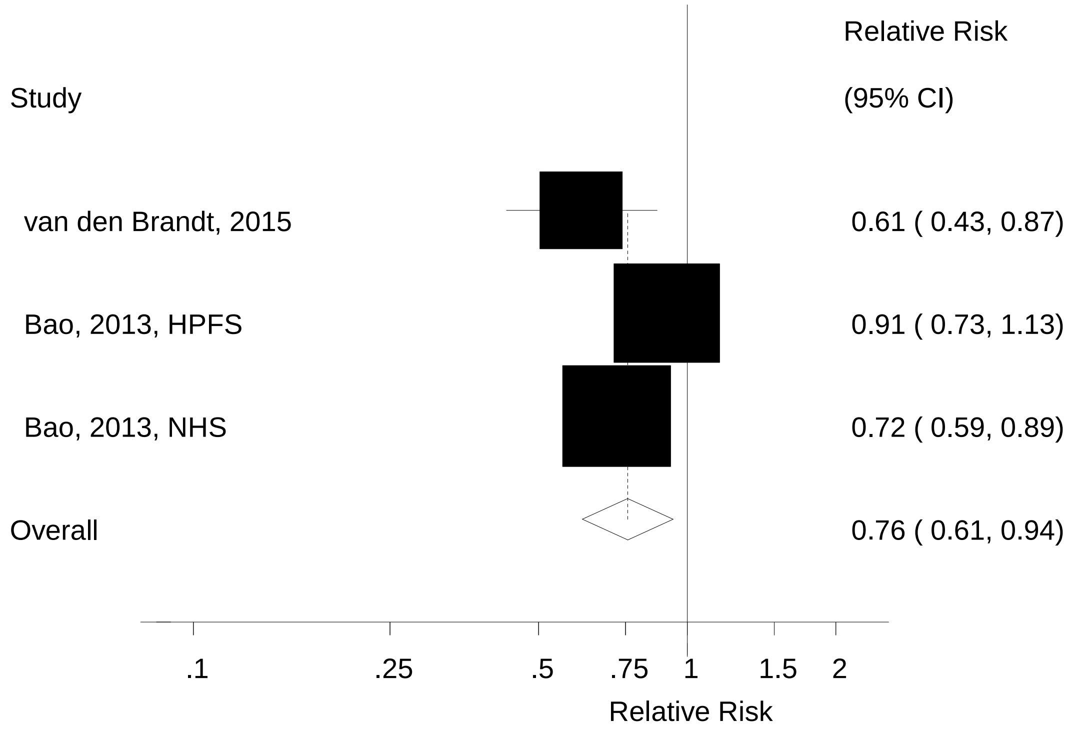

Supplementary Figure 7. Nuts and diabetes mortality, high vs. low analysis

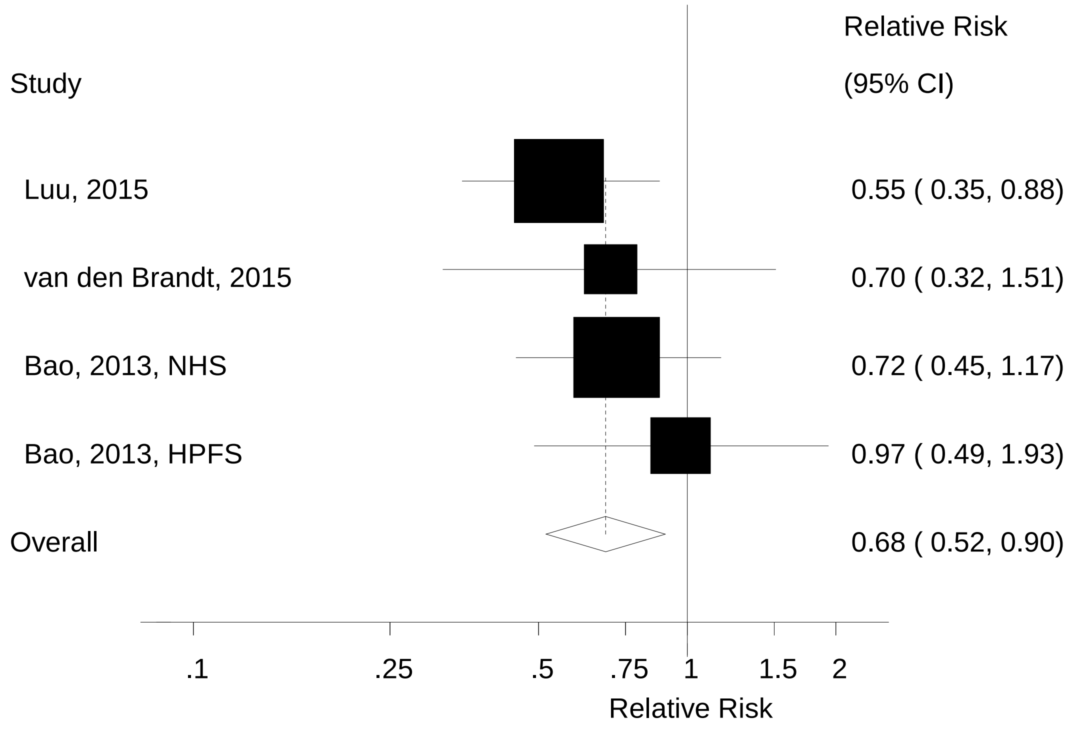

Supplementary Figure 8. Nuts and neurodegenerative disease mortality, high vs. low analysis

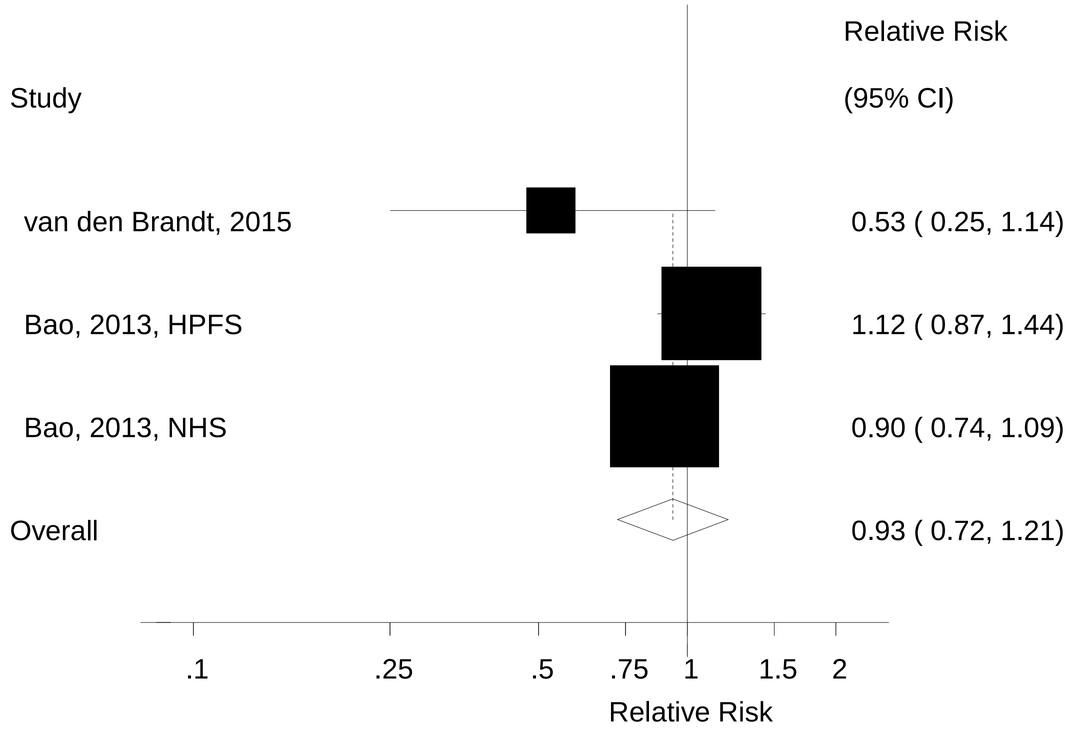

Supplementary Figure 9. Nuts and infectious disease mortality, high vs. low analysis

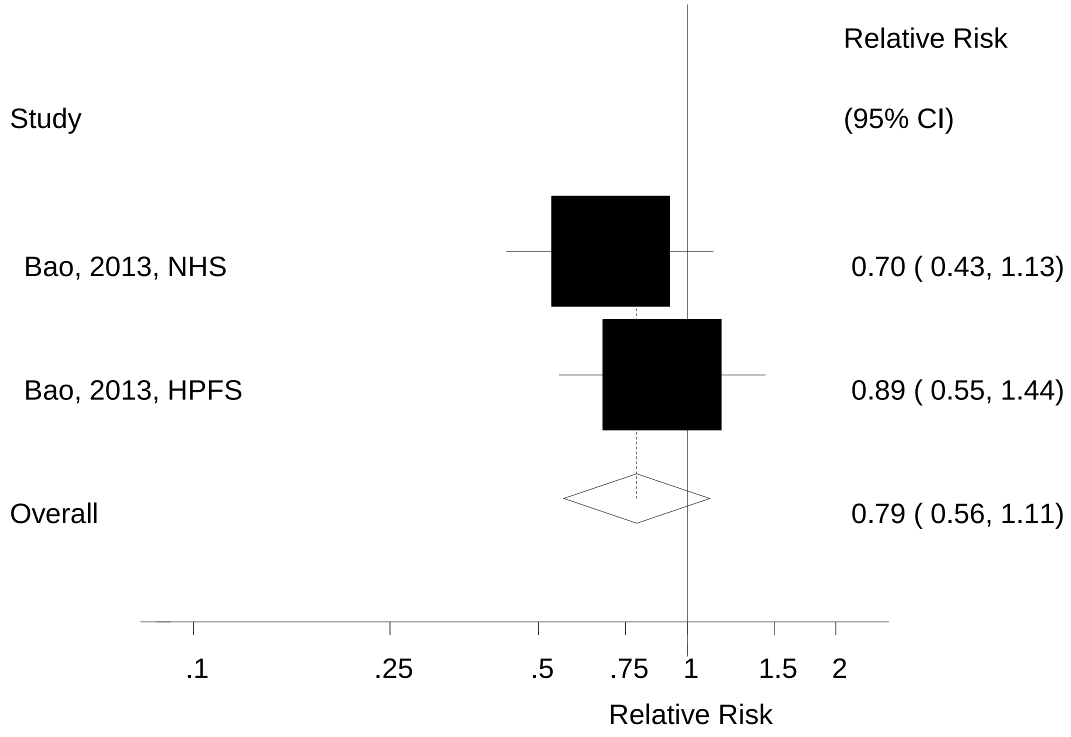

Supplementary Figure 10. Nuts and kidney disease mortality, high vs. low analysis

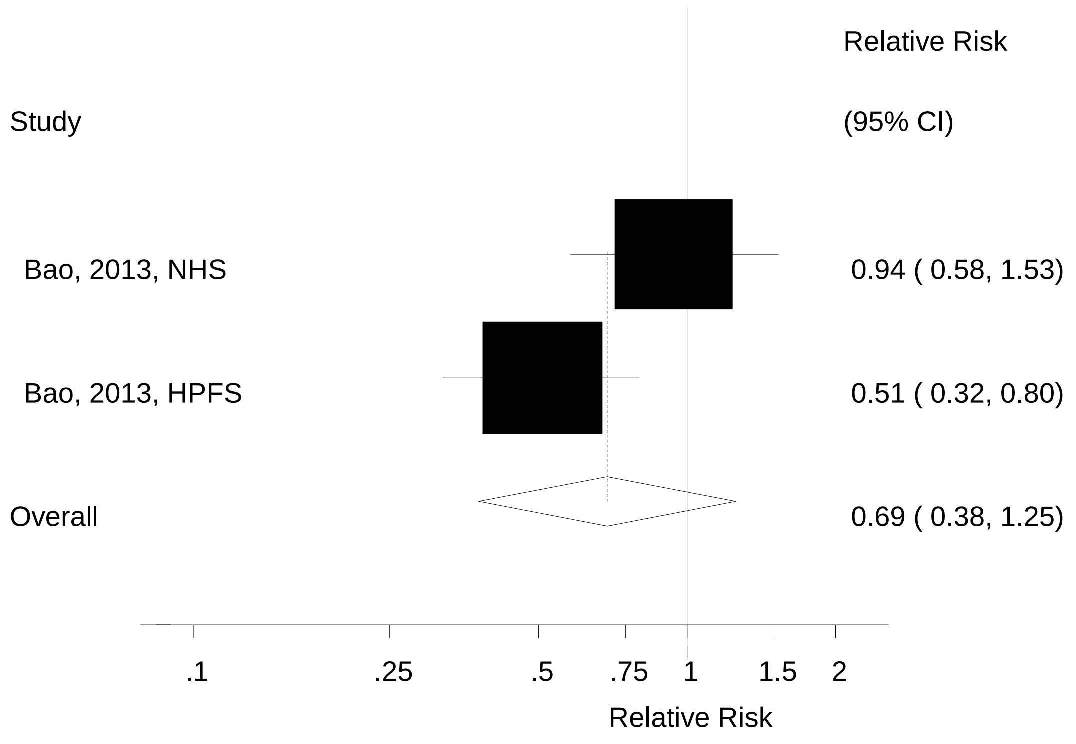

Supplementary Figure 11. Tree nuts and coronary heart disease, high vs. low analysis

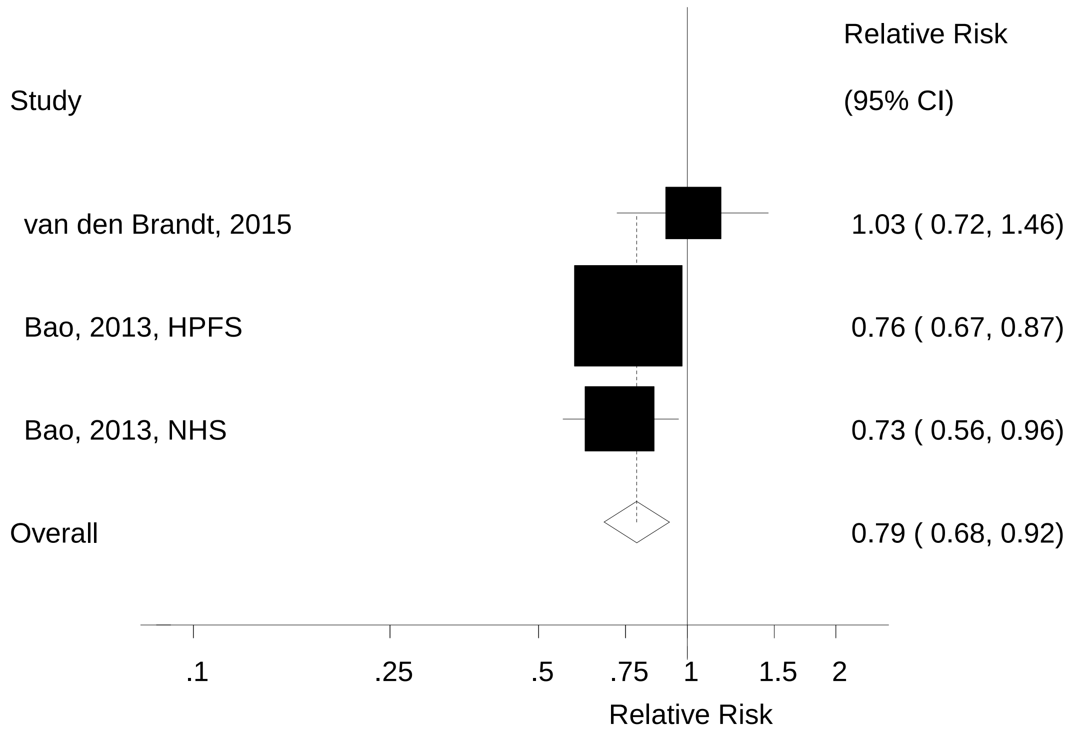

Supplementary Figure 12. Tree nuts and coronary heart disease, dose-response analysis, per 10 g/d

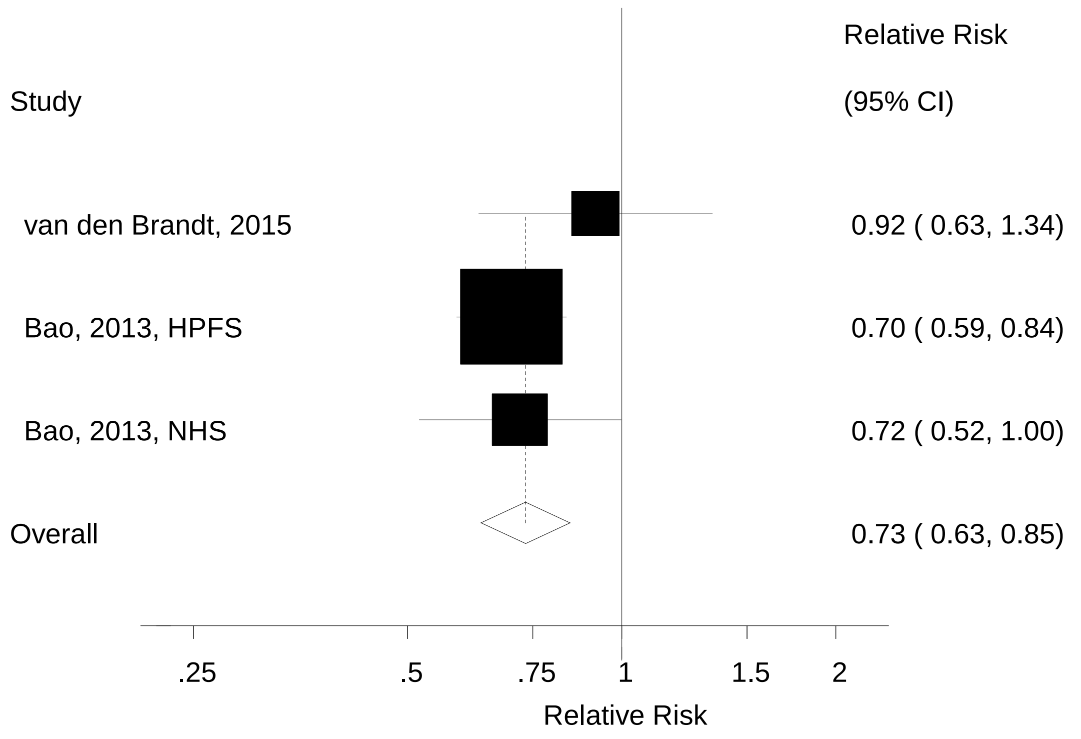

Supplementary Figure 13. Tree nuts and coronary heart disease, nonlinear dose-response analysis

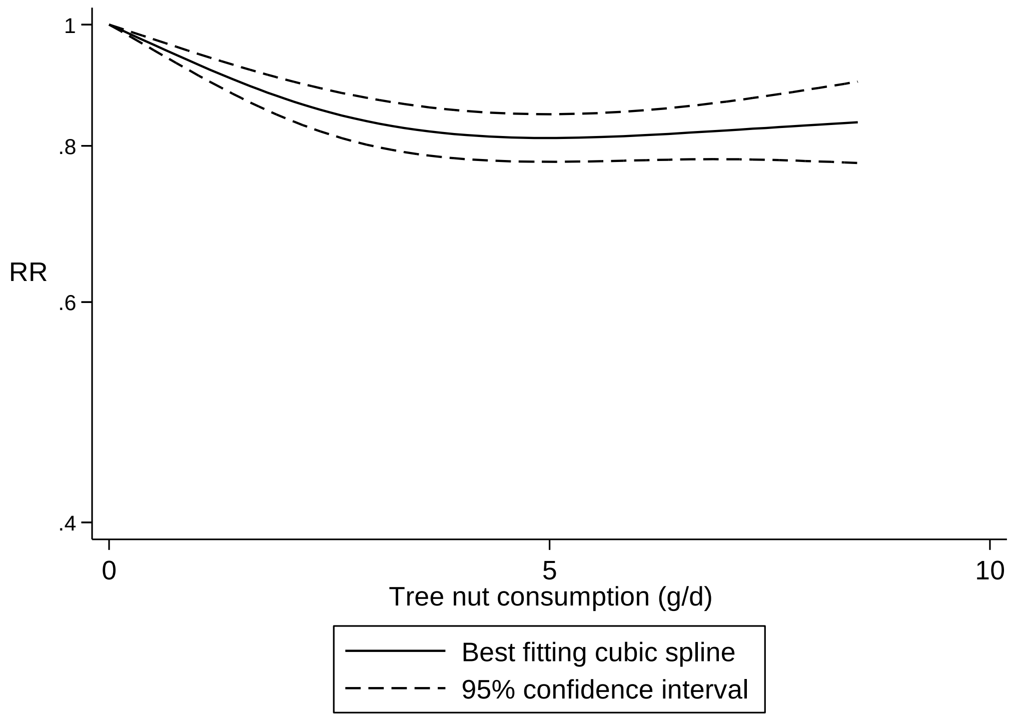

$p_{nonlinearity}$<0.0001

Supplementary Figure 14. Peanuts and coronary heart disease, high vs. low analysis

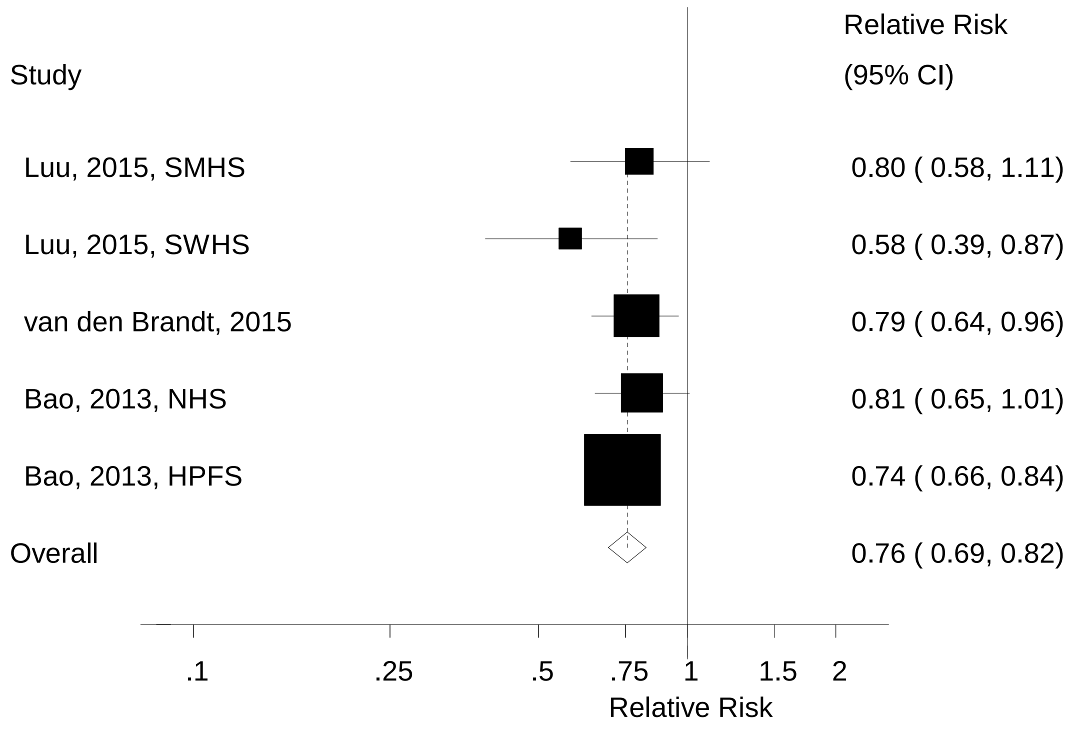

Supplementary Figure 15. Peanuts and coronary heart disease, dose-response analysis, per 10 g/d

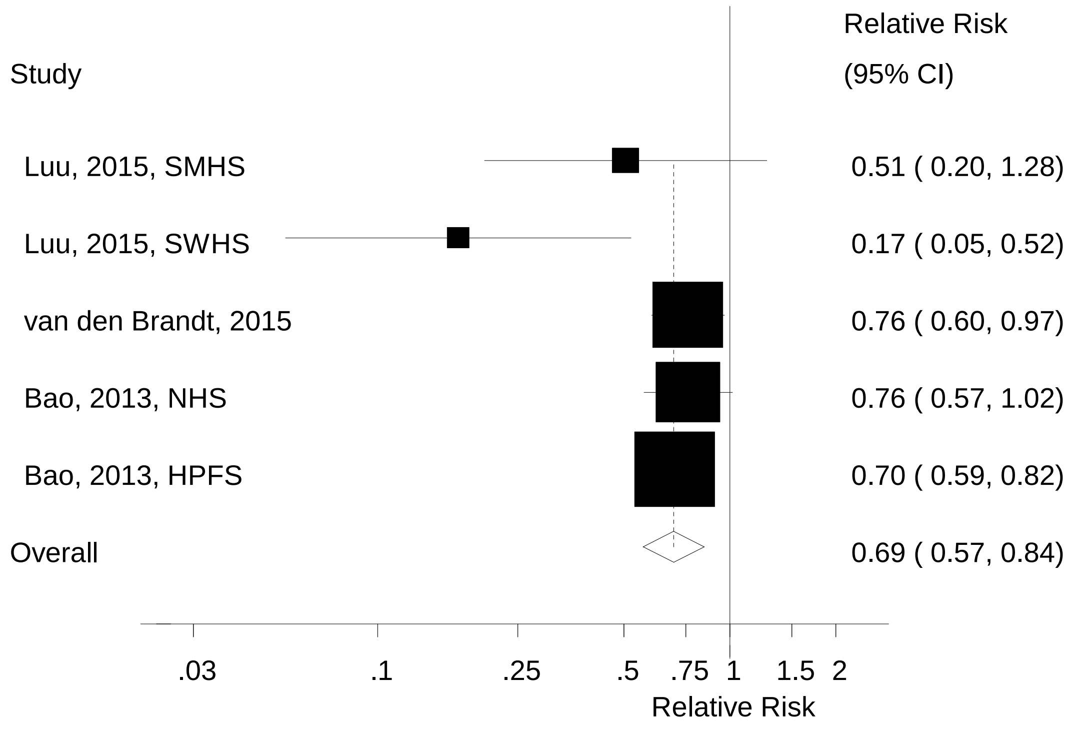

Supplementary Figure 16. Peanuts and coronary heart disease, nonlinear dose-response analysis

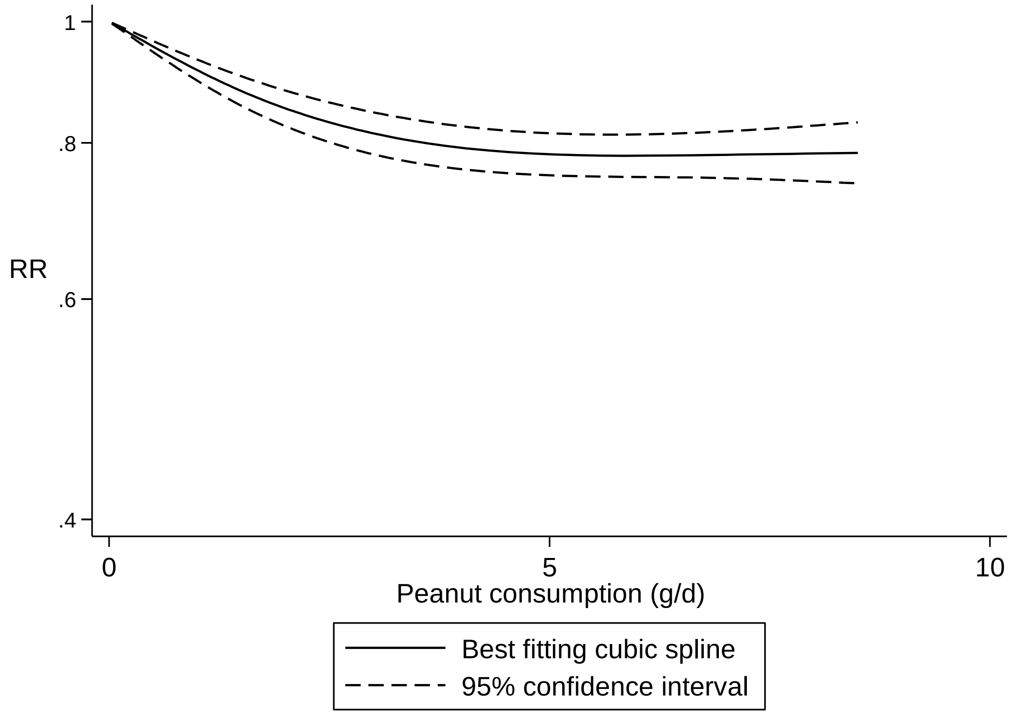

$p_{nonlinearity}$<0.0001

Supplementary Figure 17. Tree nuts and stroke, high vs. low analysis

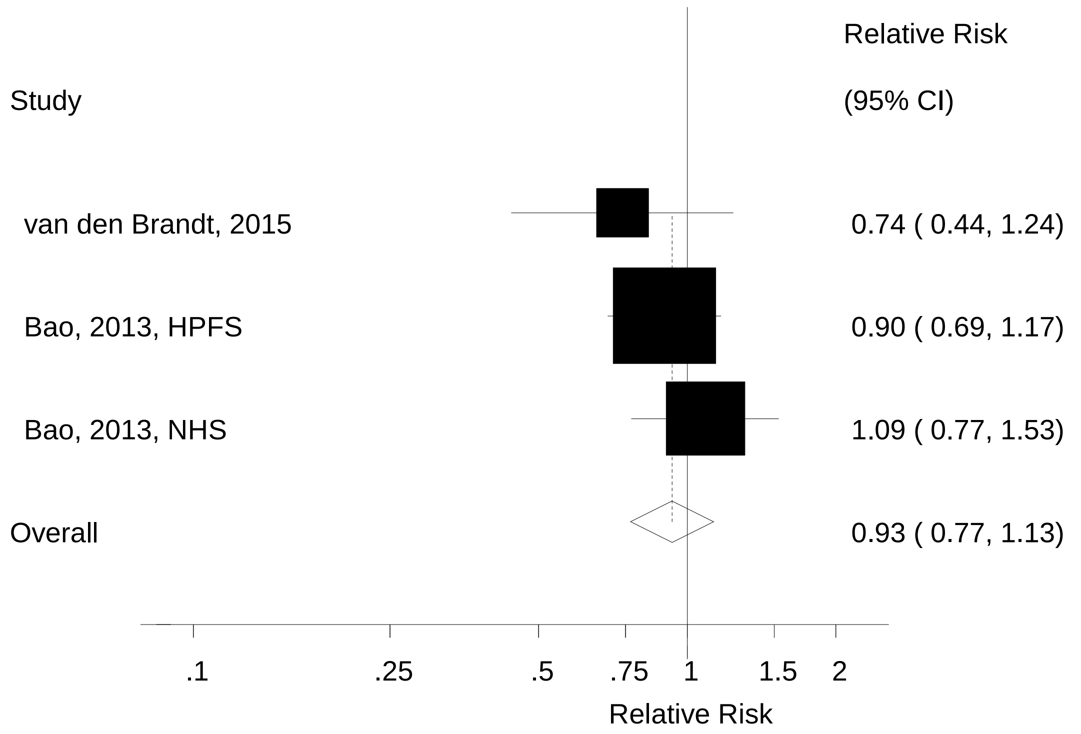

Supplementary Figure 18. Tree nuts and stroke, dose-response analysis, per 10 g/d

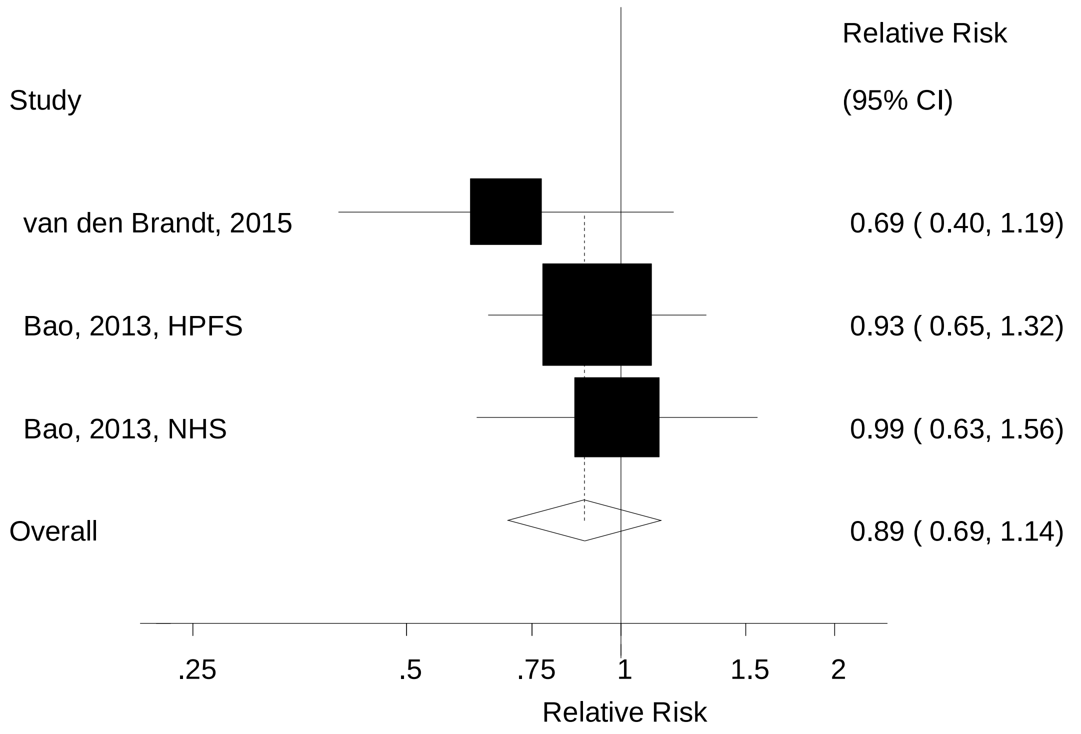

Supplementary Figure 19. Tree nuts and stroke, nonlinear dose-response analysis

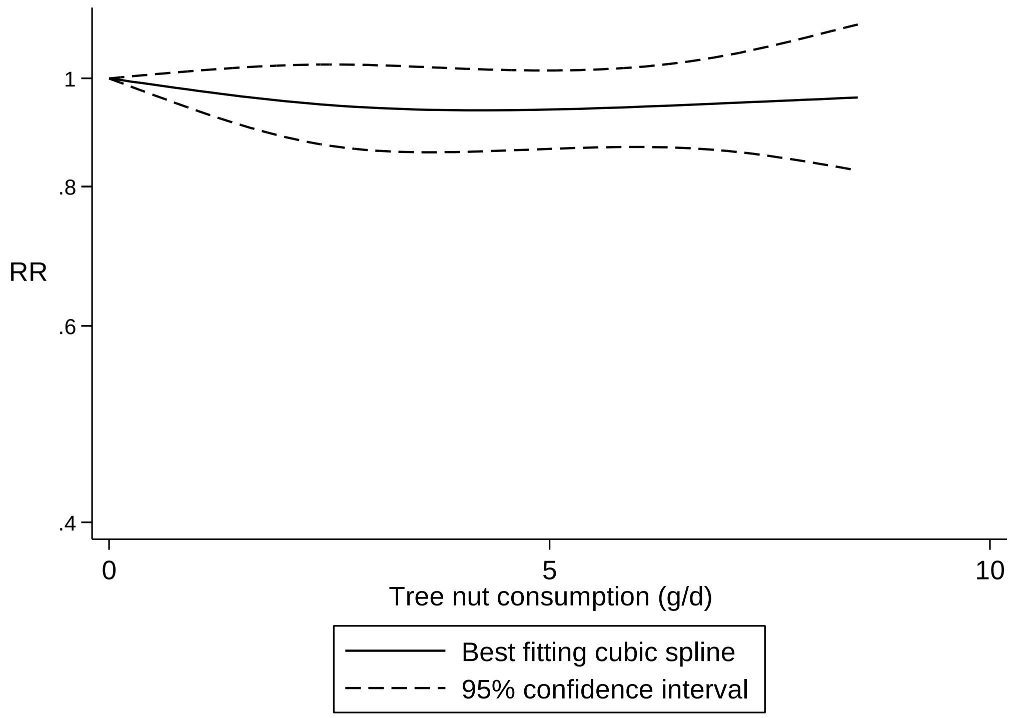

$p_{nonlinearity}$=0.43

Supplementary Figure 20. Peanuts and stroke, high vs. low analysis

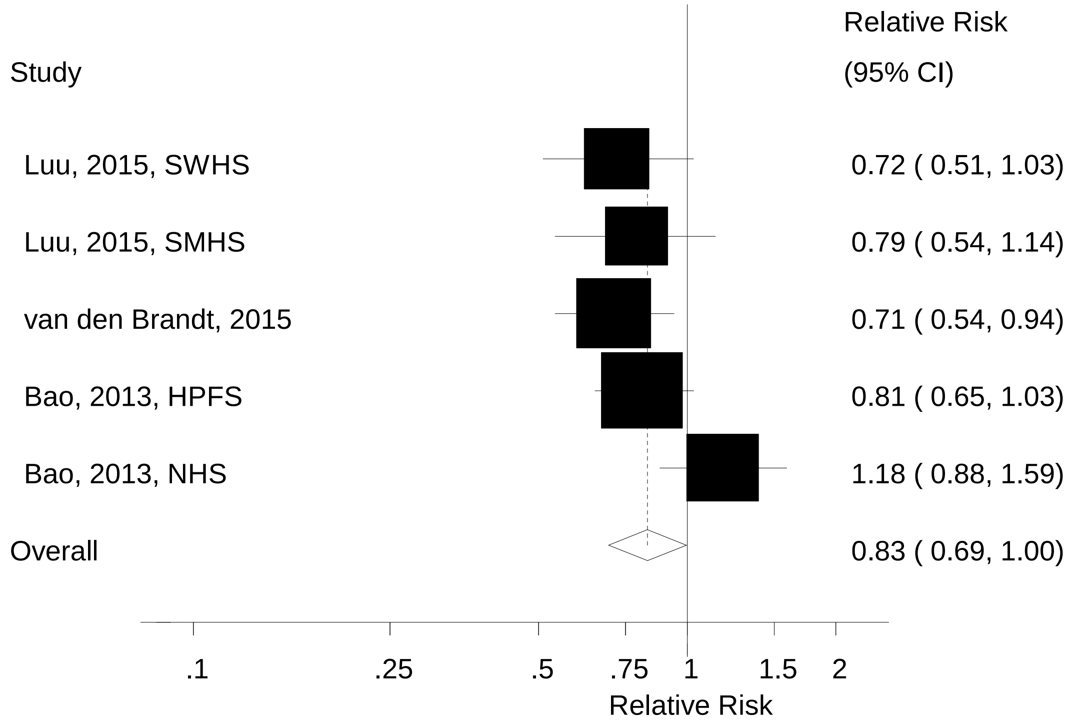

Supplementary Figure 21. Peanuts and stroke, dose-response analysis

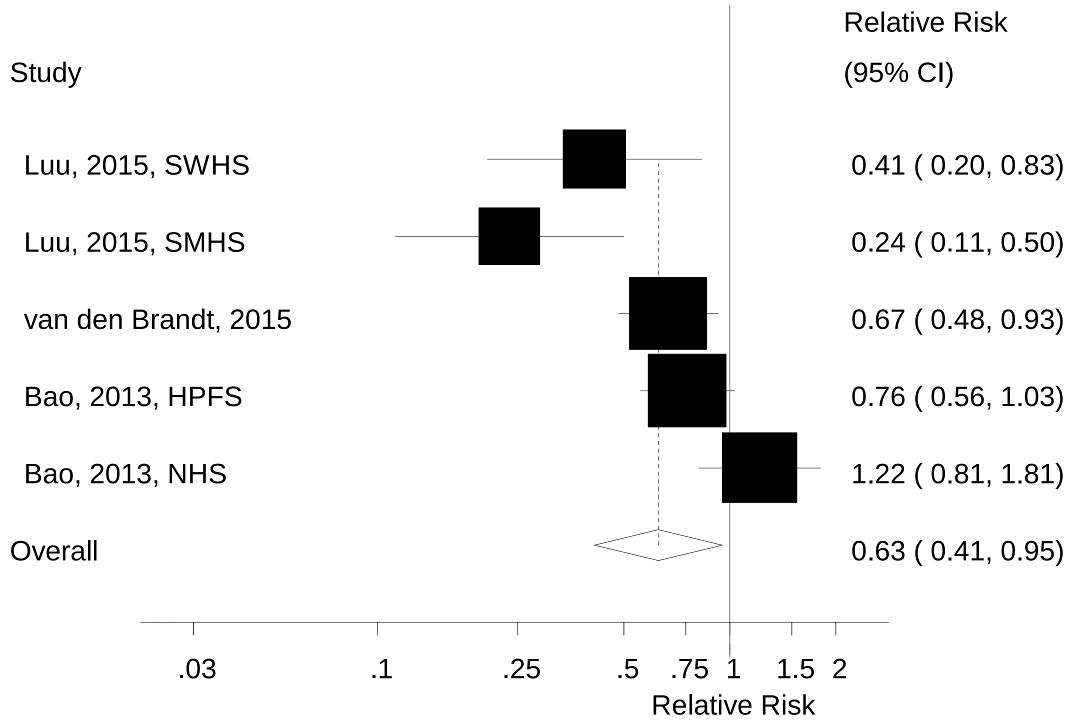

Supplementary Figure 22. Peanuts and stroke, nonlinear dose-response analysis

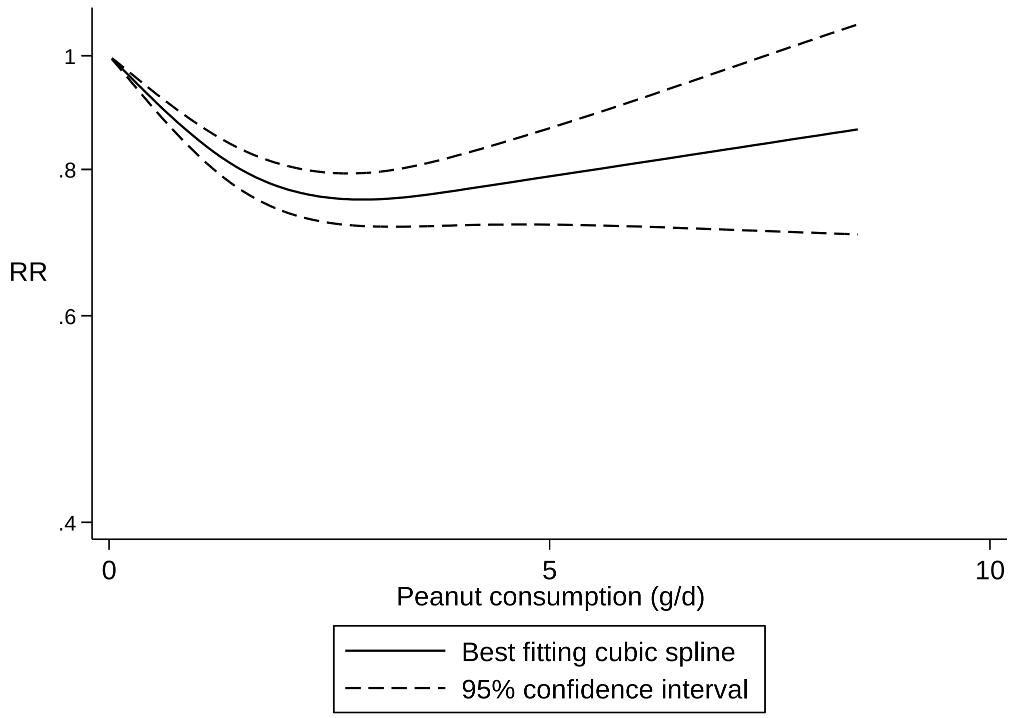

$p_{nonlinearity}$<0.0001

Supplementary Figure 23. Tree nuts and cardiovascular disease, high vs. low analysis

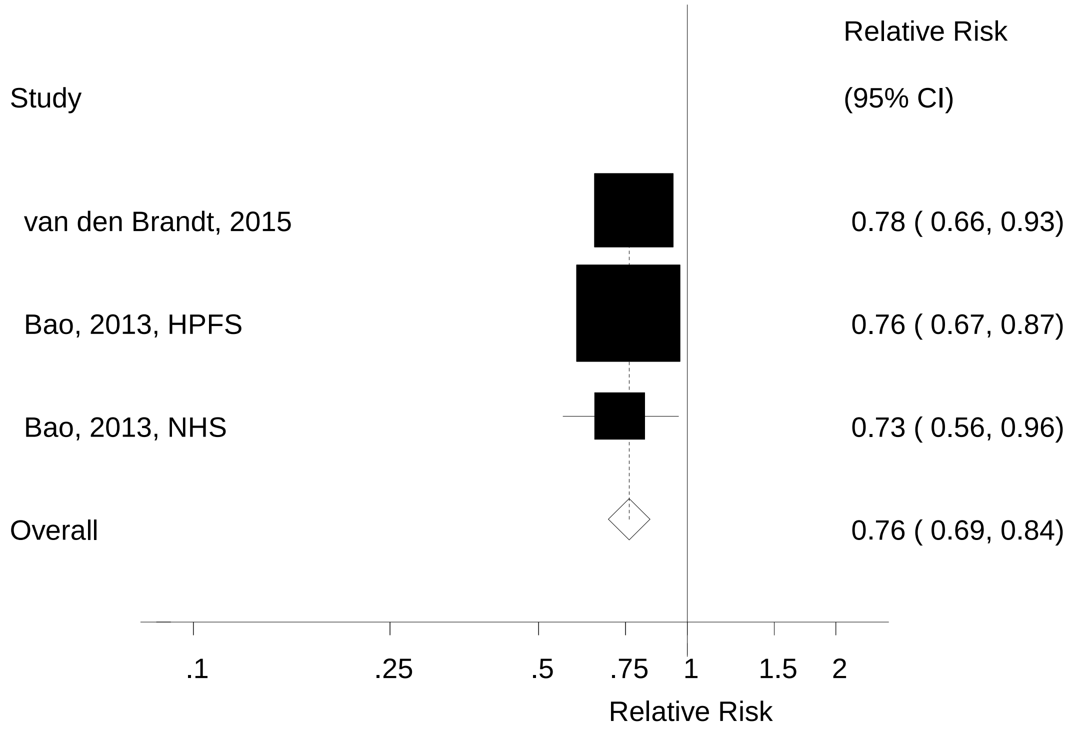

Supplementary Figure 24. Tree nuts and cardiovascular disease, dose-response analysis, per 10 g/d

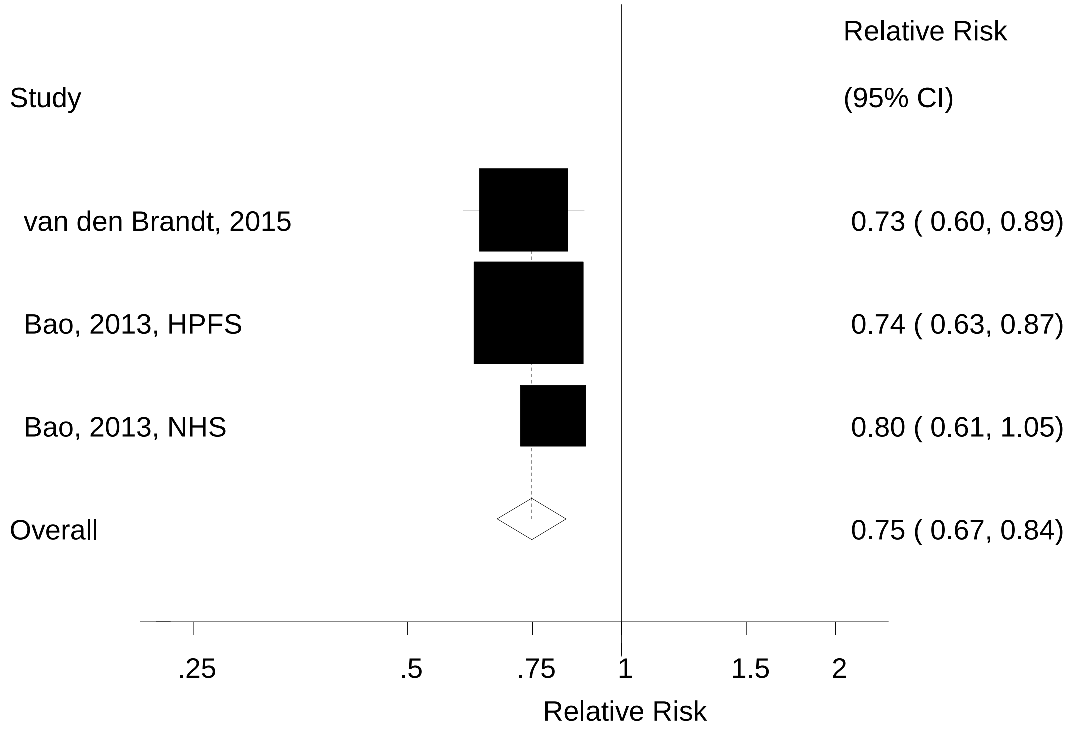

Supplementary Figure 25. Tree nuts and cardiovascular disease, nonlinear dose-response analysis

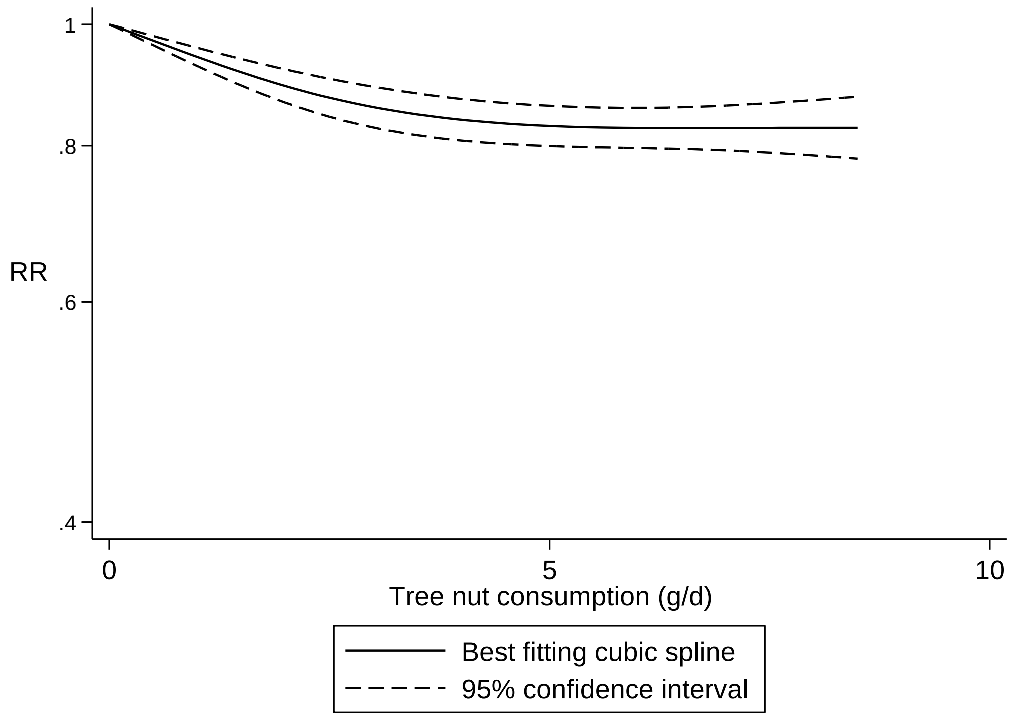

$p_{nonlinearity}$<0.0001

Supplementary Figure 26. Peanuts and cardiovascular disease, high vs. low analysis

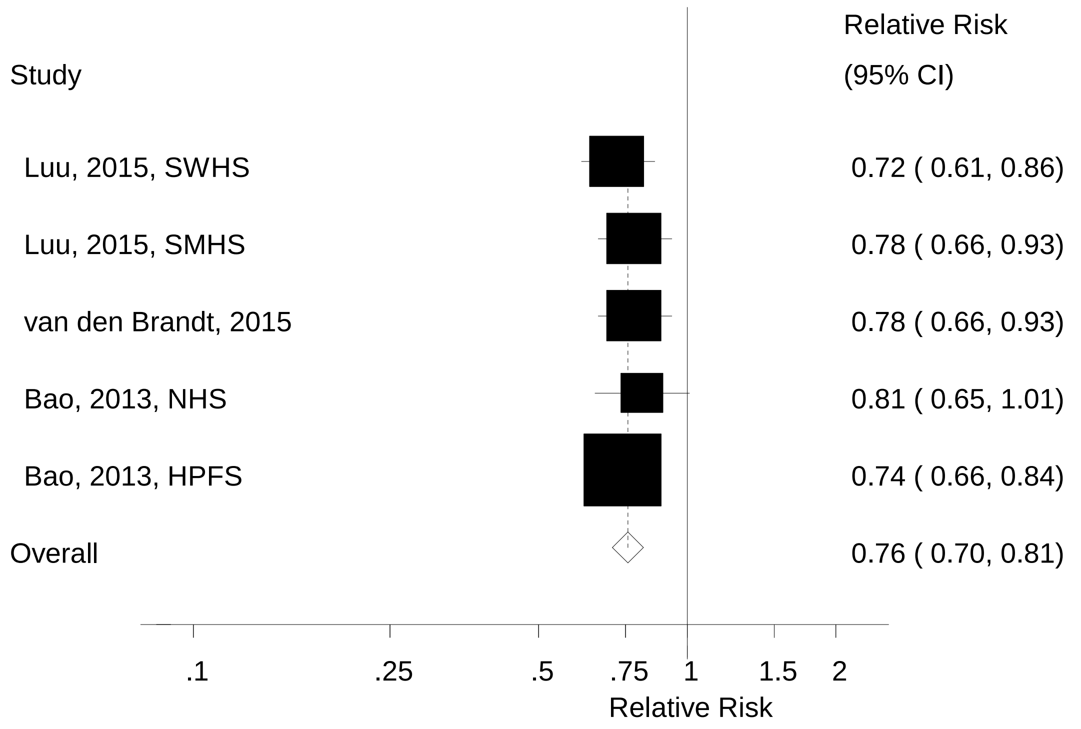

Supplementary Figure 27. Peanuts and cardiovascular disease, dose-response analysis

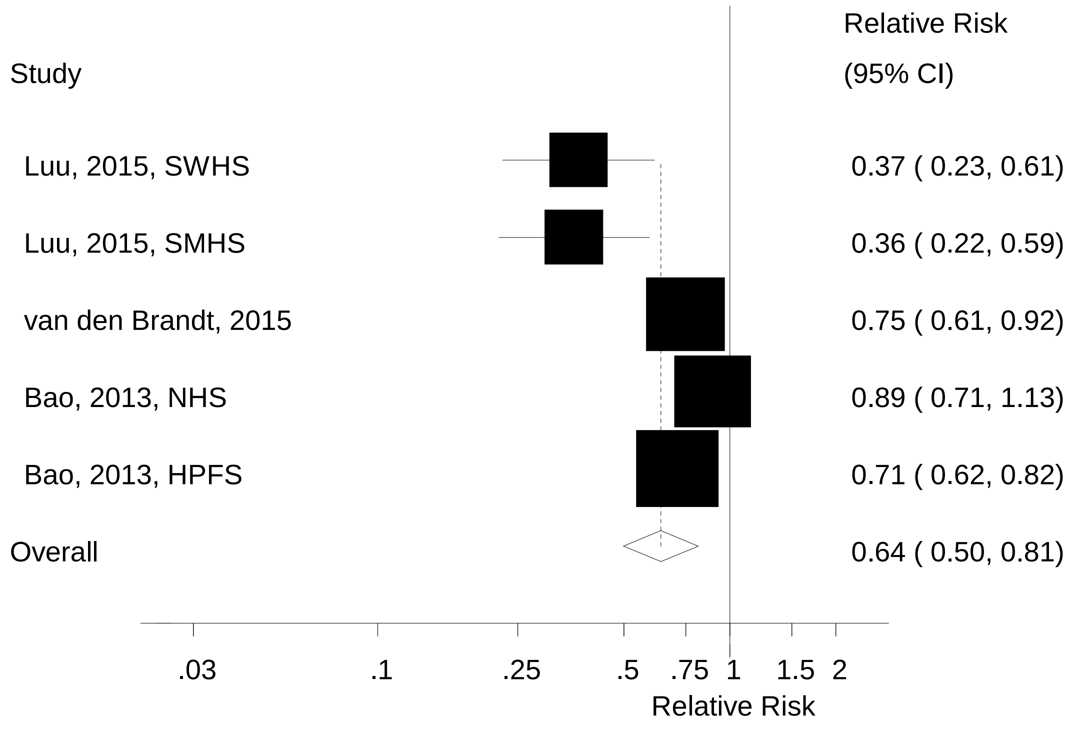

Supplementary Figure 28. Peanuts and cardiovascular disease, nonlinear dose-response analysis

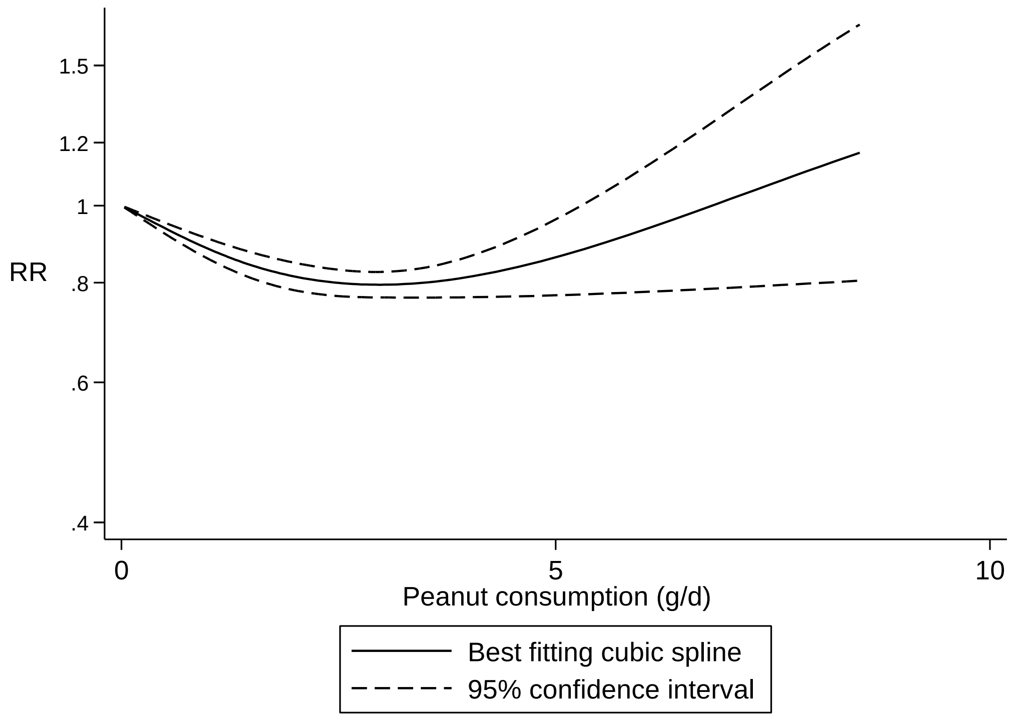

$p_{nonlinearity}$<0.0001

Supplementary Figure 29. Tree nuts and total cancer, high vs. low analysis

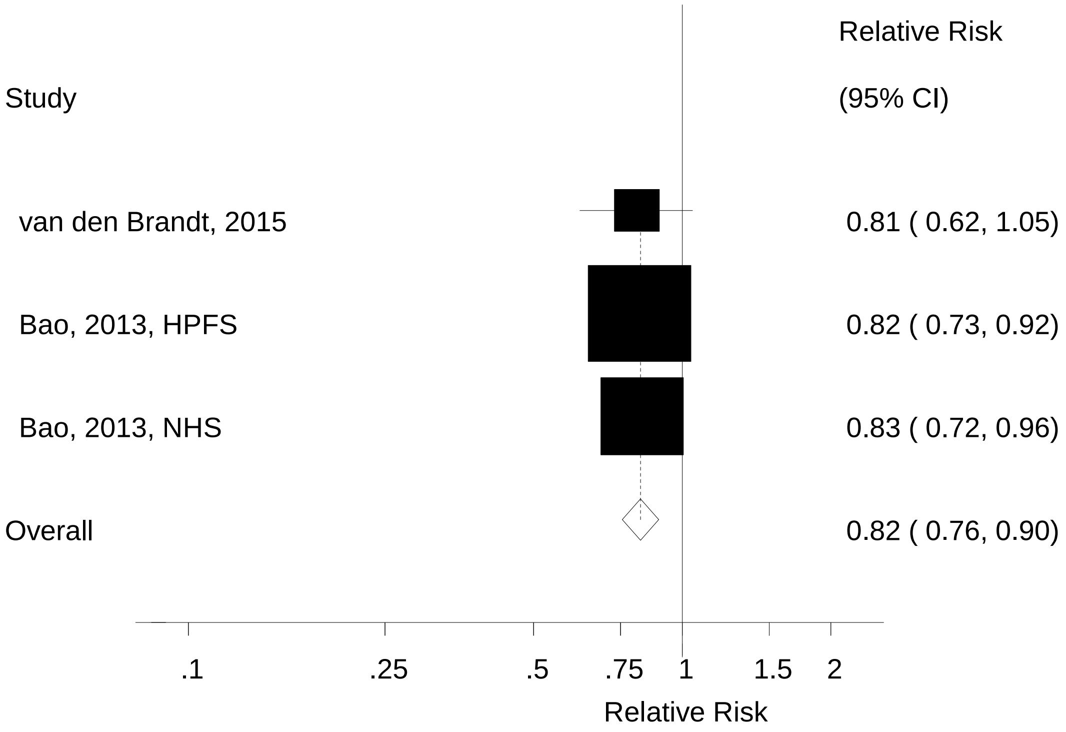

Supplementary Figure 30. Tree nuts and total cancer, dose-response analysis

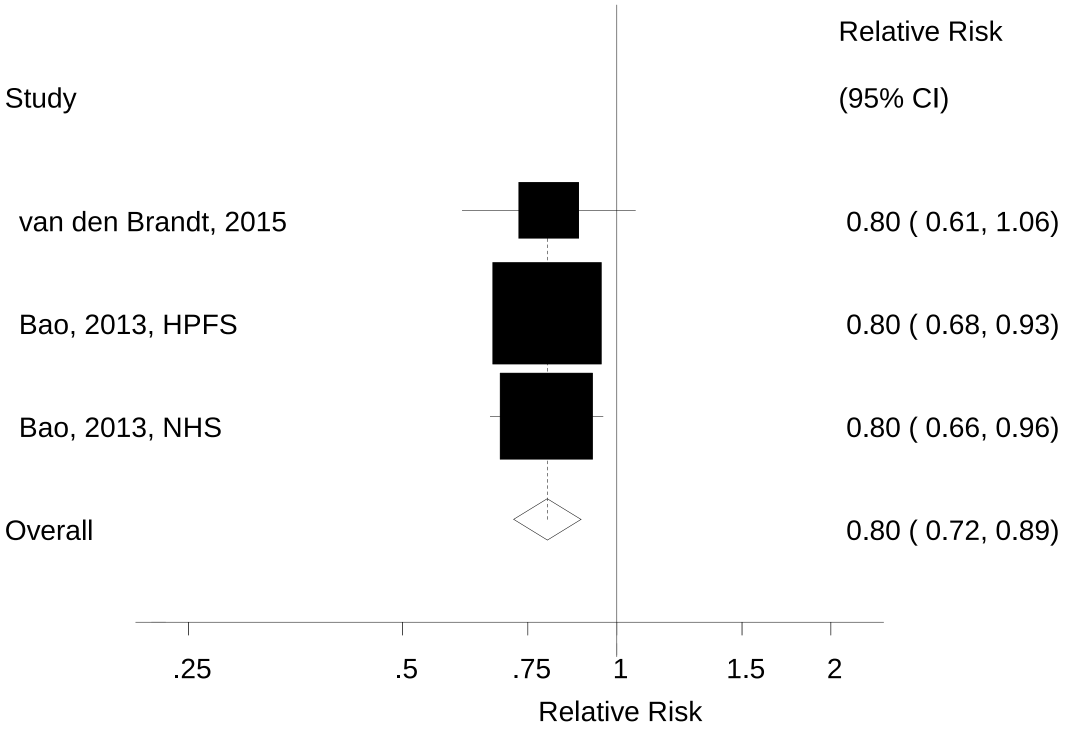

Supplementary Figure 31. Tree nuts and total cancer, nonlinear dose-response analysis

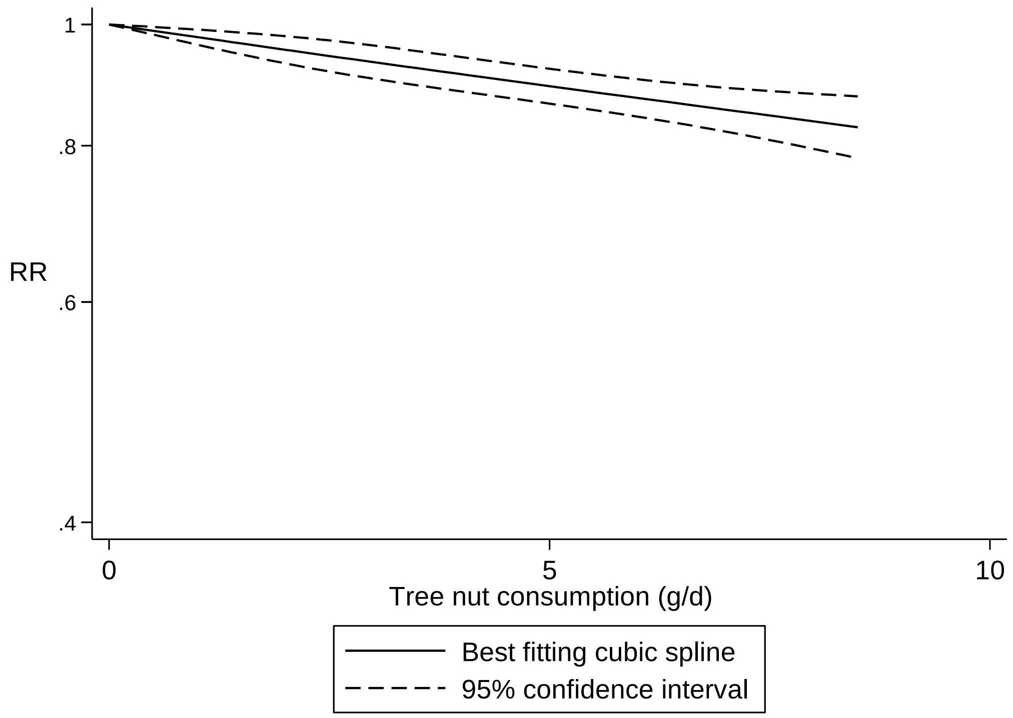

$p_{nonlinearity}$=0.89

Supplementary Figure 32. Peanuts and total cancer, high vs. low analysis

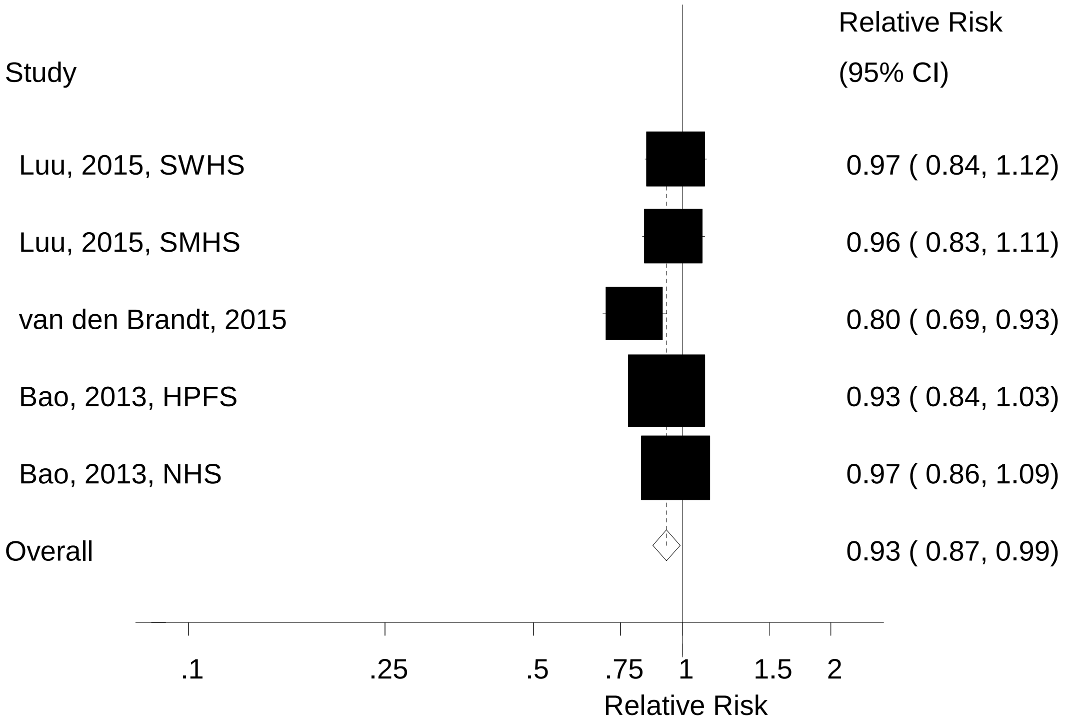

Supplementary Figure 33. Peanuts and total cancer, dose-response analysis

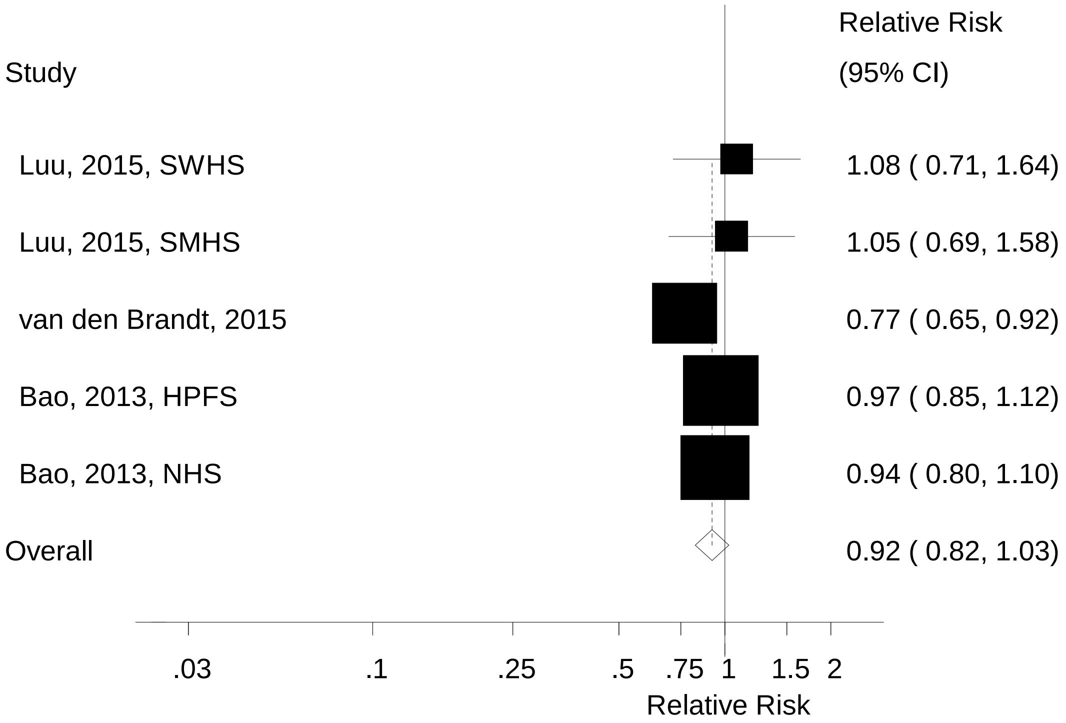

Supplementary Figure 34. Peanuts and total cancer, nonlinear dose-response analysis

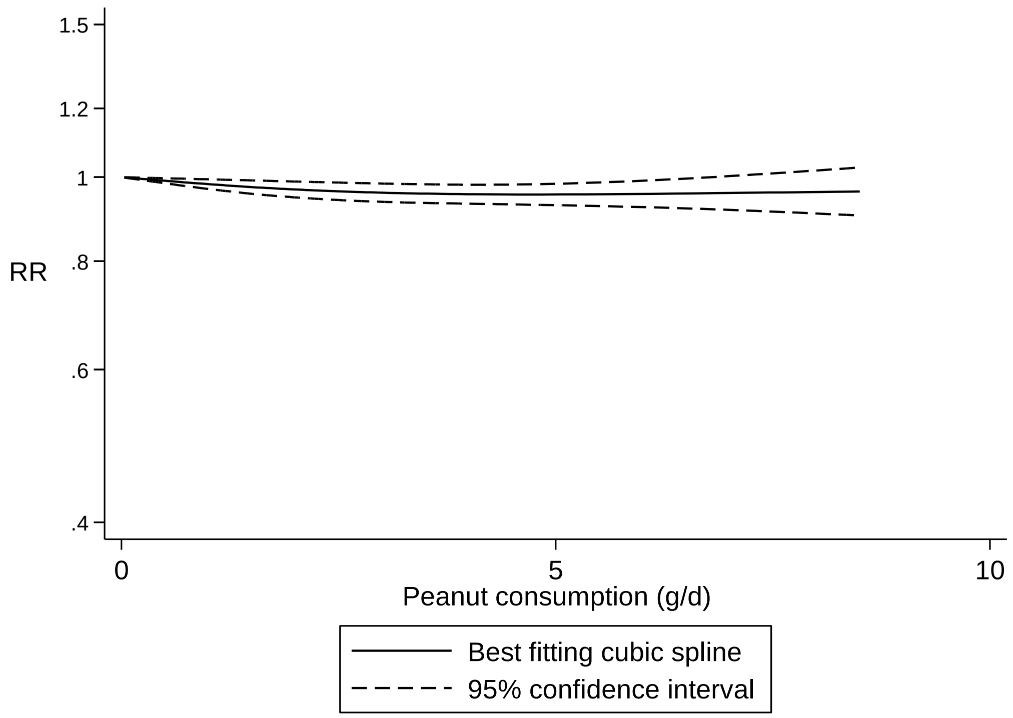

$p_{nonlinearity}$=0.10

Supplementary Figure 35. Funnel plot of nuts and all-cause mortality

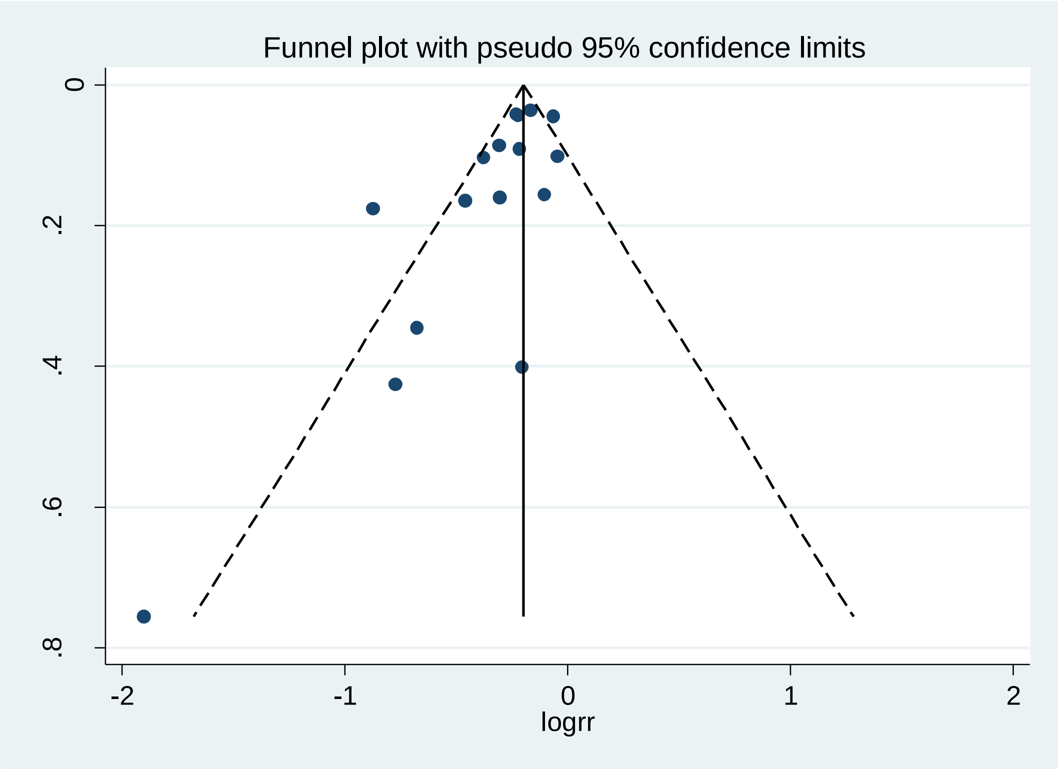

Supplementary Figure 36. Tree nuts and all-cause mortality, high vs. low analysis

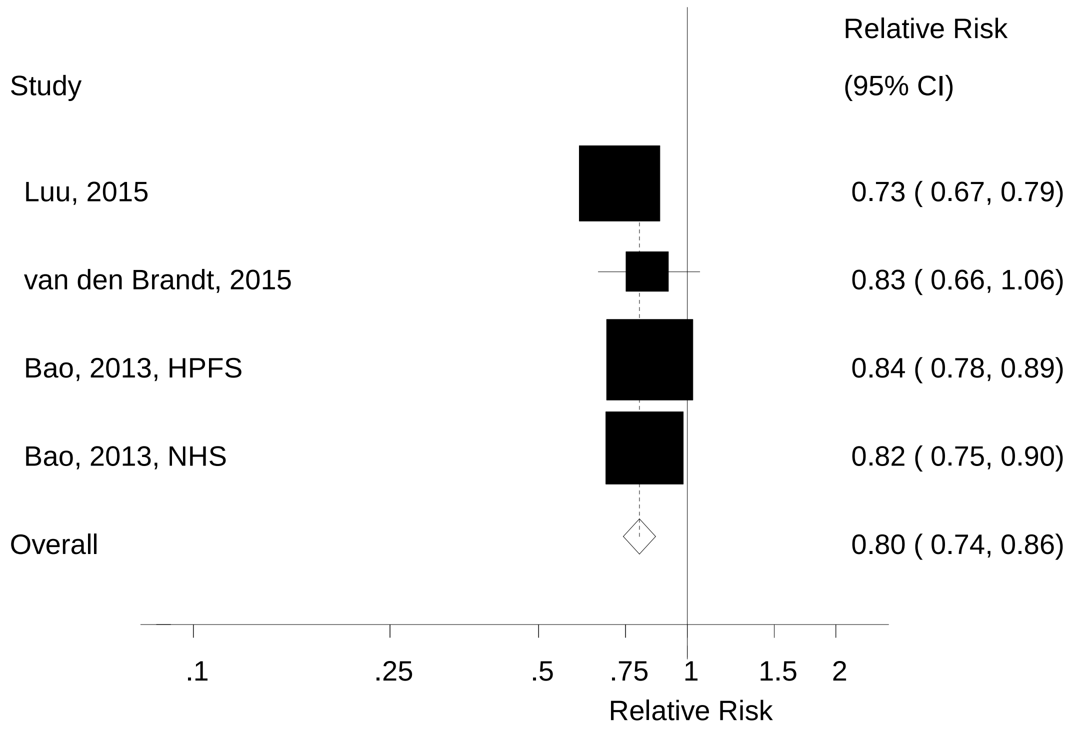

Supplementary Figure 37. Tree nuts and all-cause mortality, dose-response analysis

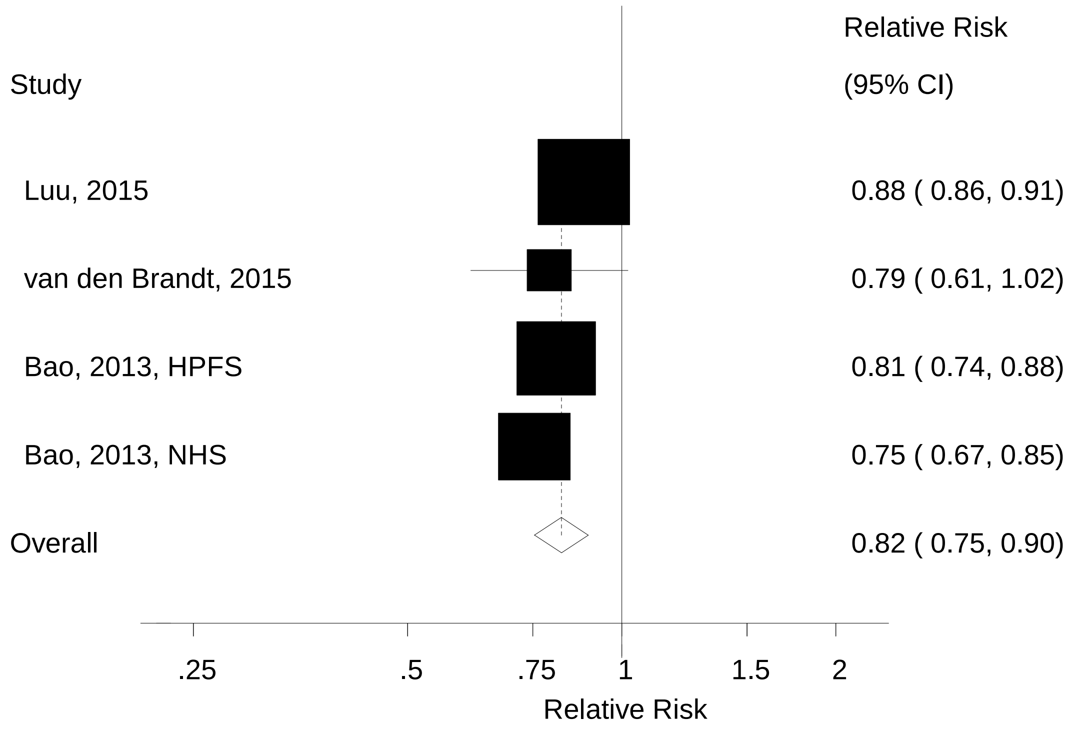

Supplementary Figure 38. Tree nuts and all-cause mortality, nonlinear dose-response analysis

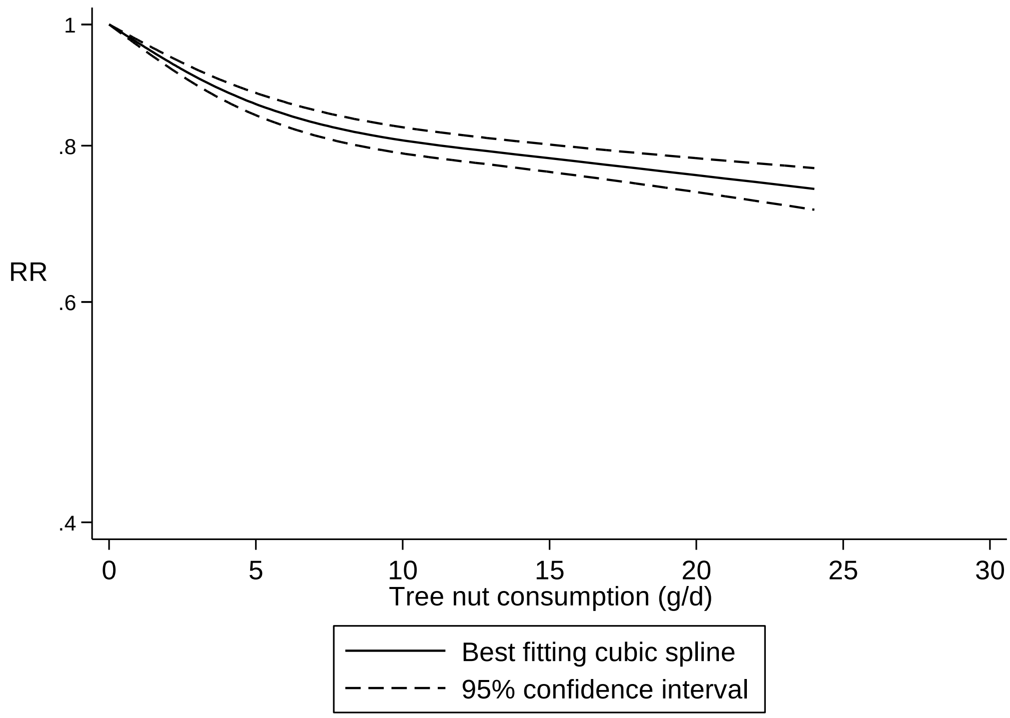

$p_{nonlinearity}$<0.0001

Supplementary Figure 39. Peanuts and all-cause mortality, high vs. low analysis

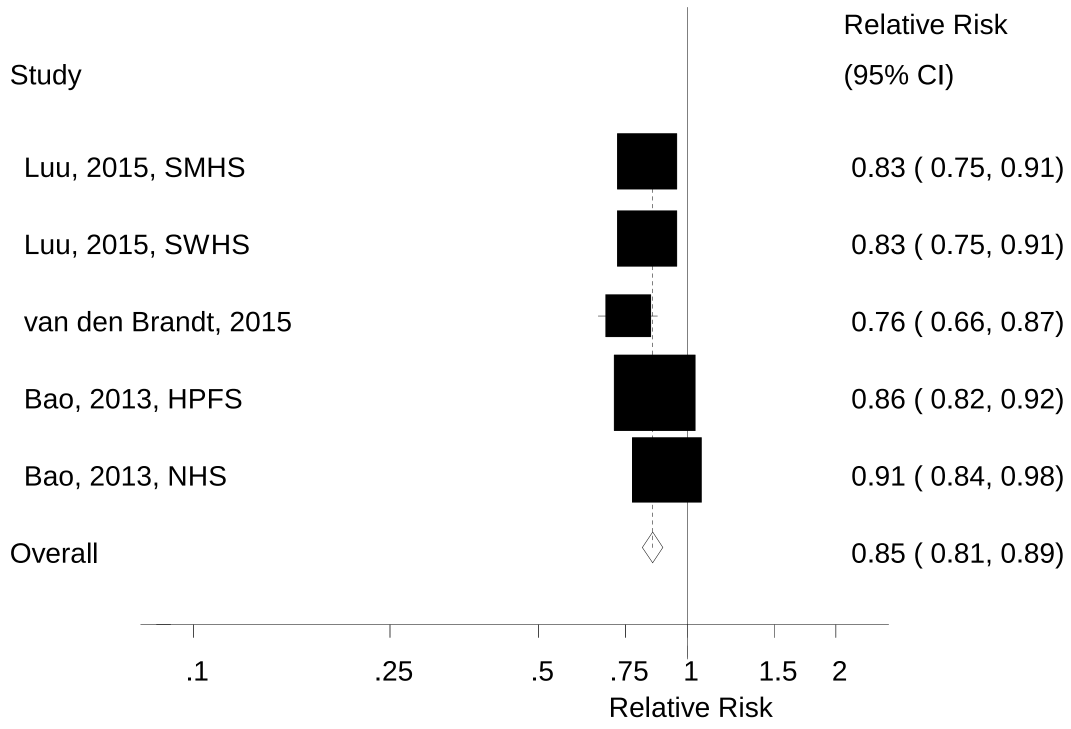

Supplementary Figure 40. Peanuts and all-cause mortality, dose-response analysis

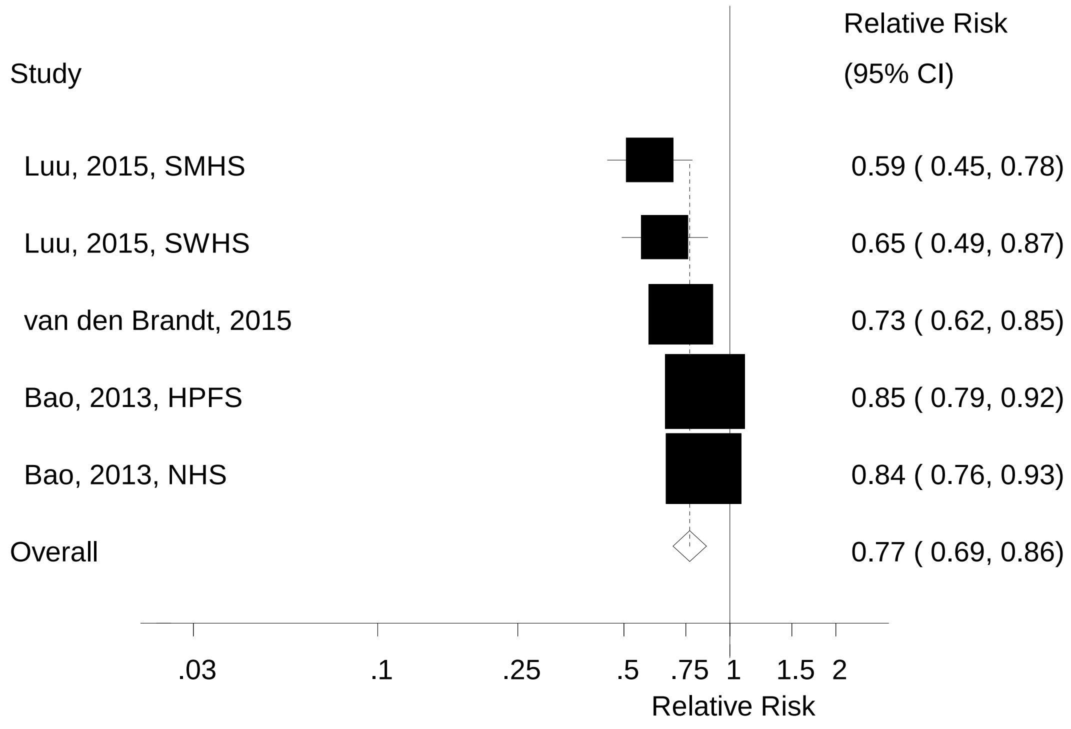

Supplementary Figure 41. Peanuts and all-cause mortality, nonlinear dose-response analysis

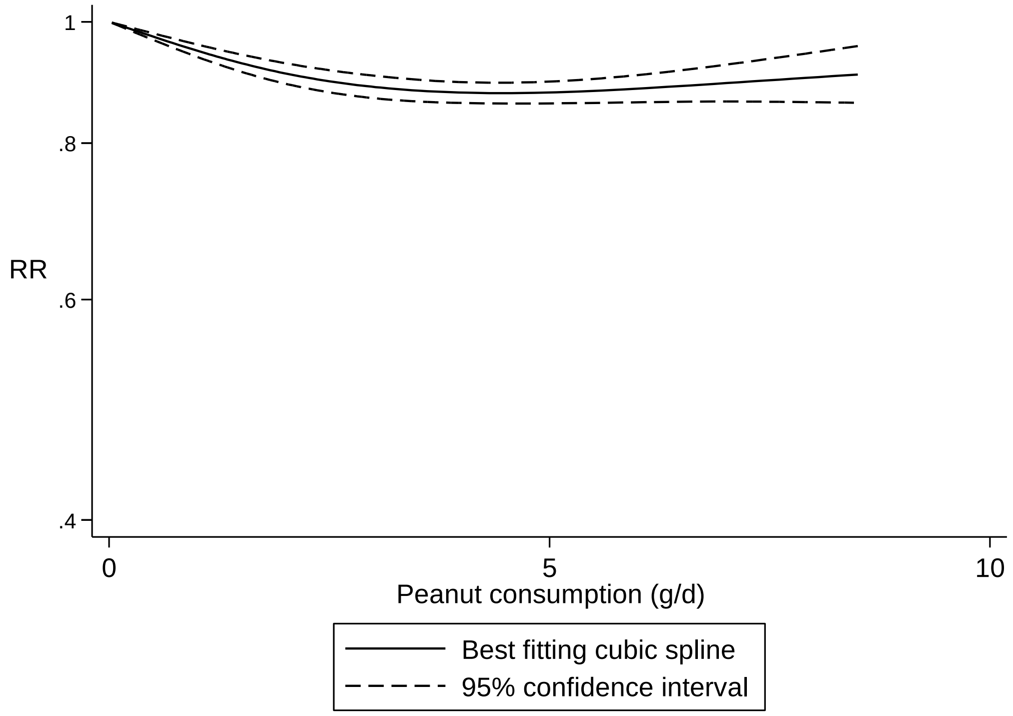

$p_{nonlinearity}$<0.0001

Supplementary Figure 42. Peanut butter and all-cause mortality, high vs. low analysis

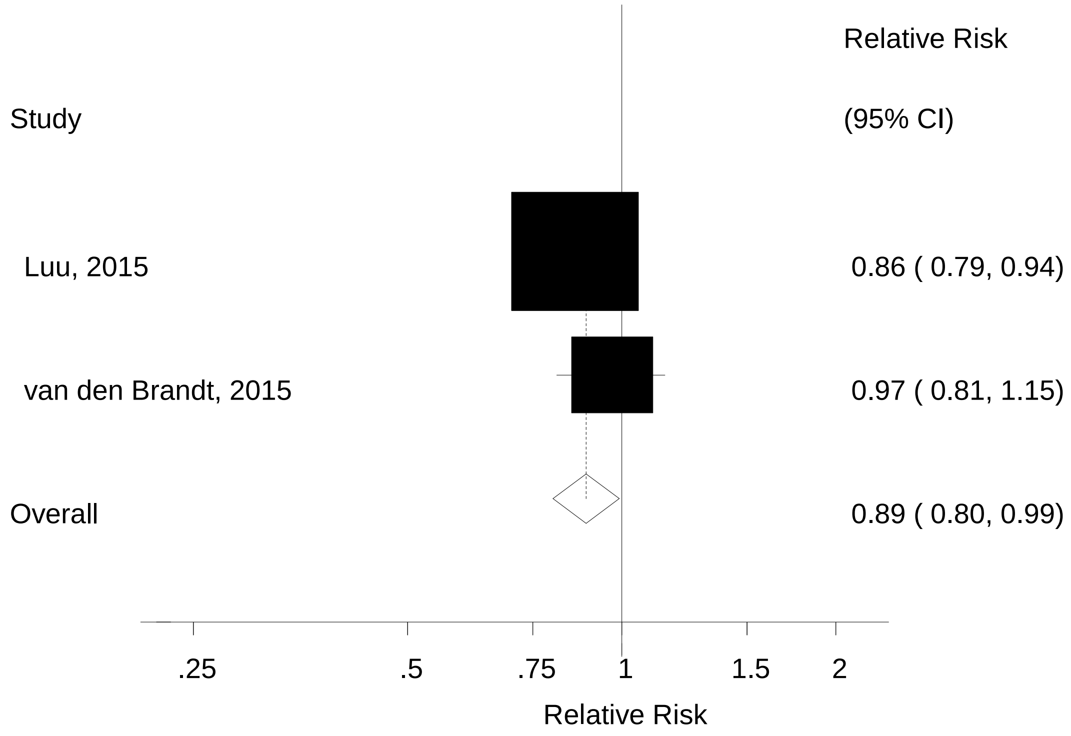

Supplementary Figure 43. Peanut butter and all-cause mortality, dose-response analysis

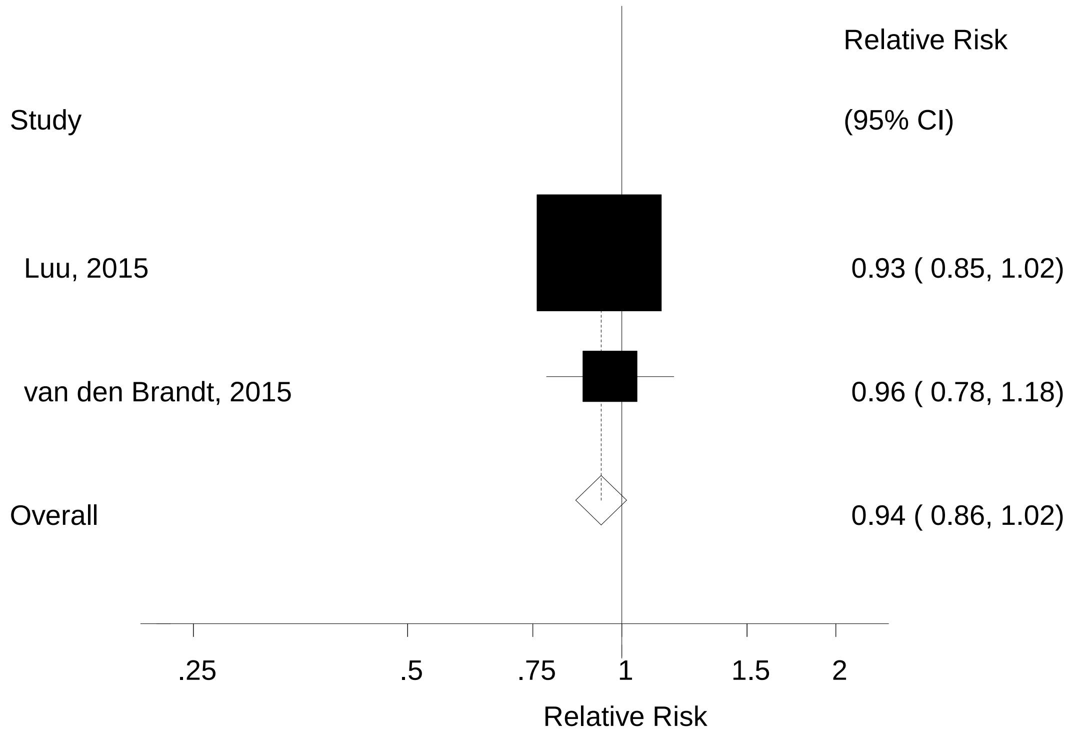

Supplementary Figure 44. Tree nuts and respiratory disease mortality, high vs. low analysis

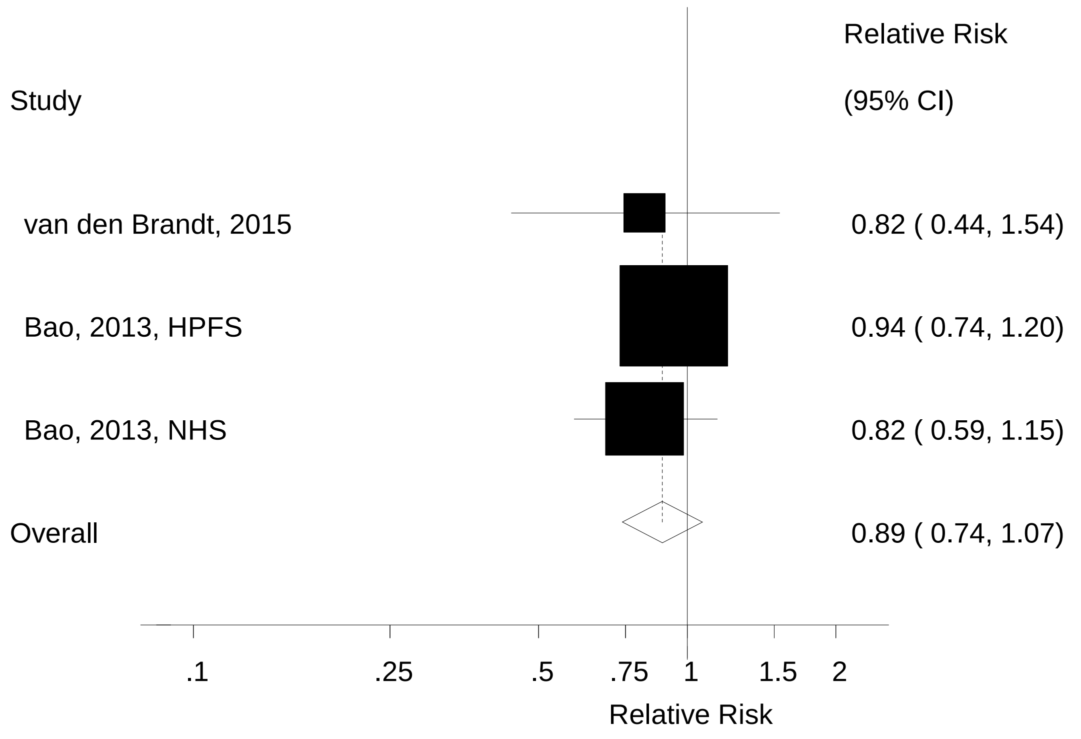

Supplementary Figure 45. Tree nuts and respiratory disease mortality, dose-response analysis

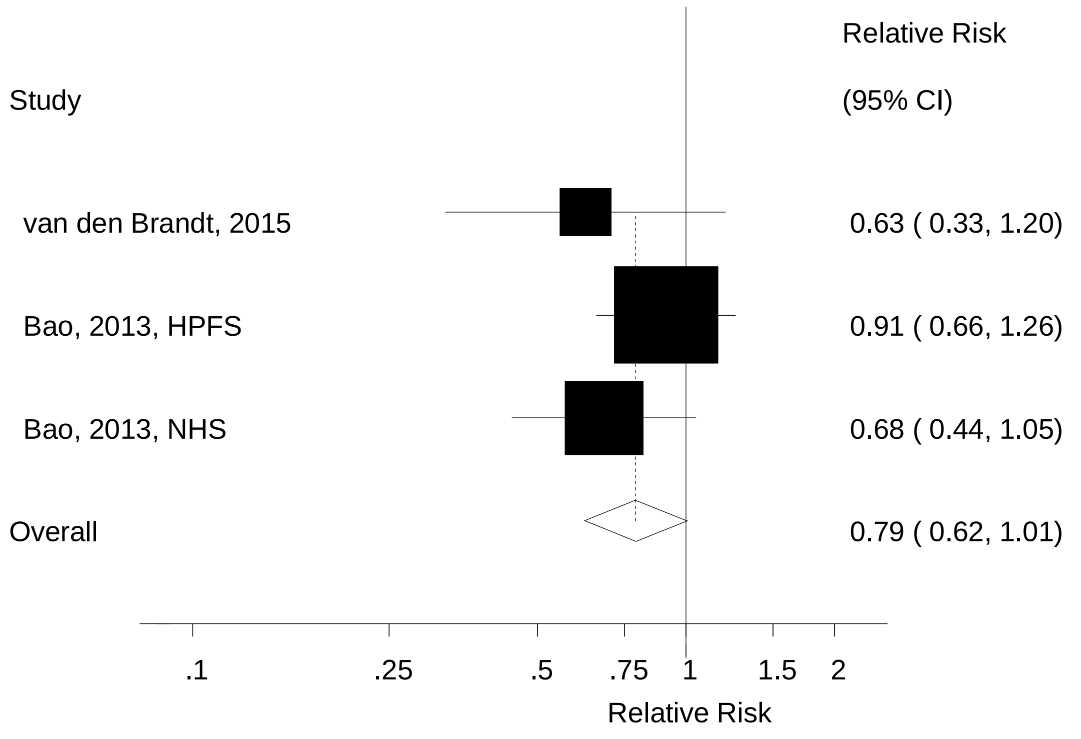

Supplementary Figure 46. Tree nuts and respiratory disease mortality, nonlinear dose-response analysis

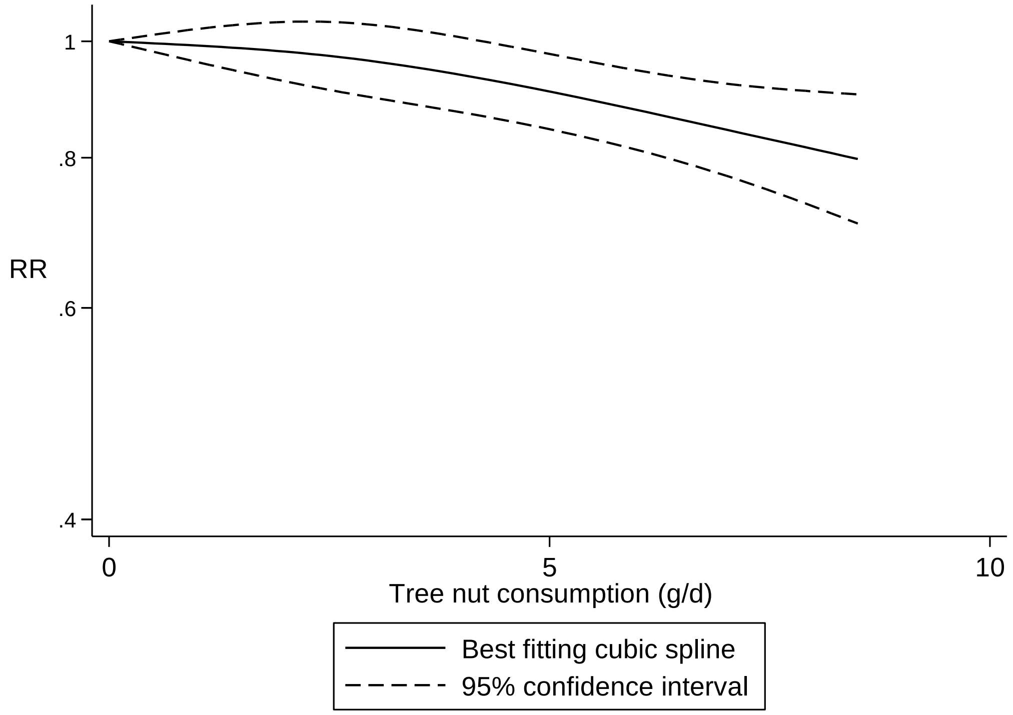

$p_{nonlinearity}$=0.33

Supplementary Figure 47. Peanuts and respiratory disease mortality, high vs. low analysis

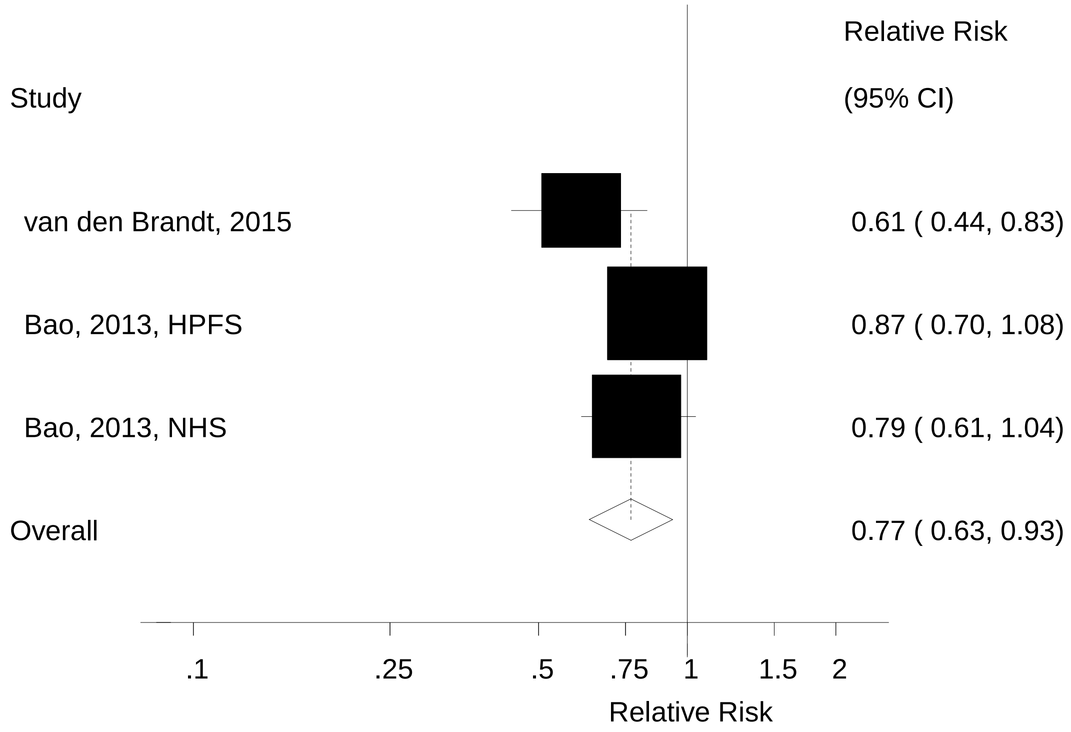

Supplementary Figure 48. Peanuts and respiratory disease mortality, dose-response analysis

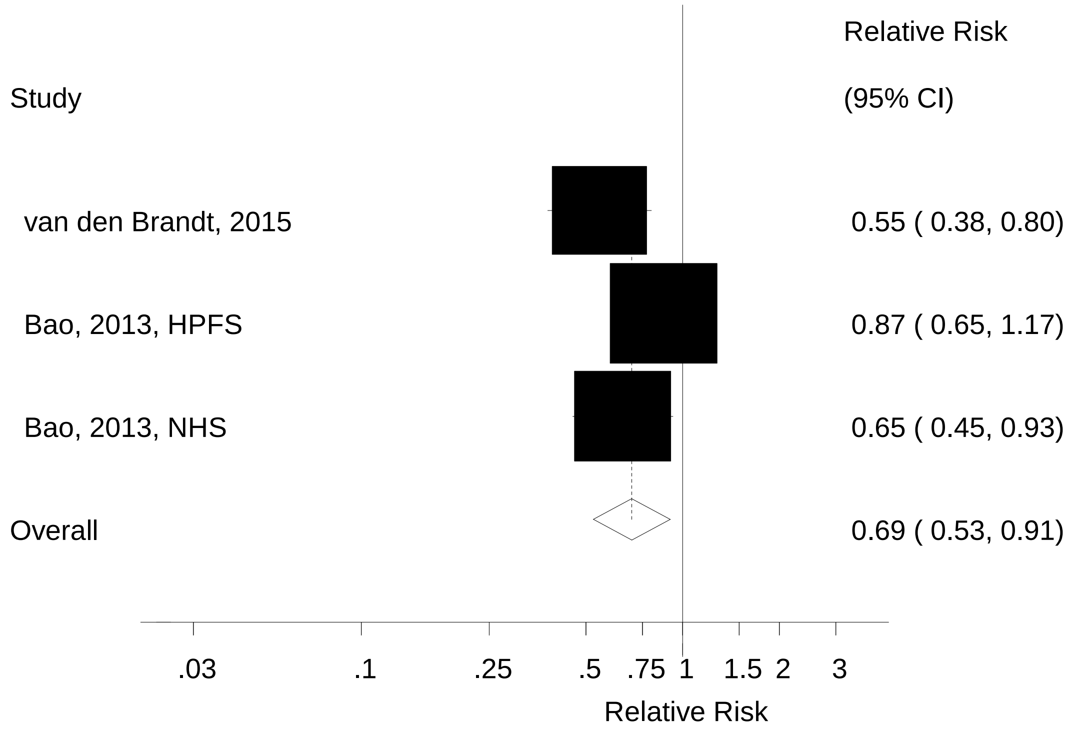

Supplementary Figure 49. Peanuts and respiratory disease mortality, nonlinear dose-response analysis

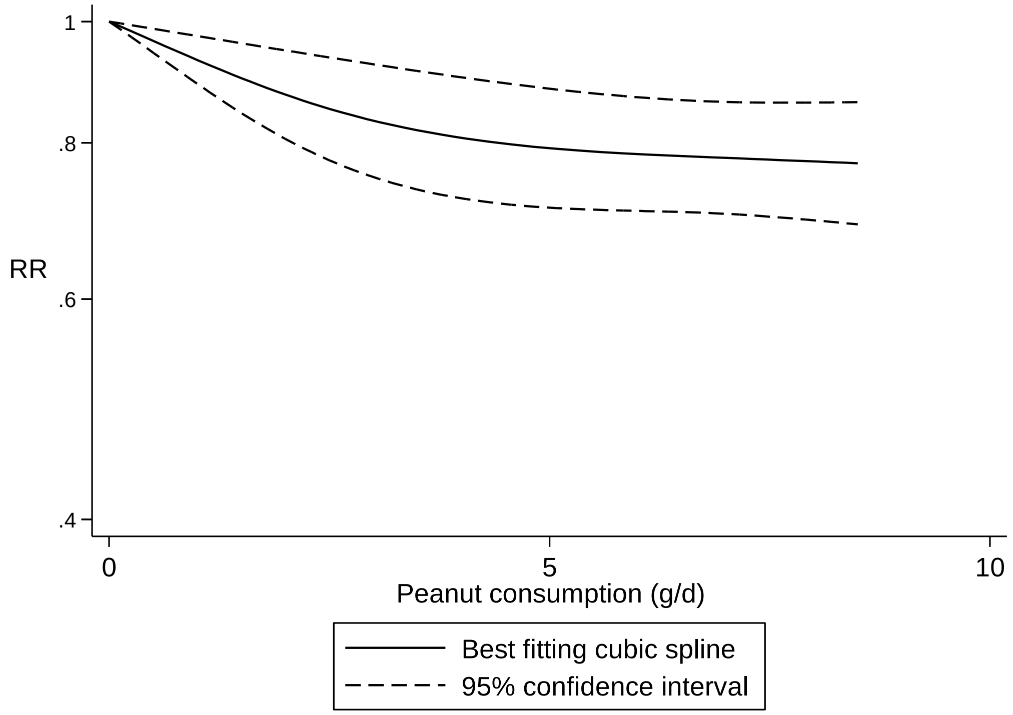

$p_{nonlinearity}$=0.06

Supplementary Figure 50. Tree nuts and diabetes mortality, high vs. low analysis

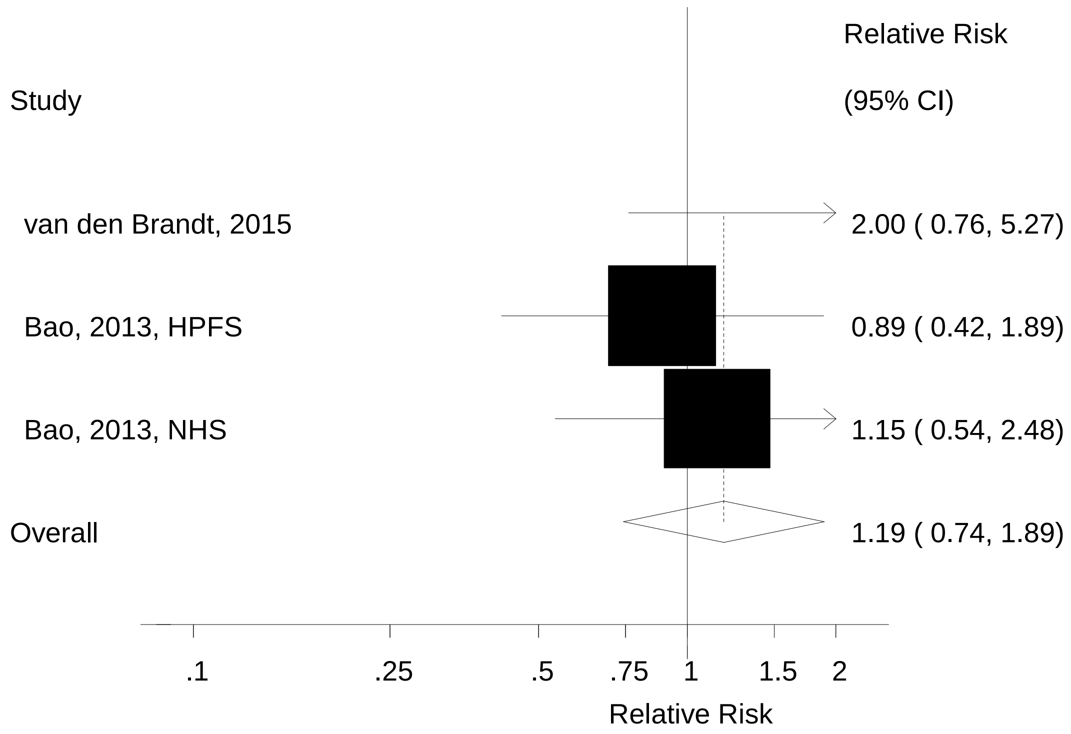

Supplementary Figure 51. Tree nuts and diabetes mortality, dose-response analysis

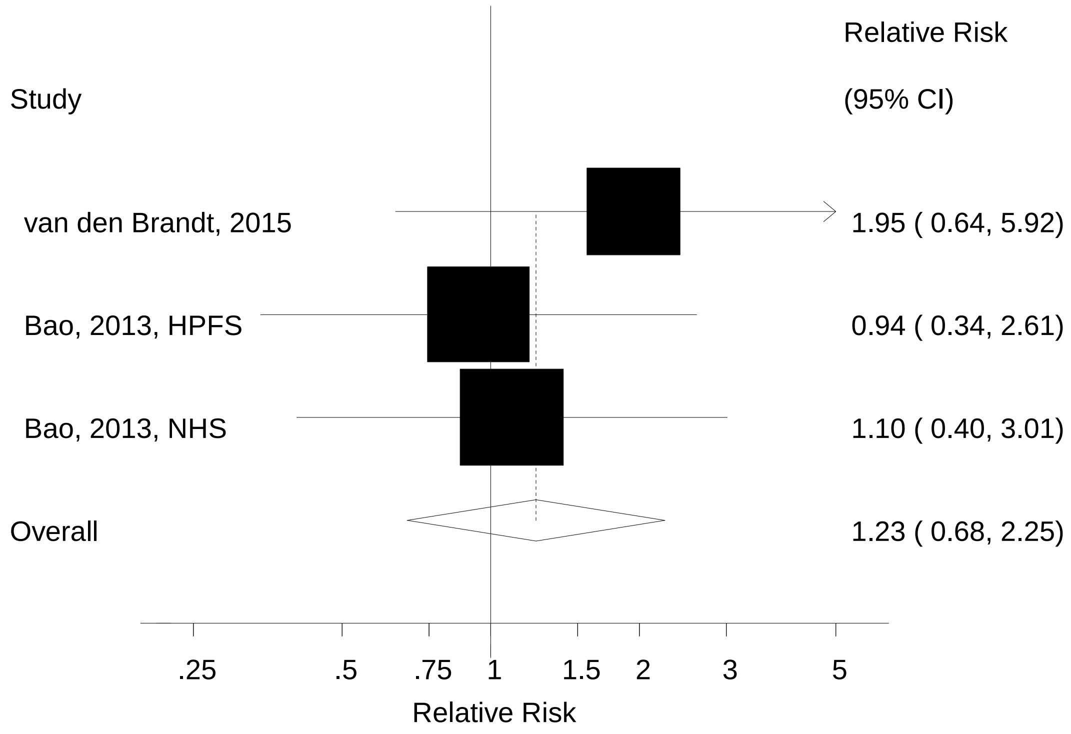

Supplementary Figure 52. Tree nuts and diabetes mortality, nonlinear dose-response analysis

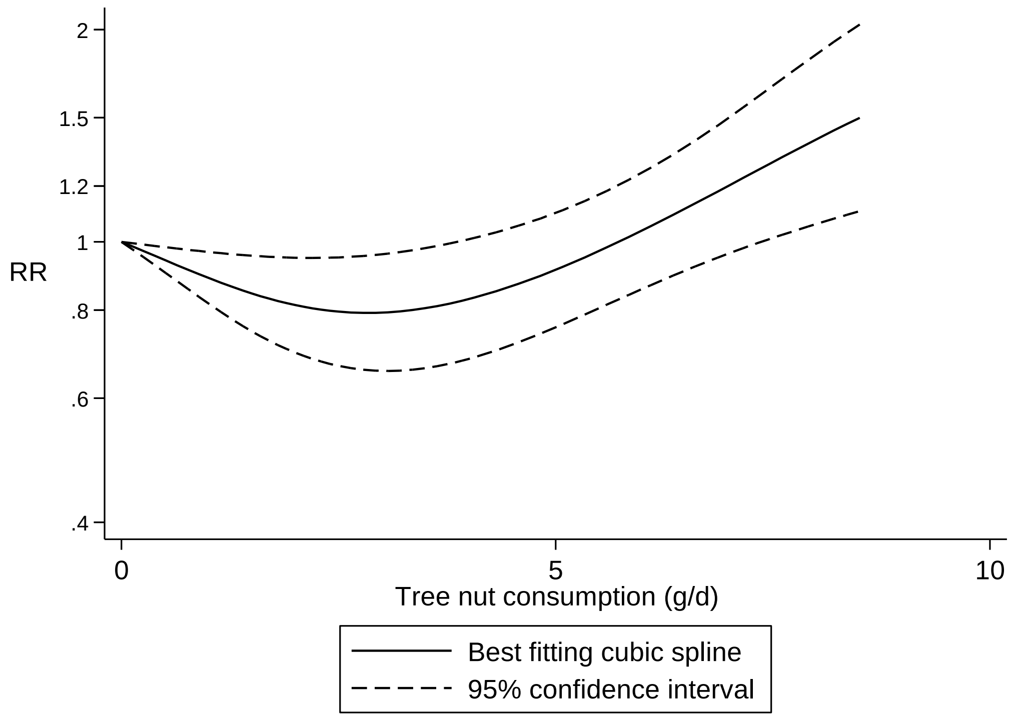

$p_{nonlinearity}$=0.001

Supplementary Figure 53. Peanuts and diabetes mortality, high vs. low analysis

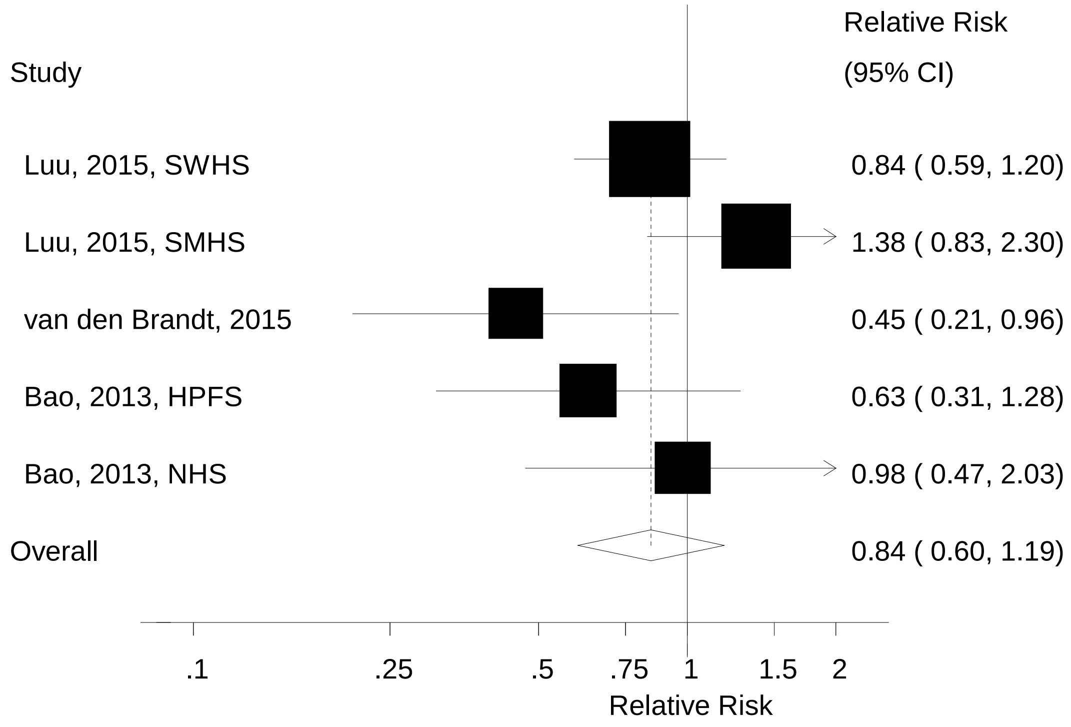

Supplementary Figure 54. Peanuts and diabetes mortality, dose-response analysis

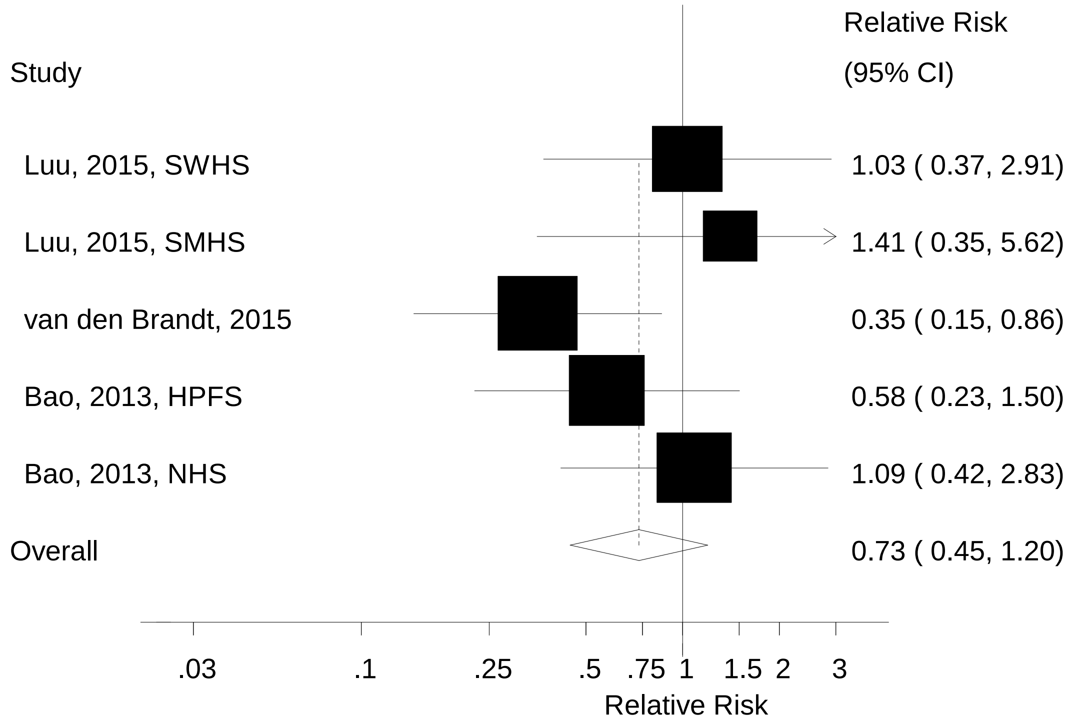

Supplementary Figure 55. Peanuts and diabetes mortality, nonlinear dose-response analysis

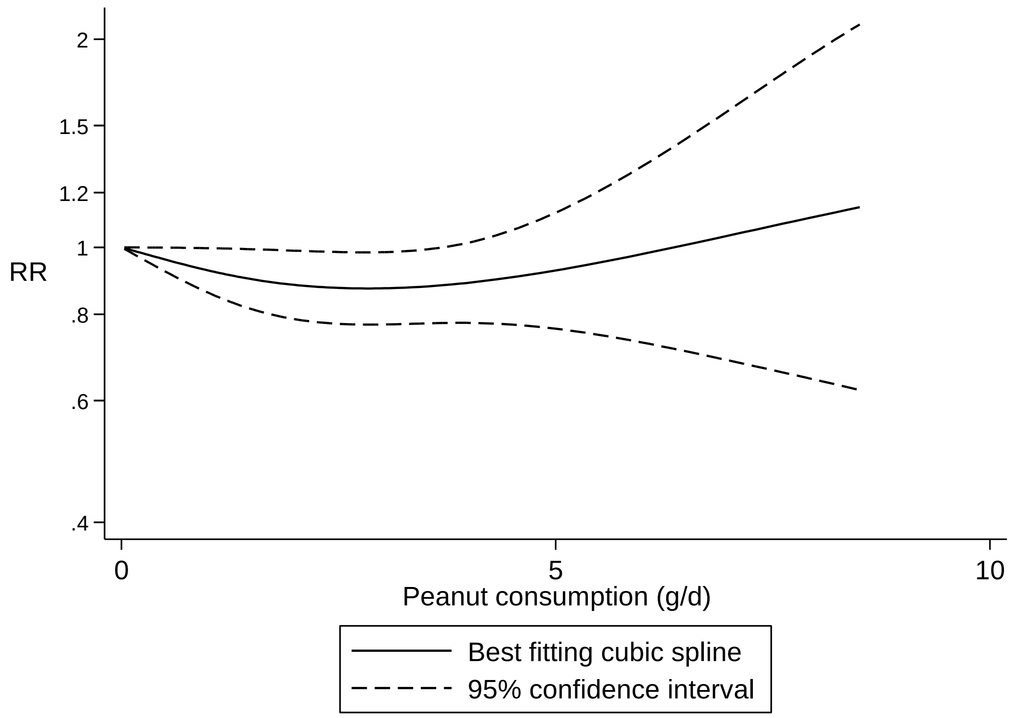

$p_{nonlinearity}<0.0001$

Supplementary Figure 56. Tree nuts and neurodegenerative disease mortality, high vs. low analysis

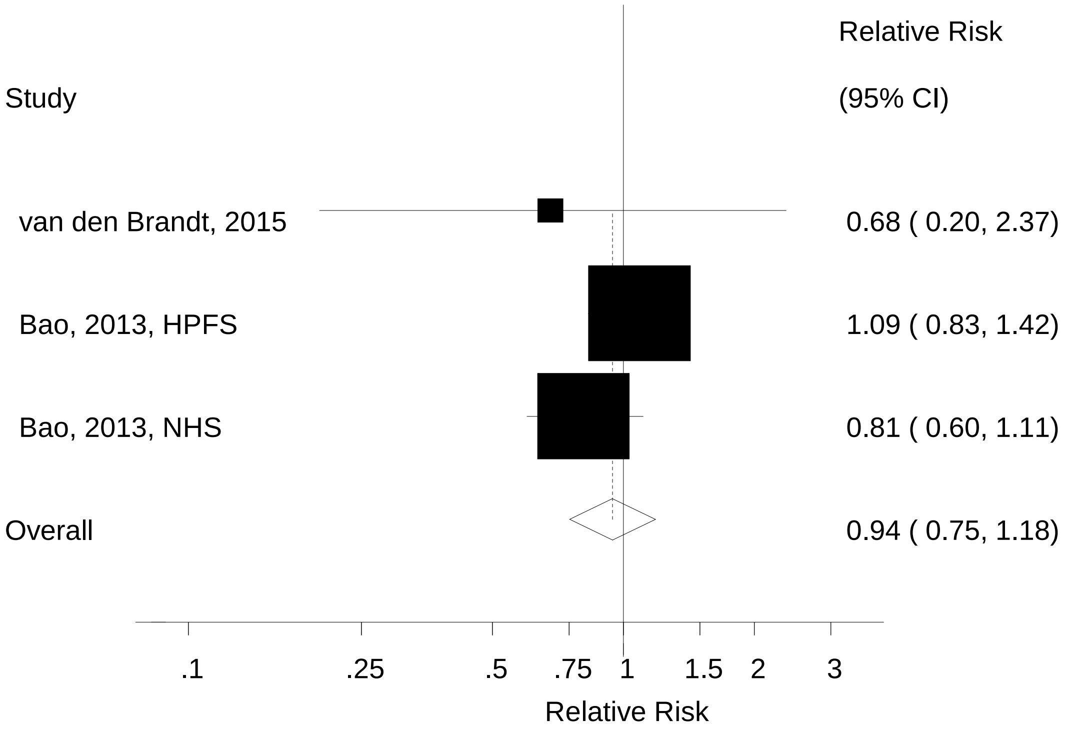

Supplementary Figure 57. Tree nuts and neurodegenerative disease mortality, dose-response analysis

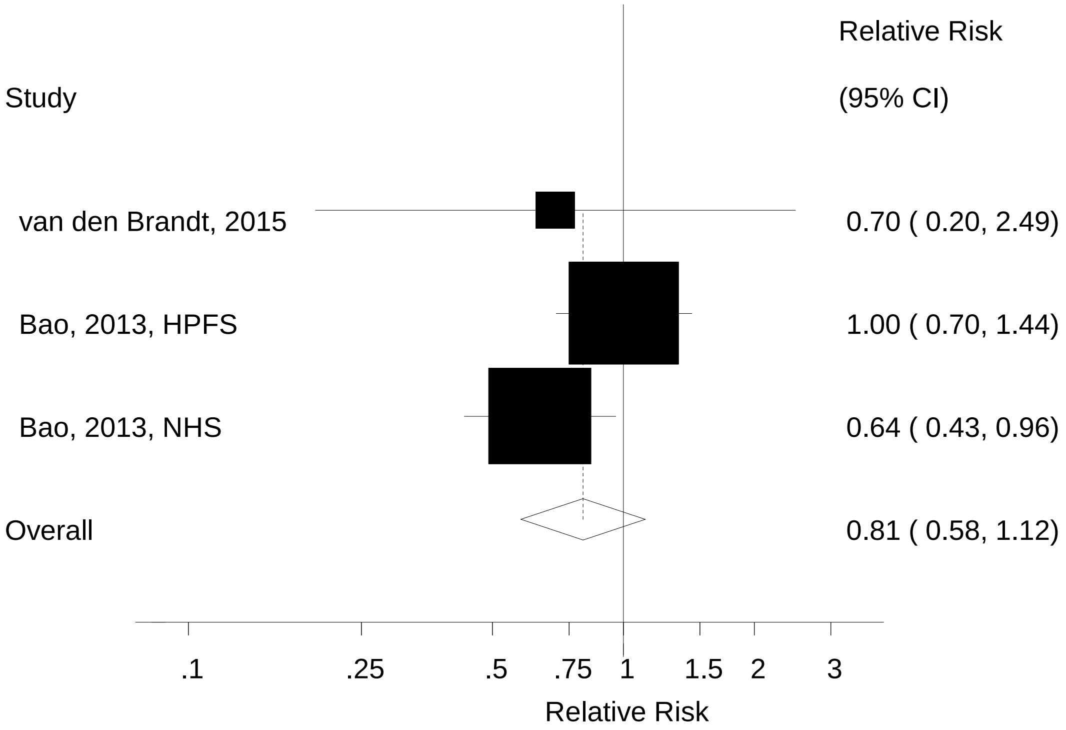

Supplementary Figure 58. Tree nuts and neurodegenerative disease mortality, nonlinear dose-response analysis

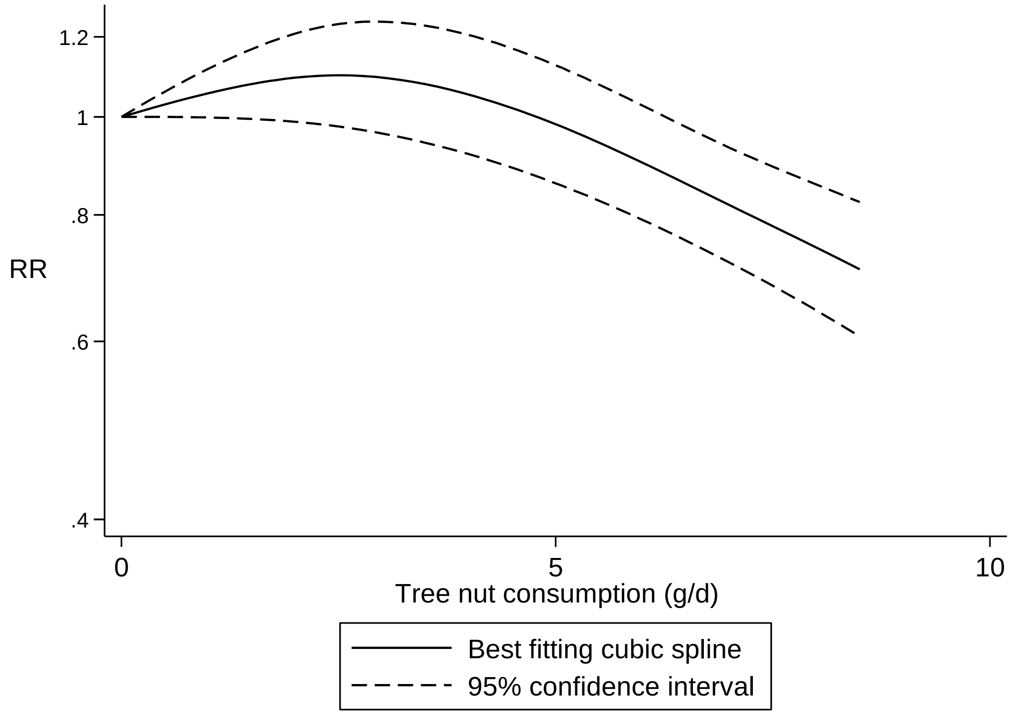

$p_{nonlinearity}$=0.16

Supplementary Figure 59. Peanuts and neurodegenerative disease mortality, high vs. low analysis

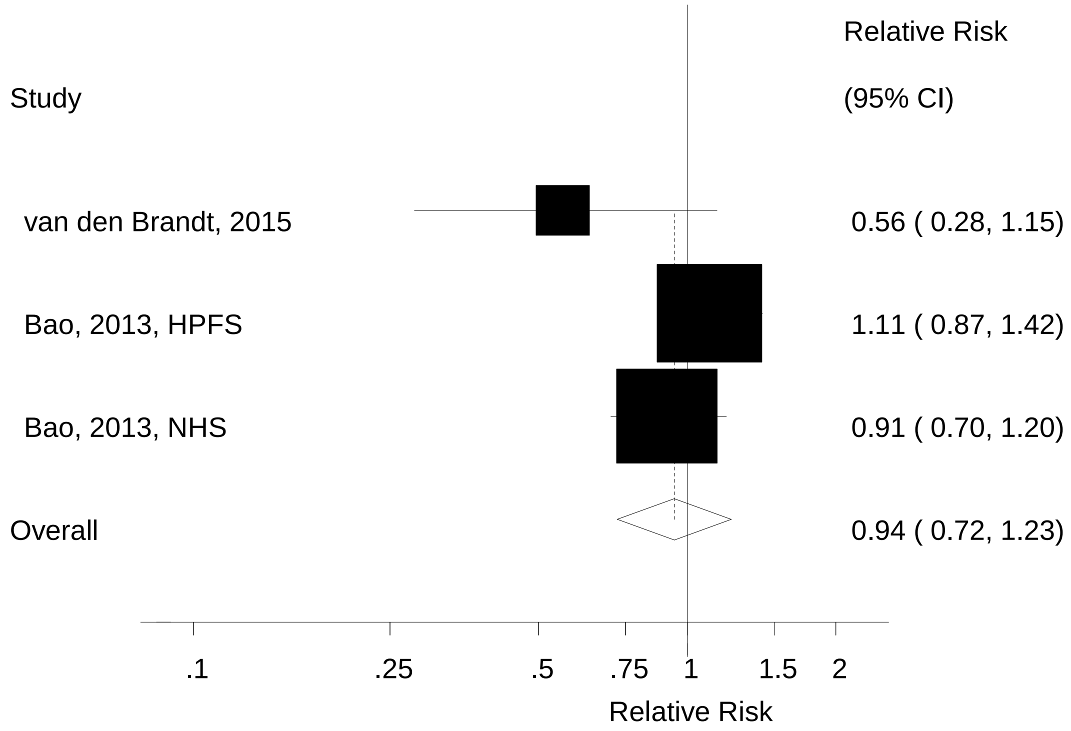

Supplementary Figure 60. Peanuts and neurodegenerative disease mortality, dose-response analysis

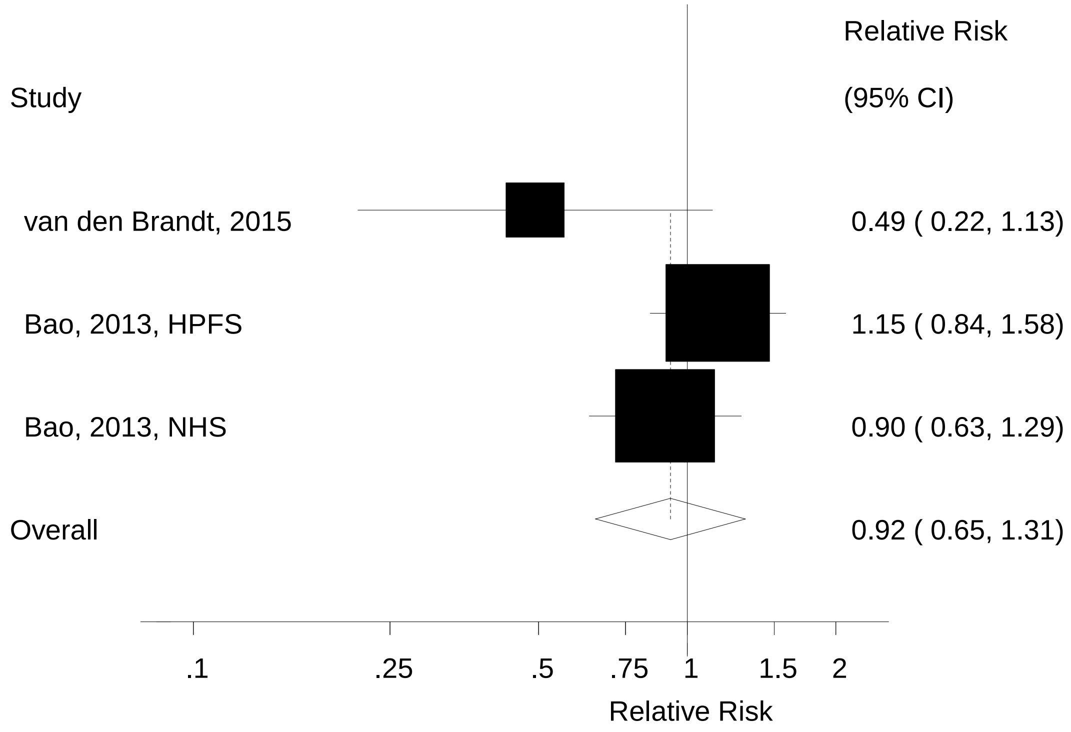

Supplementary Figure 61. Peanuts and neurodegenerative disease mortality, nonlinear dose-response analysis

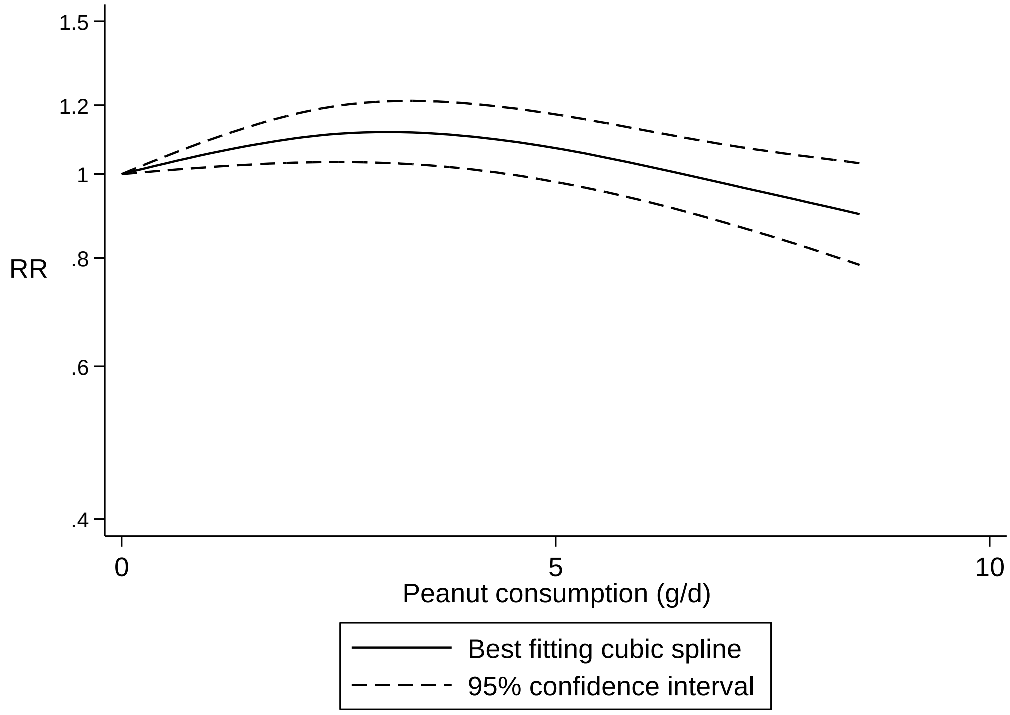

$p_{nonlinearity}$=0.001

Supplementary Figure 62. Tree nuts and infectious disease mortality, high vs. low analysis

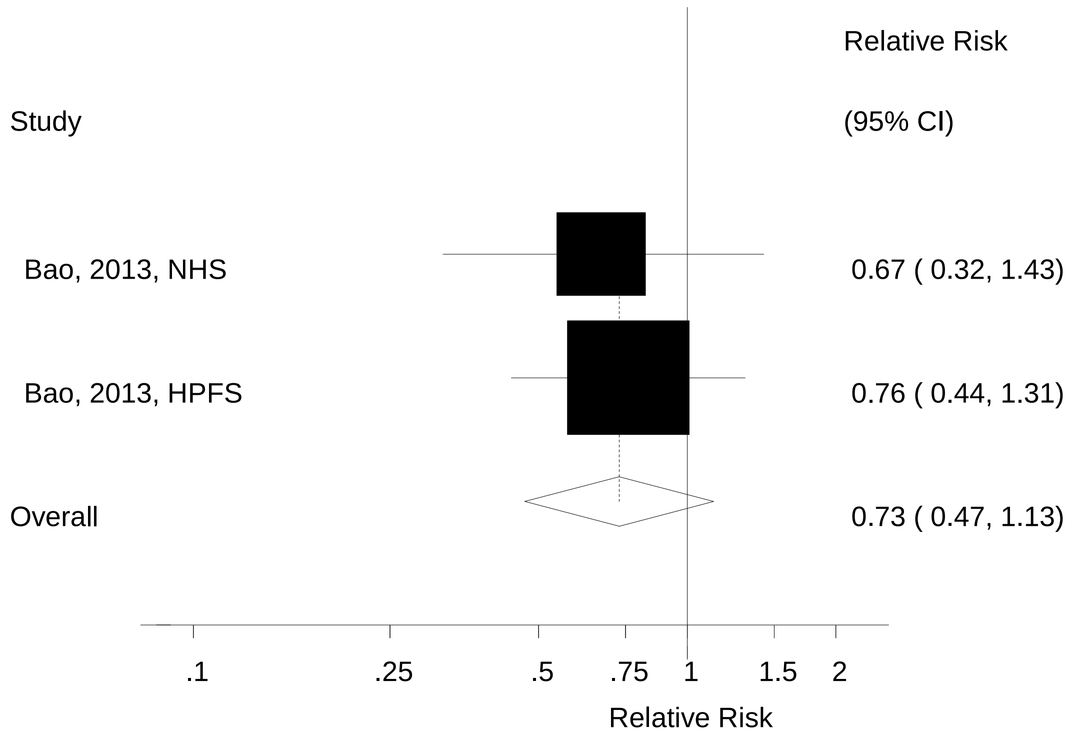

Supplementary Figure 63. Tree nuts and infectious disease mortality, dose-response analysis

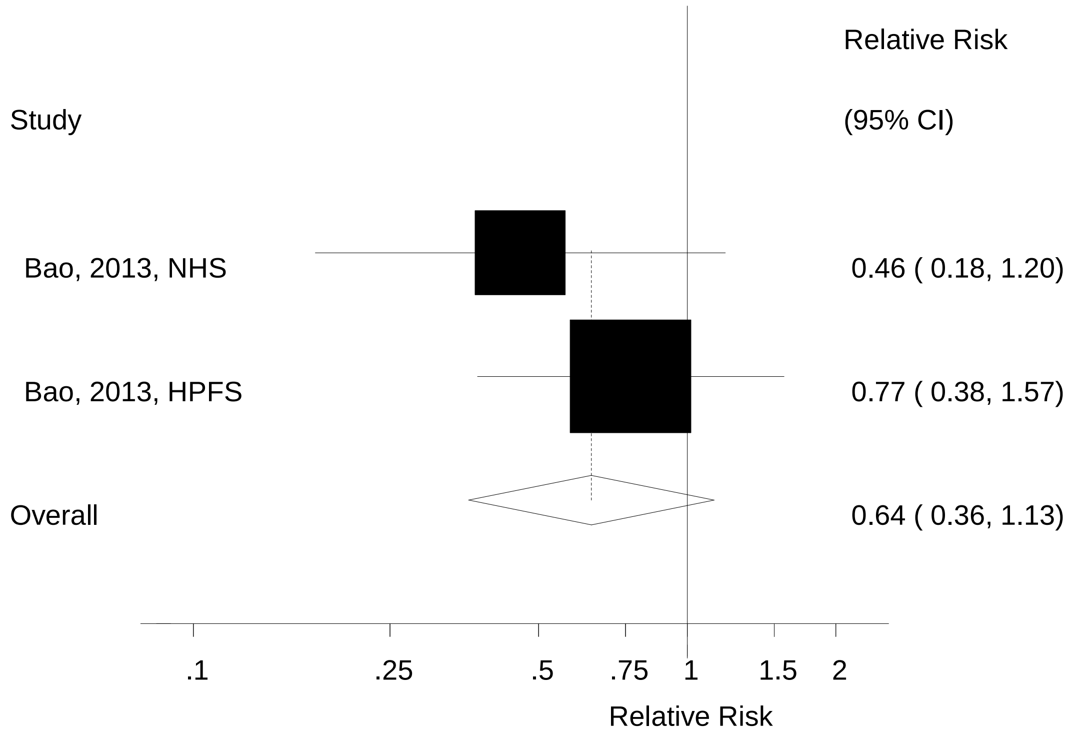

Supplementary Figure 64. Peanuts and infectious disease mortality, high vs. low analysis

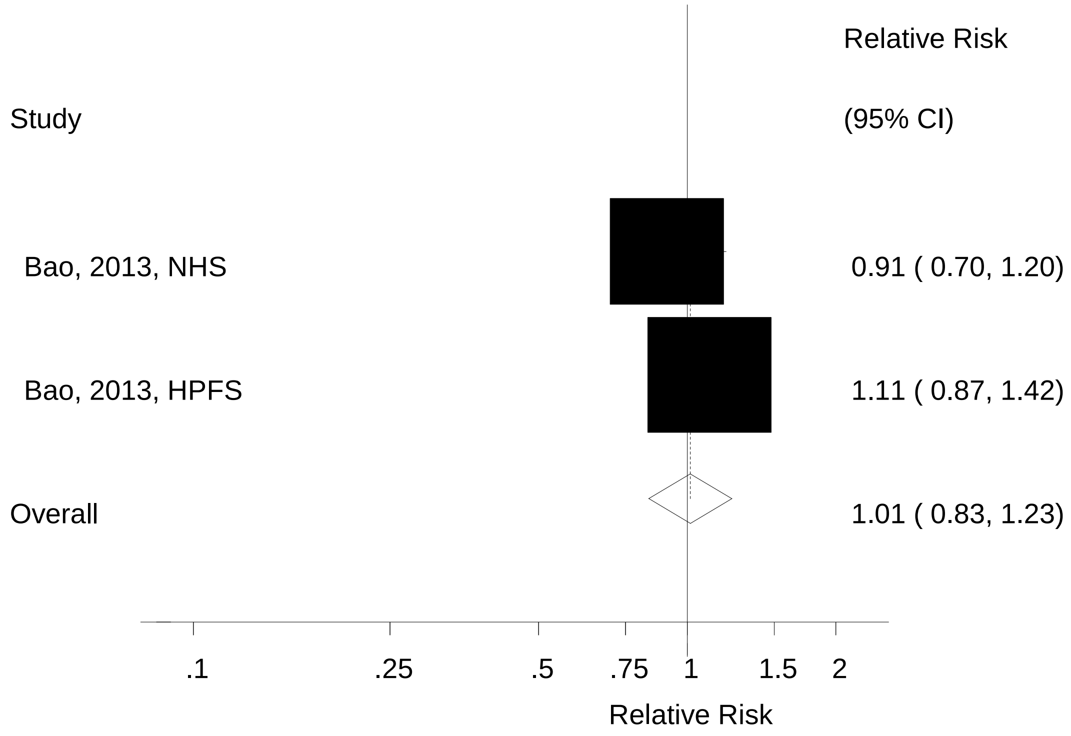

Supplementary Figure 65. Peanuts and infectious disease mortality, dose-response analysis

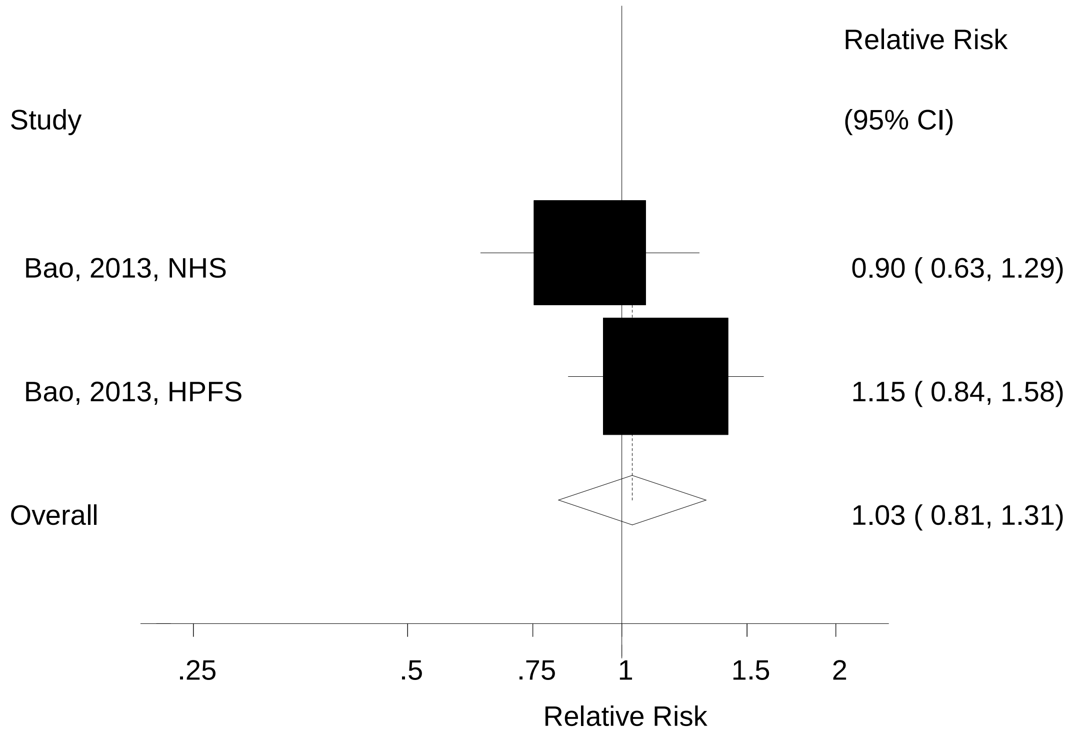

Supplementary Figure 66. Tree nuts and kidney disease mortality, high vs. low analysis

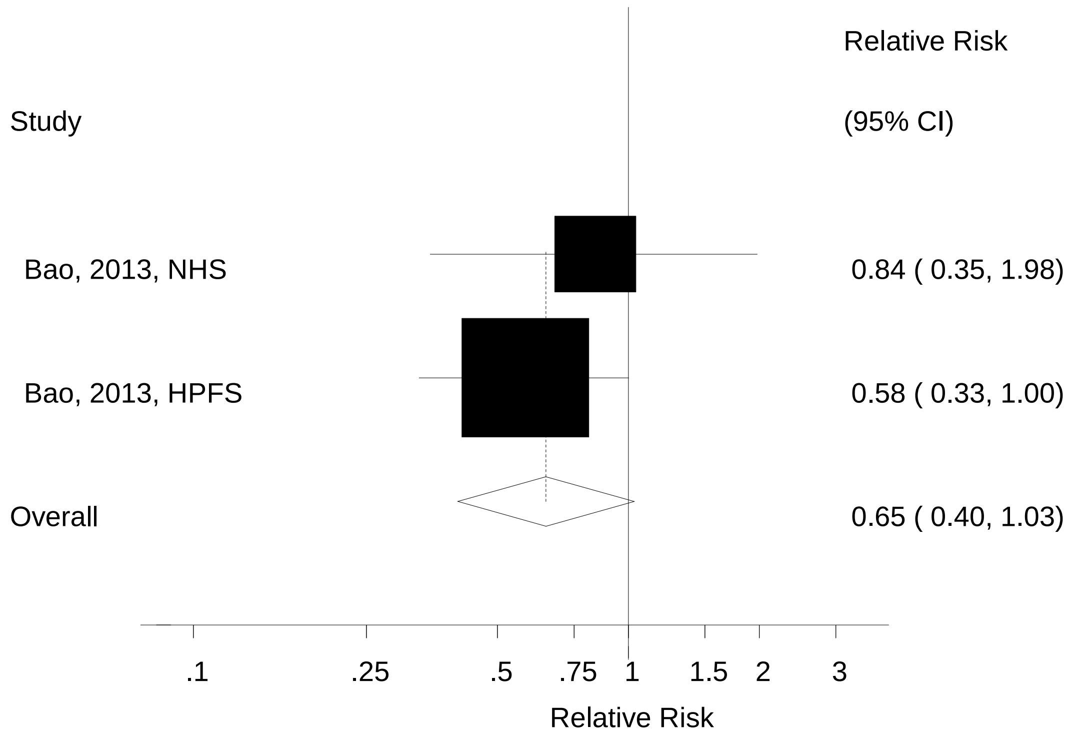

Supplementary Figure 67. Tree nuts and kidney disease mortality, dose-response analysis

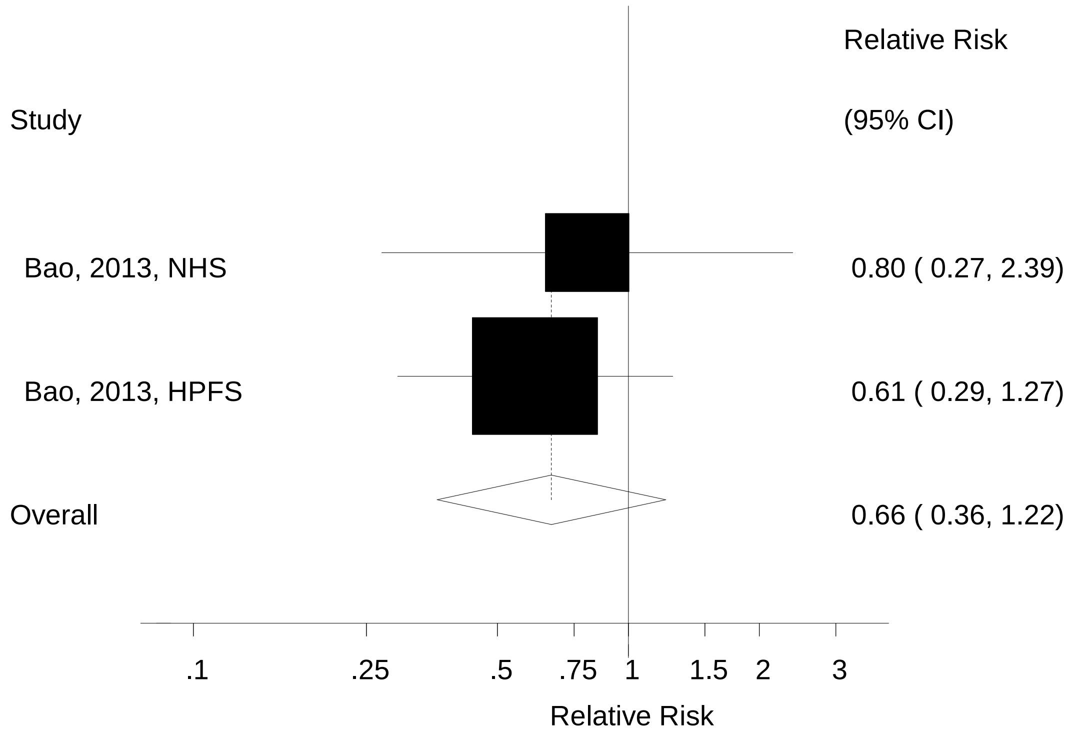

Supplementary Figure 68. Peanuts and kidney disease mortality, high vs. low analysis

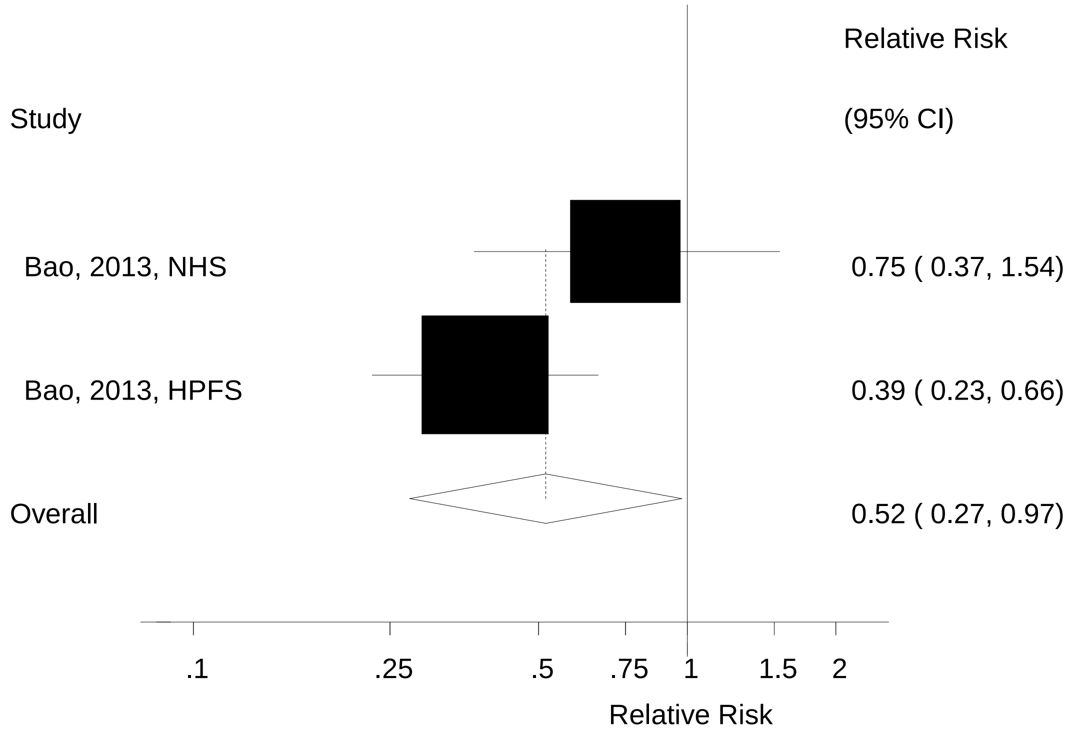

Supplementary Figure 69. Peanuts and kidney disease mortality, dose-response analysis

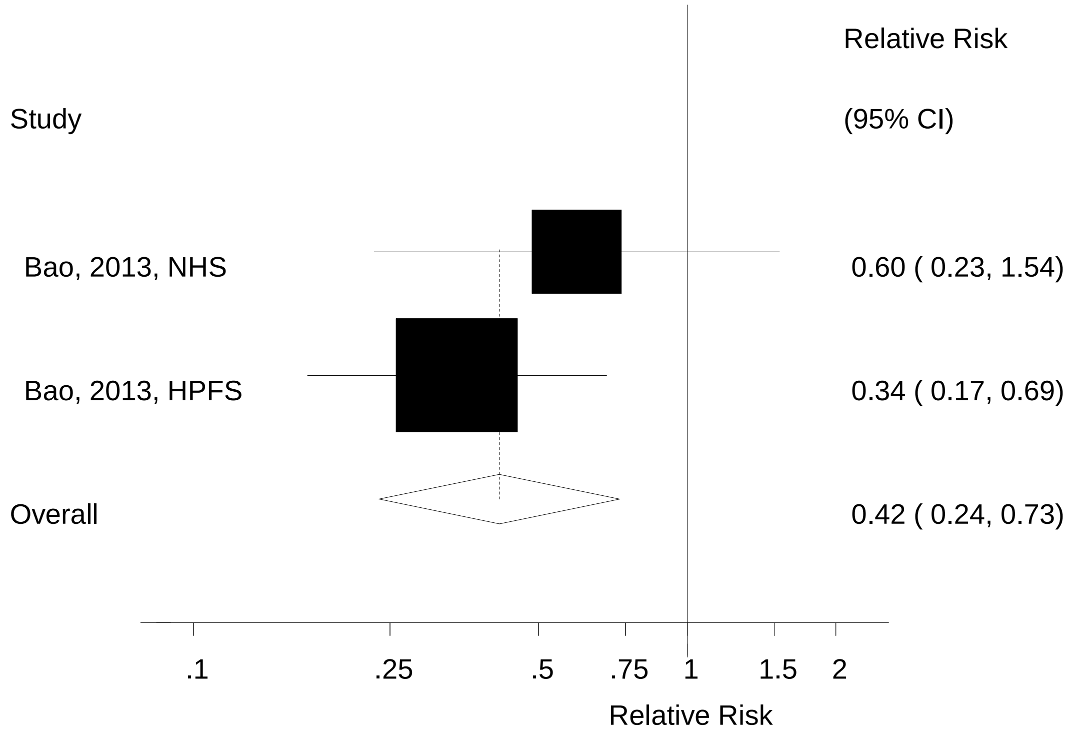

Supplementary Figure 70. Influence analysis of nuts and coronary heart disease

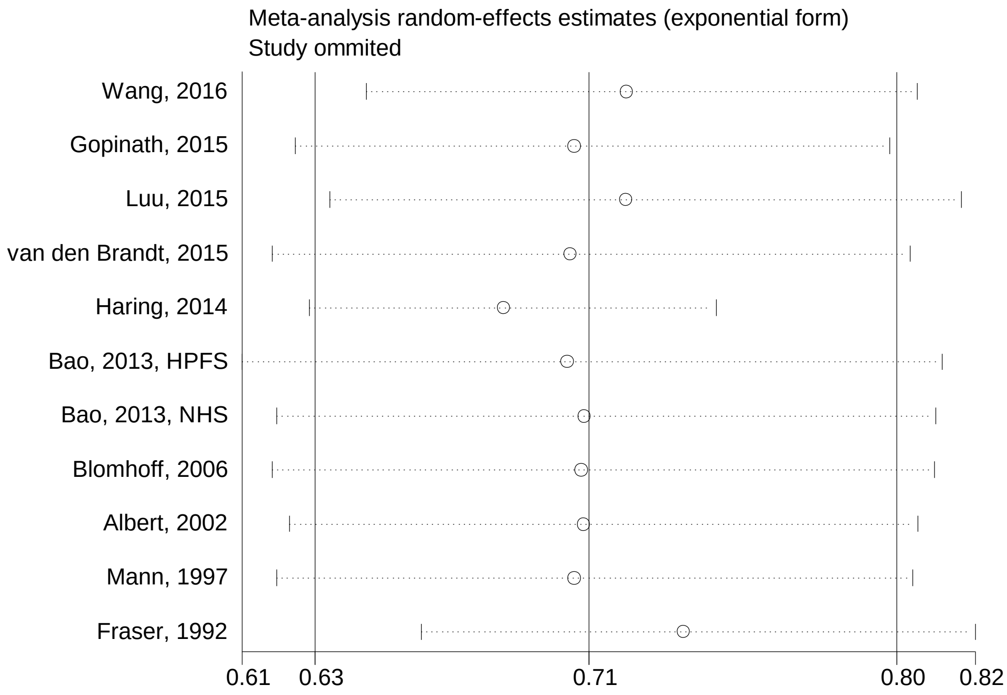

Study omitted	e^coef.	[95% Conf.	Interval]
Wang, 2016	0.72052968	0.64619869	0.80341089
Gopinath, 2015	0.70566195	0.62594962	0.79552531
Luu, 2015	0.72032356	0.63579094	0.81609529
van den Brandt, 2015	0.70455873	0.61944151	0.80137181
Haring, 2014	0.68555653	0.62993771	0.74608612
Bao, 2013, HPFS	0.70361555	0.61073756	0.81061792
Bao, 2013, NHS	0.70846039	0.62067235	0.80866516
Blomhoff, 2006	0.70759284	0.61933184	0.80843192
Albert, 2002	0.70834136	0.6243099	0.80368328
Mann, 1997	0.70556635	0.62062329	0.80213529
Fraser, 1992	0.7367267	0.6618399	0.8200869
Combined	0.7097442	0.63154736	0.7976232

Supplementary Figure 71. Influence analysis of nuts and stroke

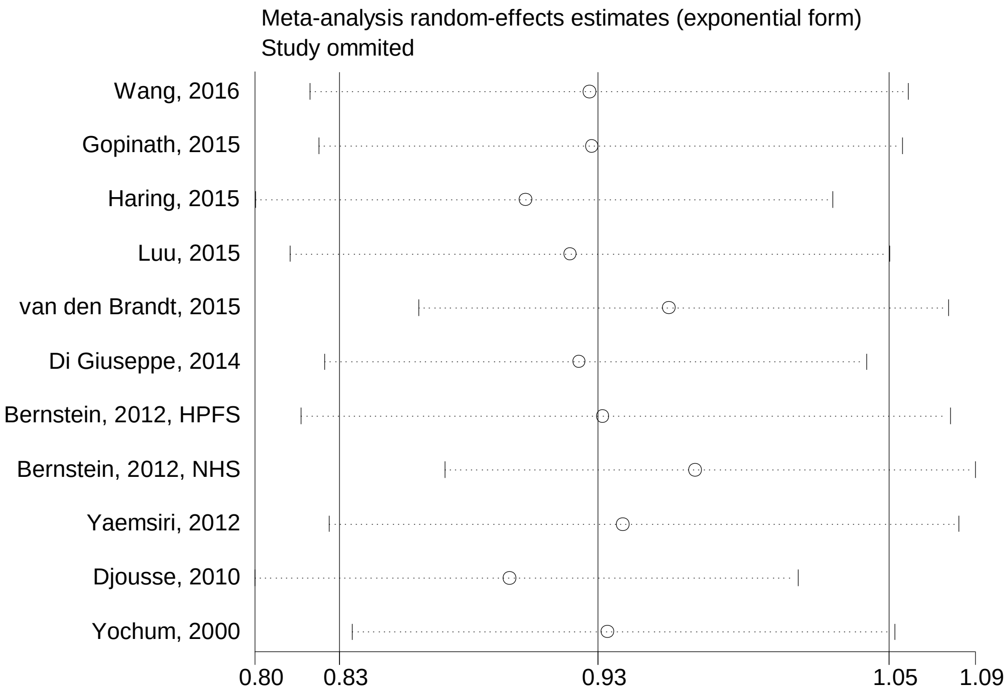

Study omitted	e^coef.	[95% Conf.	Interval]
Wang, 2016	0.93132597	0.81784934	1.0605475
Gopinath, 2015	0.93230218	0.82144582	1.0581189
Haring, 2015	0.90527499	0.79572052	1.0299129
Luu, 2015	0.92347413	0.80986726	1.0530176
van den Brandt, 2015	0.96344882	0.86191368	1.0769449
Di Giuseppe, 2014	0.92721111	0.82383013	1.0435653
Bernstein, 2012, HPFS	0.93673015	0.8142845	1.0775882
Bernstein, 2012, NHS	0.97423249	0.87249619	1.0878316
Yaemsiri, 2012	0.94484818	0.82571584	1.0811687
Djousse, 2010	0.89890766	0.79535866	1.0159378
Yochum, 2000	0.93863279	0.83501011	1.0551146
Combined	0.93461243	0.82972686	1.0527566

Supplementary Figure 72. Influence analysis of nuts and cardiovascular disease

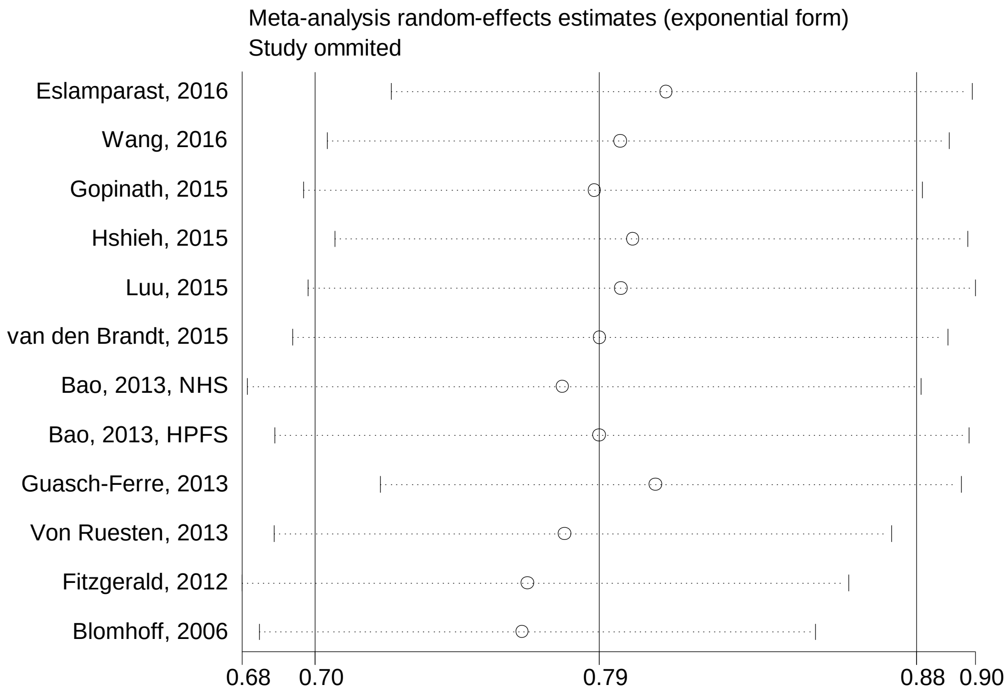

Study omitted	e^coef.	[95% Conf.	Interval]
Eslamparast, 2016	0.80613863	0.7246452	0.89679682
Wang, 2016	0.79246283	0.70572001	0.88986754
Gopinath, 2015	0.78501511	0.69876647	0.88190943
Hshieh, 2015	0.7962243	0.70803815	0.89539397
Luu, 2015	0.79272896	0.70005506	0.89767116
van den Brandt, 2015	0.78652287	0.69546199	0.88950682
Bao, 2013, NHS	0.77547973	0.68212789	0.88160712
Bao, 2013, HPFS	0.78623897	0.69010103	0.89576983
Guasch-Ferre, 2013	0.80284512	0.72145021	0.89342302
Von Ruesten, 2013	0.77608901	0.69001567	0.87289923
Fitzgerald, 2012	0.7650305	0.68047196	0.86009663
Blomhoff, 2006	0.7635538	0.68561733	0.85034949
Combined	0.78620925	0.7021711	0.88030537

Supplementary Figure 73. Influence analysis of nuts and total cancer

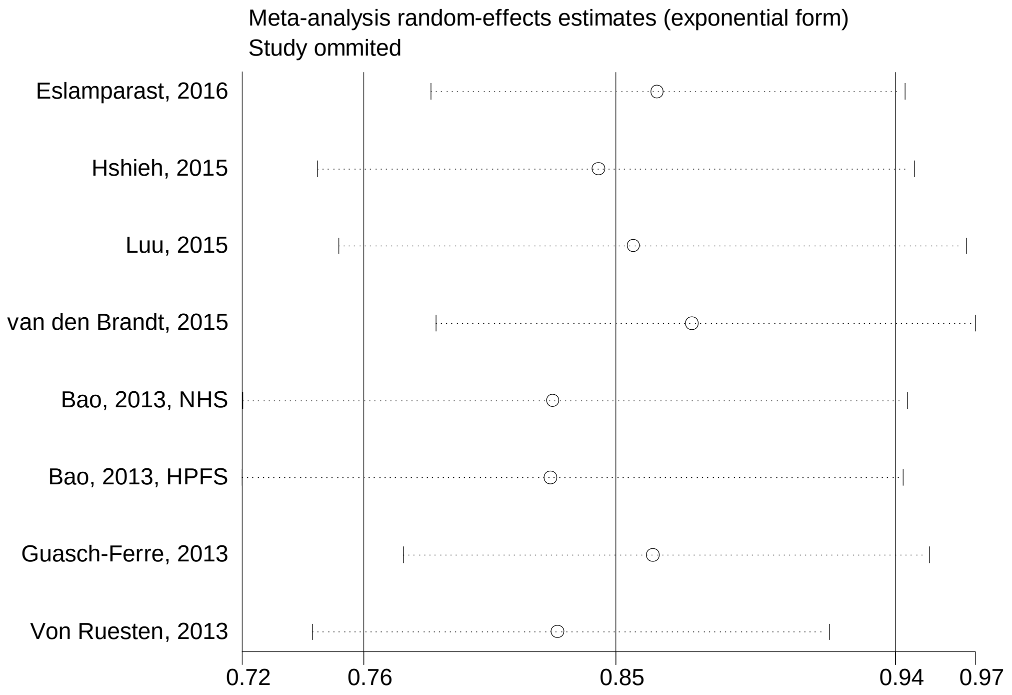

Study omitted	e^coef.	[95% Conf.	Interval]
Eslamparast, 2016	0.86147064	0.78639811	0.94370985
Hshieh, 2015	0.84202188	0.7488178	0.94682688
Luu, 2015	0.85358477	0.75579917	0.9640218
van den Brandt, 2015	0.87291747	0.78801918	0.9669624
Bao, 2013, NHS	0.82687312	0.72396338	0.94441128
Bao, 2013, HPFS	0.82614875	0.72376835	0.94301134
Guasch-Ferre, 2013	0.86000115	0.77724576	0.95156777
Von Ruesten, 2013	0.82846338	0.74715799	0.91861635
Combined	0.84767489	0.76404364	0.94046031

Supplementary Figure 74. Influence analysis of nuts and all-cause mortality

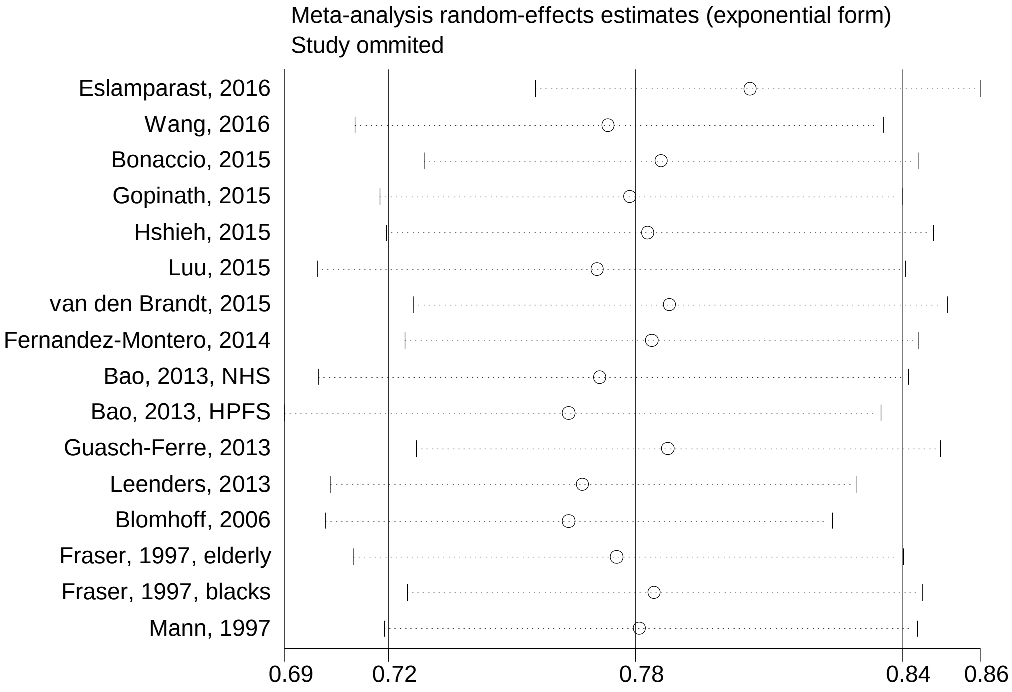

Study omitted	e^coef.	[95% Conf.	Interval]
Eslamparast, 2016	0.80678028	0.75485545	0.86227697
Wang, 2016	0.77253097	0.71132797	0.83899987
Bonaccio, 2015	0.78536564	0.72797155	0.84728473
Gopinath, 2015	0.77780688	0.71734804	0.84336126
Hshieh, 2015	0.78214961	0.71892047	0.85093969
Luu, 2015	0.76988757	0.70216417	0.84414291
van den Brandt, 2015	0.78726429	0.72540563	0.85439795
Fernandez-Montero, 2014	0.78295243	0.72332704	0.84749287
Bao, 2013, NHS	0.77043462	0.70251775	0.84491742
Bao, 2013, HPFS	0.76292157	0.69436026	0.83825266
Guasch-Ferre, 2013	0.78682935	0.72611767	0.8526172
Leenders, 2013	0.76631898	0.70552975	0.83234578
Blomhoff, 2006	0.76296216	0.70423567	0.82658589
Fraser, 1997, elderly	0.77449507	0.71100211	0.84365797
Fraser, 1997, blacks	0.78368533	0.72391659	0.84838879
Mann, 1997	0.78010321	0.71841502	0.8470884
Combined	0.77892971	0.71935823	0.84343441
